# Key Connectomes and Synaptic‐Compartment‐Specific Risk Genes Drive Pathological α‐Synuclein Spreading

**DOI:** 10.1002/advs.202413052

**Published:** 2025-05-28

**Authors:** Yuanxi Li, Justin Torok, Shujing Zhang, Jessica Ding, Ning Wang, Courtney Lau, Shruti Kulkarni, Chaitali Anand, Julie Tran, Michael Cheng, Claire Lo, Binbin Lu, Yanzi Sun, Robert Damoiseaux, Xia Yang, Ashish Raj, Chao Peng

**Affiliations:** ^1^ Department of Neurology David Geffen School of Medicine University of California Los Angeles Los Angeles CA 90095 USA; ^2^ Institute for Cognitive Neurodynamics East China University of Science and Technology Shanghai 200237 China; ^3^ School of Mathematics East China University of Science and Technology Shanghai 200237 China; ^4^ Department of Radiology University of California San Francisco San Francisco CA 94117 USA; ^5^ Department of Integrative Biology and Physiology University of California Los Angeles Los Angeles CA 90095 USA; ^6^ Interdepartmental Program of Molecular Cellular and Integrative Physiology University of California Los Angeles Los Angeles CA 90095 USA; ^7^ Smith College Northampton MA 01063 USA; ^8^ Molecular Screening Shared Resource (MSSR) California NanoSystems Institute University of California Los Angeles CA 90095 USA; ^9^ Institute for Quantitative and Computational Biosciences University of California Los Angeles CA 90095 USA; ^10^ Molecular Biology Institute University of California Los Angeles Los Angeles CA 90095 USA; ^11^ Brain Research Institute University of California Los Angeles Los Angeles CA 90095 USA; ^12^ Mary S. Easton Center for Alzheimer's Research University of California Los Angeles Los Angeles CA 90095 USA

**Keywords:** key connectomes, mathematical models, pathological α‐synuclein, risk genes, systems biology analyses

## Abstract

Previous studies have suggested that pathological α‐synuclein (α‐Syn) mainly transmits along the neuronal network, but several key questions remain unanswered: 1) How many and which connections in the connectome are necessary for predicting the progression of pathological α‐Syn? 2) How to identify risk genes that affect pathology spreading functioning at presynaptic or postsynaptic regions, and are these genes enriched in different cell types? Here, these questions are addressed with novel mathematical models. Strikingly, the spreading of pathological α‐Syn is predominantly determined by the key subnetworks composed of only 2% of the strongest connections in the connectome. Genes associated with the selective vulnerability of brain regions to pathological α‐Syn transmission are further analyzed to distinguish those functioning at presynaptic versus postsynaptic regions. Those risk genes are significantly enriched in microglial cells of presynaptic regions and neurons of postsynaptic regions. Gene regulatory network analyses are then conducted to identify “key drivers” of genes responsible for selective vulnerability and overlapping with Parkinson's disease risk genes. By identifying and discriminating between key gene mediators of transmission operating at presynaptic and postsynaptic regions, this study has demonstrated for the first time that these are functionally distinct processes.

## Graphical abstract

Utilizing a stereotaxic injection mouse model and a novel mathematical approach, this study uncovers key subnetworks that drive pathological α‐synuclein (α‐Syn) progression in Parkinson's disease (PD). Remarkably, just 2% of the strongest connections in the connectome are sufficient to predict its spread. The study also demonstrated that distinct risk genes and cell types regulate α‐Syn transmission at different synaptic sites.

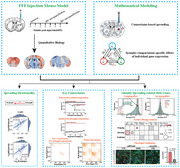

## 1 Introduction

Parkinson's disease (PD) is characterized by the accumulation of misfolded α‐synuclein (α‐Syn) in the central nervous system (CNS).^[^ ^1^, ^2^, ^3^ ^]^ Previous evidence suggests that PD progression is driven by the intercellular spreading and templated amplification of pathological α‐Syn in the CNS.^[^ ^1^, ^4^, ^5^ ^]^ Hence, blocking either the spreading or amplification of pathological α‐Syn is promising therapeutic strategies for PD.^[^ ^2^ ^]^ Injecting misfolded α‐Syn preformed fibrils (PFFs, as the “pathological seeds”) into the brains of wild‐type (WT) mice can induce misfolding of native α‐Syn into pathological conformations, which then spreads to other brain regions.^[^ ^6^ ^]^ This injection mouse model for PD research is ideal for studying the spreading of pathological α‐Syn along the neuronal network.^[^ ^6^, ^7^ ^]^

Biophysical or mathematical models of network spreading have become useful tools to augment experimental studies. Such models can facilitate our understanding of rich experimental data by helping to validate specific hypotheses about disease mechanisms that are difficult to observe directly. Biophysical models can further reveal which processes control disease dynamics, and uncover key mechanisms that future treatments could be designed to disrupt.^[^ ^8^ ^]^ Mathematical models, especially the network diffusion model,^[^ ^9^ ^]^ the Smoluchowski network model,^[^ ^10^, ^11^, ^12^ ^]^ and the Nex*is* model,^[^ ^13^ ^]^ which rely upon the mesoscale connectome^[^ ^14^, ^15^ ^]^ and Allen Gene Expression Atlas (AGEA),^[^ ^16^ ^]^ have been used in silico to model and predict the progression of pathological proteins. Pathological α‐Syn progression was modeled based on the following factors: pathology amplification and clearance, network spread, and regional selective vulnerability explained by individual gene expression.^[^ ^7^, ^13^, ^17^, ^18^ ^]^

Despite the progress made by previous studies,^[^ ^7^, ^17^, ^18^, ^19^, ^20^ ^]^ the following important questions remain unclear: 1) Previous models are based on the hypothesis that pathological α‐Syn spreading is either fully anterograde or fully retrograde in WT mice and *G2019S LRRK2* transgenic mice.^[^ ^7^ ^]^ However, bidirectional spreading with both retrograde and anterograde components is likely but has not been investigated in previous studies. What are the proportions of the anterograde spreading and retrograde spreading? 2) Is there any difference in pathological α‐Syn spreading directionality preference between WT mice and *G2019S LRRK2* transgenic mice? 3) All previous studies have used the whole or the majority of the whole mesoscale connectome to model the spreading of pathological α‐Syn. It remains unknown how many connections from the whole connectome are necessary for predicting the pathological α‐Syn progression. In other words, are there subsets of key connections in the actual connectome that predominantly determine the spreading patterns of pathological α‐Syn? 4) In previous studies, genes that contribute to the selective vulnerability of pathological α‐Syn spreading were identified based on the assumption that they only function in presynaptic regions (referred to as the “outgoing connections”; see Equation (9) in the Experimental Section). However, it is possible that distinct genes modulate pathological α‐Syn spreading in postsynaptic regions specifically (referred to as the “incoming connections”); or in both the presynaptic and postsynaptic regions. How to model and identify these different kinds of genes? 5) Both neurons and glial cells have been shown to modulate the spreading of pathological proteins.^[^ ^2^ ^]^ It is unknown which cell type predominantly encodes the selective vulnerability of pathological α‐Syn spreading.

Here, we quantitatively measured the spatiotemporal patterns of pathological α‐Syn in PFF‐injected WT mice at 3 and 6 months‐post‐injection (MPI) at a higher resolution than the previous study.^[^ ^7^ ^]^ These data and an external dataset from a previous study^[^ ^7^ ^]^ were then coupled with the augmented Nex*is* model^[^ ^13^ ^]^ (see Equations (2)–(8) in the Experimental Section) and systems biology approach (see Systems Biology Analyses subsections in the Experimental Section) to address the questions above. Compared to the previous mathematical models in this field,^[^ ^7^, ^9^, ^10^, ^11^, ^12^, ^13^, ^17^, ^21^ ^]^ our augmented Nex*is* model was able to simultaneously capture the seed scaling, the global amplification and clearance effect, the diffusion effect, and the directionally biased spread of pathology, whereas the others were only modeling parts of the above factors, especially ignoring the possibility of directionally biased spread. We found that pathological α‐Syn transmitted predominantly retrogradely in WT mice, but the directionality of pathological α‐Syn spreading in *G2019S LRRK2* mice was bidirectional with a slight anterograde bias. This is the first report on the quantitative proportion of pathological α‐Syn spreading and how *G2019S LRRK2* affects pathological α‐Syn spreading directionality, giving a more nuanced picture of the directional preference of α‐Syn spreading than previously assumed.^[^ ^7^, ^17^, ^18^, ^20^ ^]^ Unexpectedly, we then found that the spread of pathological α‐Syn can be predicted using as few as the top 2% strongest connections of the whole mesoscale connectome. This surprising finding suggests that the spreading of pathological α‐Syn is predominantly determined by key connectomes composed of a minor subset of brain regions and connections, which may guide the investigations in this field focusing on these “key connectomes”. We also identified candidate risk genes that modulated pathological α‐Syn spreading by affecting the presynaptic regions specifically, postsynaptic regions specifically, and both (see Equation (9) in the Experimental Section). We performed systems biology analyses to study the similarities and differences of top risk genes ranked by our model performance. We found that the risk genes of outgoing effect were enriched in microglial cells, while the risk genes of incoming effect were enriched in neurons, indicating that different cell types play distinct roles in determining the selective vulnerability at presynaptic versus postsynaptic sites. Gene regulatory network analyses were then conducted to identify “key drivers” of genes responsible for selective vulnerability and overlapping with Parkinson's disease risk genes. Subsequently, to further validate our findings, we selected *RORA*, one of the top‐ranked genes in terms of incoming effect, for functional validation, and provided the first evidence that *RORA* is involved in regulating pathological α‐Syn spreading, specifically exerting an inhibitory effect. Overall, our study successfully investigated the key connectomes and risk genes at different synaptic sites in modulating α‐Syn pathology progression. Another significant aspect of our study is that it establishes a pipeline for computationally identifying vulnerable genes related to pathology spreading and biologically validating their functions, which could serve as a valuable framework for research on other neurodegenerative diseases.

## 2 Results

### 2.1 Quantitative Pathology in the Stereotaxic Injection Mouse Model

**Figure 1:**
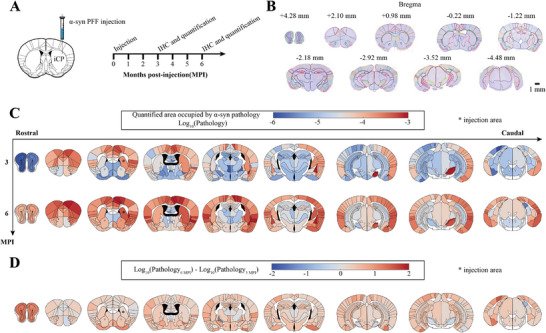
Immunohistochemistry experiments and quantitative biology. A) Experiment schematic: pathological α‐Syn PFF was stereotaxically injected into the iCP region. Mice were euthanized at 3 and 6 months‐post‐injection prior to immunohistochemistry, which were then quantitatively assessed for α‐Syn inclusions using QuPath. B) Representative images of brain sections and annotations. 9 brain bregma levels were selected per mouse, and 196 gray matter regions were manually annotated. C) Heatmap of regions affected by α‐Syn pathologies (log10‐transformed; *, injection area). Warmer colors represent areas with higher pathology burdens, while cooler colors represent areas with lower pathology burdens. D) Differences of regional pathology burdens between 3 and 6 MPI, where the color indicates the fold change in regional pathology burden between 3 and 6 MPI. Scale bar in (B): 1 mm.

We used the stereotaxic injection mouse model to study the progression of pathological α‐Syn in the CNS.^[^ ^6^ ^]^ Pathological α‐Syn PFF was stereotaxically injected into the ipsilateral dorsal striatum (ipsilateral caudoputamen region, iCP) of two‐to‐three‐month‐old C57BL6/C3H wild‐type mice to mimic the progression of α‐Syn pathology. Mice were euthanized at 3 and 6 MPI for the immunohistochemistry analyses (**Figure** **1**A). α‐Syn pathology was revealed by staining with antibody against pathological α‐Syn (Syn506).^[^ ^1^ ^]^ Stained brain slices at 9 specific bregma levels were selected per mouse and annotated according to the Allen Mouse Brain Reference Atlas (AMBA, https://mouse.brain‐map.org/static/atlas). The QuPath software was then used to quantify the percentage area occupied by α‐Syn pathology in 196 brain gray matter regions (Figure 1B). With the updated AMBA database, we were able to achieve a higher resolution.^[^ ^7^ ^]^

The spatiotemporal distributions of α‐Syn pathology are shown in Figure 1C (see also Table S1 in the Supporting Information). Similar to previous findings,^[^ ^1^, ^7^ ^]^ the ipsilateral side had more pathology than the contralateral side (Figure 1C). Comparisons between 3 and 6 MPI (Figure 1D) showed distinct pathological burden changes in subregions of hippocampus, prefrontal cortex, and midbrain. As reported before, decreased pathology burden was observed in the substantia nigra (SN) between 3 and 6 MPI.^[^ ^6^, ^22^ ^]^ With our higher annotation resolution, we also found reduced pathology in the ipsilateral postsubiculum (iPOST; Figure 1D and Figure S1 (Supporting Information)), but the vast majority of regions showed increased α‐Syn burdens between 3 and 6 MPI (Figure 1D). We also identified a significant increase in pathology burden in the ipsilateral olfactory areas (iOLF), the primary motor area (iMOp), the ipsilateral primary somatosensory area, lower limb (iSSp‐ll), and the contralateral caudoputamen (cCP) (Figures S1–S3 and Table S1, Supporting Information).

### 2.2 Different Directionality Preferences of Pathological α‐Syn Spreading in WT Mice and *G2019S LRRK2* Transgenic Mice

A very important question regarding pathology spread is the preference of direction (anterograde vs retrograde). Previous mathematical models of pathological α‐Syn spreading have only compared the situation of fully anterograde transport and fully retrograde transport, and concluded that pathological α‐Syn spreading is due to fully retrograde transport in both WT mice and *G2019S LRRK2* mice (see Equation (5) in the Experimental Section)^[^ ^7^, ^17^, ^18^ ^]^; however, while both anterograde and retrograde transport have been reported for pathological α‐Syn,^[^ ^23^ ^]^ unequal bidirectional spread has not been previously explored in mathematical models.^[^ ^7^, ^17^, ^18^, ^20^ ^]^

We used Nex*is*:global^[^ ^13^ ^]^ as the global spread model to investigate the pathological progression including the amplification, clearance, and spreading of pathological α‐Syn (see Equations (2)–(8) in the Experimental Section for details). To accommodate a continuous measure of net directional preference, the original Nex*is*:global model^[^ ^13^ ^]^ was augmented by introducing a new parameter, *s*, to indicate spreading direction. *s* is bounded between 0 and 1, with 0 indicating fully anterograde spread and 1 indicating fully retrograde spread (see Equations (2)–(8) in the Experimental Section for details). To the best of our knowledge, our model has incorporated and modeled the spread directionality preference as a factor in the progression of pathological α‐Syn for the first time. We used concordance correlation coefficients averaged over 3 and 6 MPI (abbreviated as Ave. CCC in the following paragraphs) as the loss function to fit our model because it can capture the actual values of the data as well as the trend (scale).^[^ ^24^ ^]^

**Figure 2:**
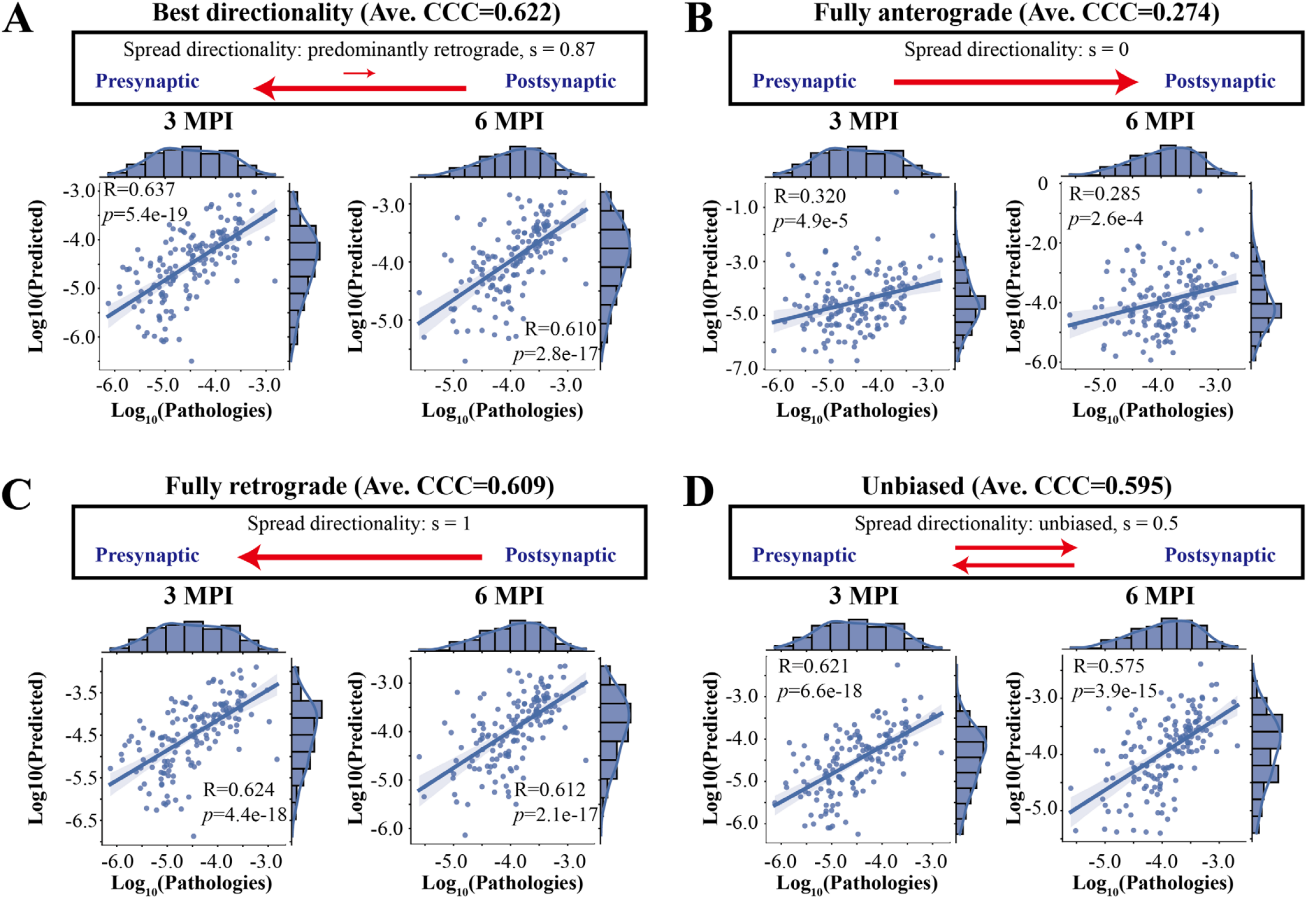
Global spread model based on the mesoscale connectome revealed the spread directionality of α‐Syn in WT mice was predominantly retrograde with a minor anterograde component. A) Best model results (Ave. CCC = 0.622) with the directionality parameter of *s* = 0.87. B) Model results (Ave. CCC = 0.274) with the fully anterograde parameter of *s* = 0. C) Model results (Ave. CCC = 0.609) with the fully retrograde parameter of *s* = 1. D) Model results (Ave. CCC = 0.595) with the unbiased directionality parameter of *s* = 0.5. The directionality of pathological α‐Syn spread is shown by the arrows in the box, while the length of the line indicates the proportion of spread direction. Each dot represents one brain region, and the *x*‐axis and *y*‐axis represent the pathology (log10‐transformed) found empirically and predicted by the model, respectively. For each situation, the Pearson's correlation coefficient and the best regression lines for both 3 and 6 MPI are also displayed. The shaded ribbon represented the 95% prediction interval. Abbreviations: *R*, Pearson's correlation coefficient; *p*, *p* values from linear regression.

Our new model demonstrates that pathological α‐Syn mainly spreads retrogradely with minor anterograde spreading (*s* = 0.87, Ave. CCC = 0.622) in WT mice, and the overall balance between amplification and clearance was weighted toward amplification, indicated by its positive sign (α = 0.33, see the Experimental Section) (**Figures** **2**A and S4 (Supporting Information)). We then tested our model under three directionality cases: fully anterograde, fully retrograde, and equal amount of anterograde versus retrograde spread (unbiased spread) (Figure 2B–D), which all had lower Ave. CCC values than the best directionality condition. To compare with previous methods utilizing Pearson's correlation coefficient (Pearson's *R*) as the target metric,^[^ ^7^, ^9^, ^18^, ^25^ ^]^ we found that the average Pearson's *R* for our model was approximately the same as Ave. CCC, indicating that our model captures the scale of the observed data (Figure 2A). Moreover, the model of previous studies^[^ ^7^, ^18^ ^]^ disregarded the amplification process of pathology and the unequally directional transmission, which significantly compromised the ability to predict both trend and scale compared to our current model (Figure S5, Supporting Information). Our model also accurately captured the disproportionate pathological α‐Syn deposition in the ipsilateral hemisphere relative to the contralateral hemisphere at both 3 and 6 MPI (Figure S6, Supporting Information).

To externally validate our observation that pathological α‐Syn mainly spreads retrogradely in WT mice, we then tested the spread directionality using our model and previous datasets from  Henderson et al.,^[^ ^7^ ^]^ which consisted of two groups of mice (wild‐type nontransgenic mice and *G2019S LRRK2* transgenic mice; see the Experimental Section for further details). In agreement with the results above, our model predicted that pathological α‐Syn also spreads predominantly retrogradely in these WT mice (Figure S7A (Supporting Information); Ave. CCC = 0.599, *s* = 0.75). More interestingly, we found that the directionality of pathological α‐Syn spread in *G2019S LRRK2* transgenic (*G2019S*) mice was very different from that in the WT mice, showing bidirectional spread with a slight anterograde preference (Figure S7B, Supporting Information; Ave. CCC = 0.561, *s* = 0.40). We also tested the previous hypothesis of retrograde spread^[^ ^7^ ^]^ in *G2019S LRRK2* mice (Figure S7C, Supporting Information; Ave. CCC = 0.508, *s* = 1), which performed worse than the best directionality model (Figure S7B, Supporting Information). We conclude that the *G2019S LRRK2* modification has a dramatic effect on the directionality of pathological α‐Syn spread. In addition, we evaluated the performance of our model against the previous model^[^ ^7^ ^]^ on both datasets, and the results demonstrated that our model outperformed theirs (Figures S8 and S9, Supporting Information).

### 2.3 The Spread of Pathological α‐Syn Is Predicted Using Only 2% Connections of the Whole Connectome

**Figure 3:**
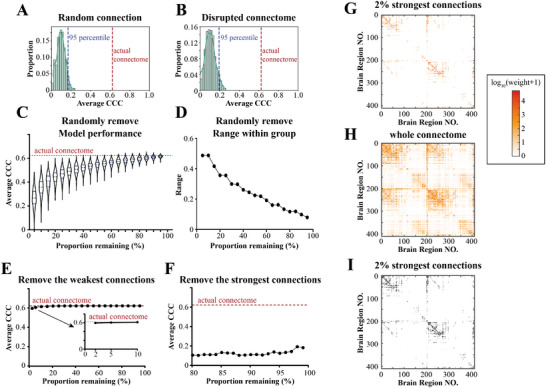
The spread of pathological α‐Syn was driven by the key connectomes composed of only top 2% strongest connections. A) Distribution of model fitting results using 1000 matrices with entries sampled from the standard uniform distribution in place of the actual connectome (Ave. CCC at 95th percentile = 0.169). B) Distribution of model fitting results using matrices with randomly permuted elements of the actual connectome (Ave. CCC at 95th percentile = 0.194). C,D) Distributions of model fitting results by removing random subsets of connections of various sizes at a 5% incremental gradient, resampling the connectome 1000 times per subset size. (C) Showing the model performance: for each, the dashed line represented the median, and the two solid lines represented the 25% and 75% percentiles. (D) Showing the range (maximum minus minimum Ave. CCC value) within the group. E,F) Model fitting results after removing proportions of edges from the connectome in order of connection strength. (E) Model fitting results after removing from weakest connections; note that the model did not fit successfully when removing 99% of the weakest connections. (F) Model fitting results after removing the strongest connections. The dotted line of the actual connectome showed the best model fitting of the global spread model with the actual (whole) connectome (Ave. CCC = 0.622). G–I) Visualization of key connectomes (2% of the strongest connections), showing heatmaps of the connection values, where the warmer colors represent stronger connections (log10(weight + 1)‐transformed). (G) Showing 2% of the strongest connections of the connectome. (H) Showing the whole connectome. (I) Heatmap of the adjacency matrix corresponding to (G). The actual connectome here consists of 410 brain regions with 168 100 connections, and 2% of connectome equals to 3362 connections.

To evaluate whether the actual connectome best predicts the spreading of pathological α‐Syn, we tested if random artificial connectomes (“null models”) could predict pathological α‐Syn progression. We fit our data using connectomes with elements sampled from a uniform random distribution, with 1000 replicates to demonstrate the robustness. As expected, the actual connectome predicted pathological α‐Syn spreading much better than the randomly generated matrices (**Figure** **3**A). Similarly, we tested the case when elements of the whole connectome were randomly permuted; the actual connectome also outperformed the permuted connectomes (Figure 3B). These results demonstrate that pathological α‐Syn spreads along the actual mesoscale connectome.

Previous studies have used the whole or a majority of the whole mesoscale connectome to predict the spreading of pathological α‐Syn.^[^ ^7^, ^17^, ^18^, ^19^, ^20^ ^]^ However, it remains unknown whether the connections in the connectome contribute equally to the spreading of pathological α‐Syn, or if the spreading of pathological α‐Syn is predominantly determined by a subset of the connections. To test these hypotheses, we randomly removed different proportions (5–95%) of the actual connectome and evaluated how well the remaining partial connectome can predict pathological α‐Syn spreading, repeating each case 1000 times for robustness (Figure 3C). Interestingly, the model worked reasonably well when removing 50–60% of the connections, and could achieve great accuracy in some specific cases even with only 5% of connections remaining (Figure 3C). We also observed much higher variations of the model performance when more connections were removed (Figure 3D). These results suggest that connections in the connectome may not contribute equally. In other words, there should be subnetworks composed of key connections in the connectome which predominantly determine the spreading pattern of pathological α‐Syn.

We hypothesize that the key subnetworks that determine the spreading of pathological α‐Syn are composed of the strongest connections in the connectome. To test this hypothesis, we removed different proportions of the strongest or weakest connections from the connectome and evaluated the effects on our model's performance (Figure 3E,F). Strikingly, using as few as 2% of the strongest connections from the actual connectome (3362 of 168 100 connections), our model still predicted the spreading of pathological α‐Syn almost as well as using the complete connectome (Figure 3E). By contrast, removing only the top 1% of the strongest connections from the connectome dramatically disrupted the performance of the model (Figure 3F). Accordingly, we visualized these connections as well as the whole connectome (Figure 3G–I and Figure S10 (Supporting Information)).

We then validated this observation using the external datasets of WT mice and *G2019S LRRK2* mice (Figure S11, Supporting Information) from Henderson et al.^[^ ^7^ ^]^ As expected, the progression of pathological α‐Syn was well predicted by the 8% of the strongest connections from their “masked” connectome (1077 of 13 456 connections), and removing even the top 1% of the strongest connections greatly decreased model performance (Figure S11, Supporting Information). Note that we used 8% of the strongest connections from their “masked” connectome instead of 2%, because the “masked” connectome used in Henderson et al.^[^ ^7^ ^]^ has ≈13‐fold fewer connections than we used in our study. These findings strongly support our hypothesis that the spreading of pathological α‐Syn is predominantly determined by a small subset of the strongest connections in the brain (Figure 3 and Figure S11 (Supporting Information)).

### 2.4 Distinct Genes Modulate Pathological α‐Syn Spreading on the Presynaptic versus Postsynaptic Site

The spreading pattern of pathological α‐Syn has not been fully predicted by the anatomic connectome as well as the global amplification and clearance effects (Figure 2A). This observation suggests that certain brain regions are selectively vulnerable to pathological α‐Syn accumulation and spreading, which could be caused by the distinct regional gene expression levels.^[^ ^1^, ^7^, ^26^, ^27^ ^]^ To investigate if pathology burden distribution is directly correlated with gene expression levels, gene expression data of 3855 genes from the AGEA coronal series^[^ ^16^ ^]^ were analyzed, and we measured the gene correlations by calculating Pearson's correlation coefficient between individual gene expression vector and pathology burden levels of our WT mice. The results showed that at both 3 and 6 MPI, the global spread model was significantly better than the gene correlation model (Figure S12, Supporting Information), demonstrating that the mesoscale connectome necessary for predicting the progression of pathological α‐Syn.

**Figure 4:**
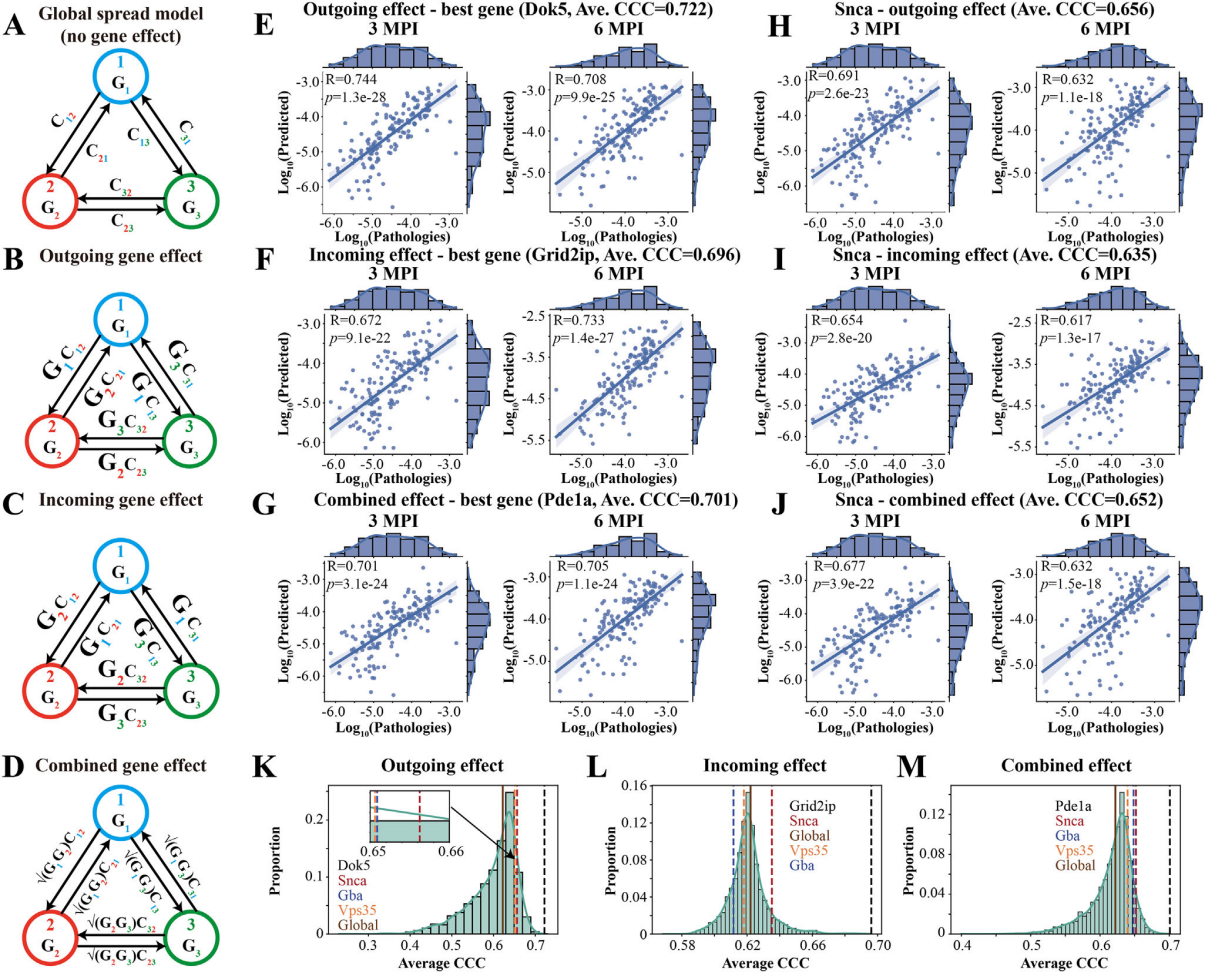
Model results of gene expression model for the outgoing, incoming, and combined effects. A–D) Schematics of the global spread model (A) and gene expression models (B, outgoing effect; C, incoming effect; D, combined effect): the circles in different colors indicates brain regions; *G_i_* in the circles indicate the regional gene expression; and *C_ij_* indicates the axonal projection strength from brain region *i* to *j*. The regional gene expression had different updated effects on the connectome by revising nothing (A, global spread model), revising only the outgoing connections (B, outgoing effect), only the incoming connections (C, incoming effect), or both equally (D, combined effect). E) Model results of the best outgoing effect gene (*Dok5*). F) Model results of the best incoming effect gene (*Grid2ip*). G) Model results of the best combined effect gene (*Pde1a*). H–J) Model results of *Snca* gene for its outgoing (H), incoming (I), and combined effects (J), respectively. K) The distribution of model results of outgoing effect (model performance: *Dok5* > *Snca* > *Gba* > *Vps35* > *Global*). L) The distribution of model results of incoming effect (model performance: *Grid2ip* > *Snca* > *Global* > *Vps35* > *Gba*). M) The distribution of model results of combined effect (model performance: *Pde1a* > *Snca* > *Gba* > *Vps35* > *Global*). Each dot in (E–J) represents one brain region, while the *x*‐axis and *y*‐axis represent the pathology (log10‐transformed) found empirically and predicted by the model, respectively. The Pearson's correlation coefficient and the best regression lines for both 3 and 6 MPI are also displayed. The shaded ribbon in (E–J) represents the 95% prediction interval. Abbreviations: *R*, Pearson's correlation coefficient; *p*, *p* values from linear regression.

We sought to incorporate gene expression in our global spread model to study how connectome and gene expression together contribute to α‐Syn spread. Previous studies to identify genes that contribute to selective vulnerability have only focused on genes that modulate spreading on the outgoing connections at the presynaptic site (see Equation (9) in the Experimental Section).^[^ ^7^, ^17^, ^19^ ^]^ However, the effects of gene expression on the postsynaptic site, and on both the presynaptic and postsynaptic regions, have not been considered yet. To identify genes that affect the outgoing connectome alone (i.e., the “outgoing” effect, which models the effect of gene expression on the presynaptic regions alone), incoming connections alone (i.e., the “incoming” effect, which models the effect of gene expression on the postsynaptic regions alone), or equally on outgoing and incoming connections (i.e., the “combined” effect), we revised and augmented the Nex*is* model.^[^ ^13^ ^]^ Briefly, the original connectome matrix incorporated in the global spread model did not incorporate any individual gene's effect (**Figure** **4**A), and we modified the connectome matrix to incorporate the effect of gene expression on outgoing connections (Figure 4B), incoming connections (Figure 4C), or both (combined effect; Figure 4D) (see Equations (9) and (10) in the Experimental Section).

Based on our WT mice data, individual genes were incorporated in the model, and ranked according to the model performance (Figure 4E–J; also see Table S2 in the Supporting Information). We compared the results of these “connectome+gene” models with the global spread model (Figure 2A) or gene correlations (Figure S12), showing that our “connectome+gene” models predicted the pathological α‐Syn spreading better than the other two models (Figure S13, Supporting Information). Then, genes that improved the Ave. CCC values over the global spread model were considered to contribute to the selective vulnerability of pathological α‐Syn spreading. We identified *Dok5* (Ave. CCC = 0.722), *Grid2ip* (Ave. CCC = 0.696), and *Pde1a* (Ave. CCC = 0.701) as the single‐best genes for the outgoing, incoming, and combined effect, respectively (Figure 4E–G). We also showed the distribution of Ave. CCC values for all genes (Figure 4K–M). Overall, distinct genes were identified for outgoing, incoming, and combined effects, which suggests that different components of spreading process may be modulated by distinct sets of genes. The top incoming gene, *Grid2ip*, did not contribute to selective vulnerability in the outgoing (ranking 3736th) and combined (ranking 3791st) effects. By contrast, *Dok5* and *Pde1a* performed very well for outgoing effect (*Dok5*, ranking 1st; *Pde1a*, ranking 53rd), incoming effect (*Dok5*, ranking 53rd; *Pde1a*, ranking 58th), and combined effect (*Dok5*, ranking 2nd; *Pde1a*, ranking 1st) (see Table S2 in the Supporting Information). These results suggest that *Dok5* and *Pde1a* influence both the presynaptic and postsynaptic regions to modulate the spread, whereas *Grid2ip* only influences the postsynaptic regions. Furthermore, similar to the global spread model, the gene model also captured the scale features of the data (Figure S14, Supporting Information)^[^ ^24^ ^]^ and the hemispherical asymmetry of the pathological distribution (Figure S15, Supporting Information). We then visualized the pathological distribution heatmaps predicted by these genes (Figure S16, Supporting Information). Interestingly, the outgoing and combined effect altered the Ave. CCC values much more than the incoming effect (Figure 4K–M). The incorporation of the best genes into the model only increased the Ave. CCC values by less than 0.1 (Figure 4E–G), suggesting that the connectome played a predominant role in predicting the spread of pathological α‐Syn.

We also investigated the effects of four important PD genetic risk factors (*Snca*, Figure 4H–J; *Gba*, Figure S17, Supporting Information; *Vps35*, Figure S18, Supporting Information; *Gpnmb*, Figure S19, Supporting Information). *Snca* modulated both the outgoing (ranking 353rd; Figure 4H,K) and incoming effects (ranking 356th; Figure 4I,L), supporting that the expression of *Snca* affected not only the presynaptic but also the postsynaptic sites. Also, *Snca* was ranked higher for all three effects than *Gba* and *Vps35* (Figure 4K–M), demonstrating the greater importance of *Snca* in modulating the spread of pathological α‐Syn over *Gba* and *Vps35*. We instead found that *Gba* and *Vps35* mainly modulated the outgoing effect (*Gba*, ranking 497th, Figure S17A (Supporting Information) and Figure 4K; *Vps35*, ranking 506th, Figure S18A (Supporting Information) and Figure 4K) rather than the incoming effect (*Gba*, ranking 3181st, Figure S17B (Supporting Information) and Figure 4L; *Vps35*, ranking 2370th, Figure S18B (Supporting Information) and Figure 4L), indicating that the *Gba* and *Vps35* genes mainly encoded the selective vulnerability of presynaptic site by altering the regional outgoing connections. Additionally, *Gpnmb* has been reported to participate in the cellular uptake of pathological α‐Syn.^[^ ^28^ ^]^ We found that, compared to the global spread model (Figure 2A), *Gpnmb* improved the model performance (Figure S19, Supporting Information) for its outgoing (Ave. CCC = 0.637), incoming (Ave. CCC = 0.623), and combined effects (Ave. CCC = 0.635), validating its importance in the spread of pathology. Unlike previous studies,^[^ ^7^, ^17^, ^19^ ^]^ our study provided critical insights into how genetic risk factors affected spreading by identifying effect regions that were presynaptic, postsynaptic, or both.

**Figure 5:**
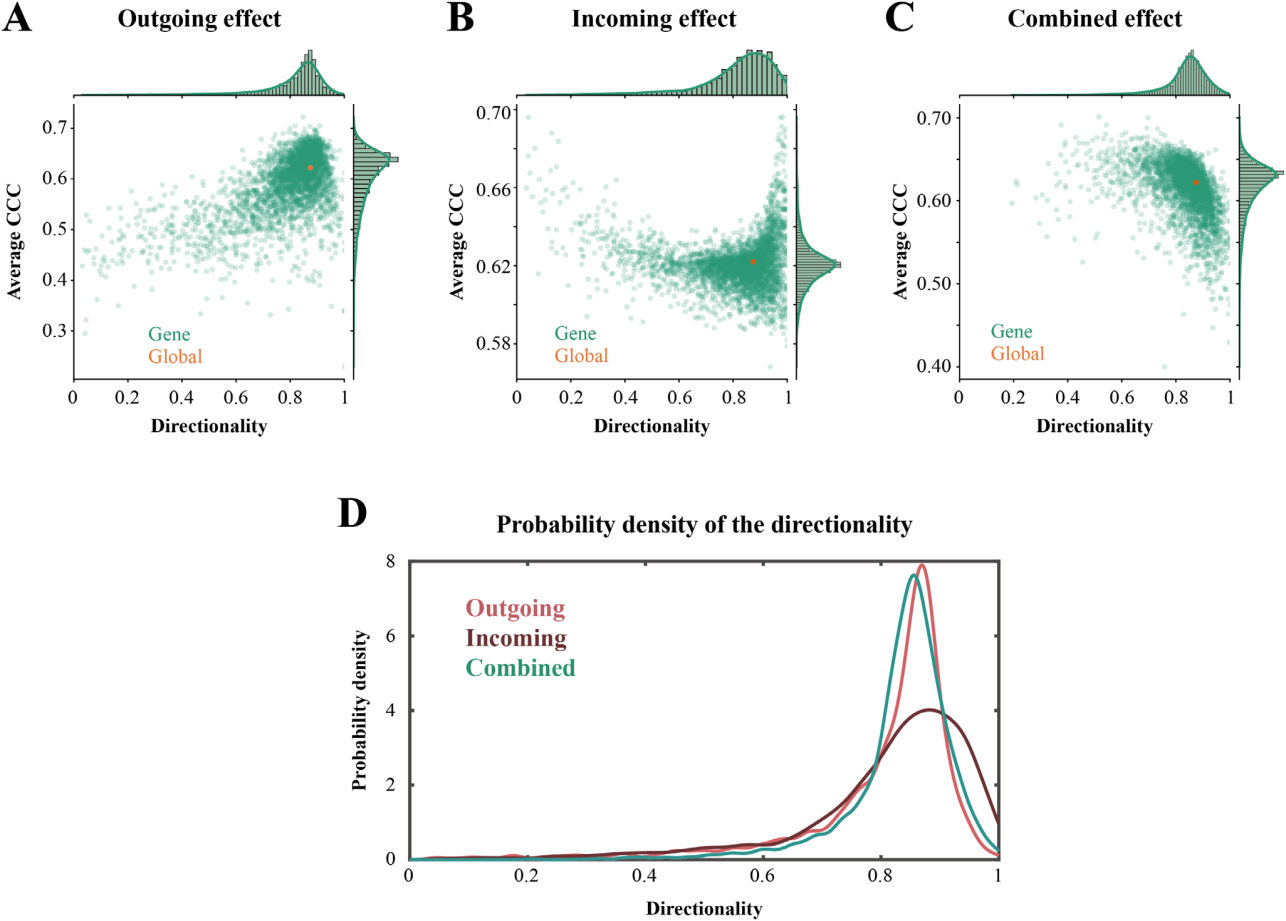
The relationship between individual gene expression and the spread directionality of the gene model. A–C) Directionality versus Ave. CCC, where each green dot represents one gene and the orange dot represents the best results for the global spread model. The *x*‐axis and *y*‐axis represent their directionality parameter values and Ave. CCC, respectively. D) Probability density of the directionality of the three groups, where the *x*‐axis and *y*‐axis represent the directionality parameter values and the probability density, respectively.

Further, we sought to investigate whether gene expression altered the spread directionality preference. The probability density of the directionality showed that the spread was still predominantly retrograde after considering the gene effect under all three conditions (**Figure** **5**), confirming our hypothesis that the spread of pathological α‐Syn in WT mice was predominantly retrograde.

**Figure 6:**
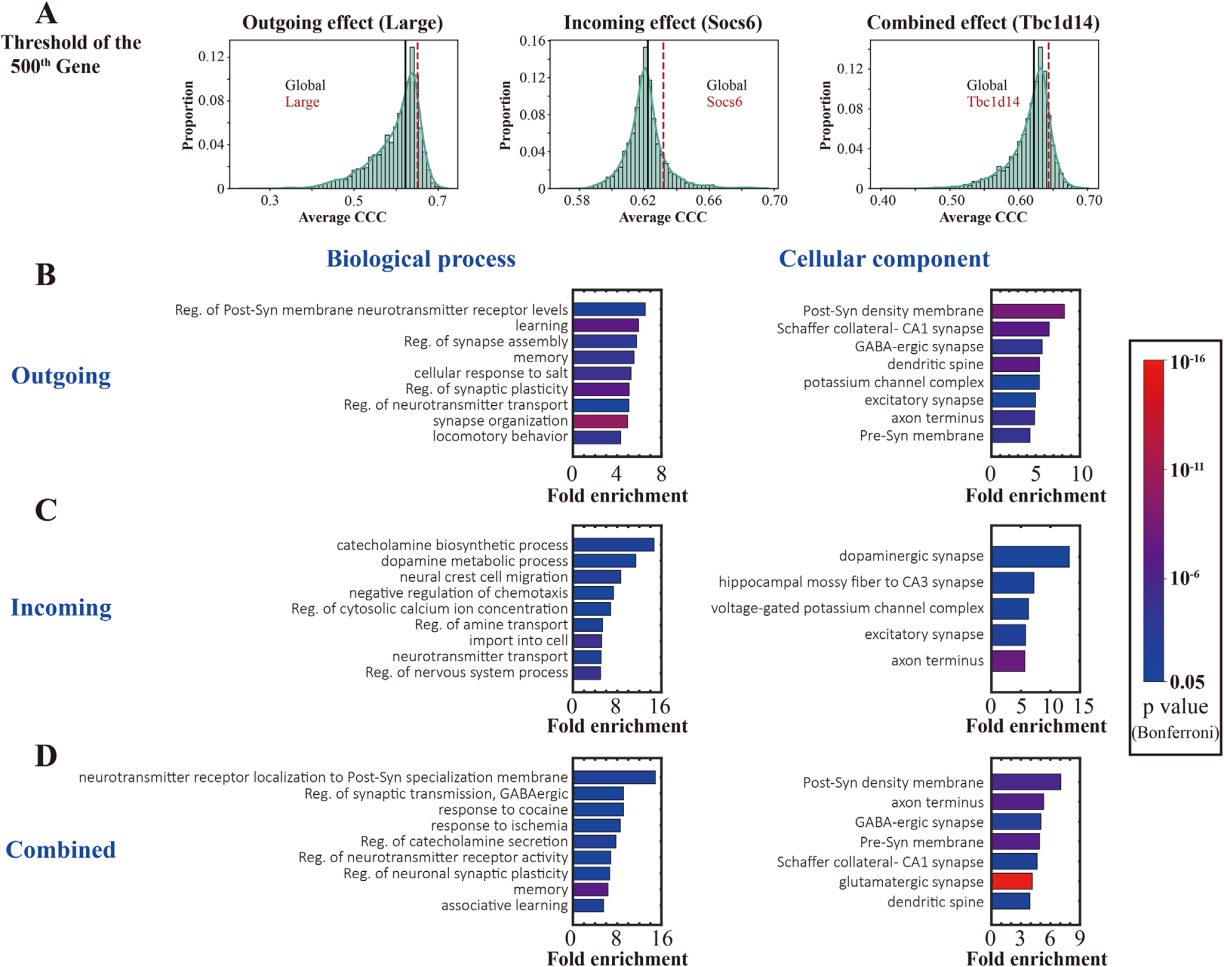
GO analyses for the top 500 genes of outgoing, incoming, and combined effect. A) Threshold of the 500th gene of the three groups (*Large* for the outgoing effect, *Socs6* for the incoming effect, and *Tbc1d14* for the combined effect). Each of the three groups exhibited better performance than the global spread model (*p* < 0.0001, one sample Wilcoxon test against the Ave. CCC value of global spread model). B–D) Biological process and cellular component of GO analyses for the candidate genes from (B) outgoing effect; (C) incoming effect; (D) combined effect. For each GO entry, the displayed results were strictly sorted by the highest fold enrichment and statistically significant after Bonferroni correction. The colors of the bars represent the *p* values, and the length represents the fold enrichment. Only the primary hierarchy is shown. Abbreviations: Reg., regulation; Post‐Syn, postsynaptic; Pre‐Syn, presynaptic.

To study the similarities and differences between the subsets of genes contributing to selective vulnerability of outgoing, incoming, and combined effects, we selected the top 500 genes of the three conditions as the candidate risk genes for further investigation (**Figure** **6**A). In order to ensure that these genes all had robust performance in predicting the spread of pathological α‐Syn, we conducted bootstrap analyses for each group's 500th gene (500th‐ranked‐gene for the outgoing, incoming, and combined effects were *Large*, *Socs6*, and *Tbc1d14*, respectively) by randomly permuting the elements of the gene expression vectors for 1000 times. As expected, the performance of the 500th gene's actual expression values exceeded the 95% percentiles of the permutations for each group (Figure S20, Supporting Information), demonstrating that these genes significantly improve model performance relative to a “null” set of genes. We assessed the overlapping of the top 500 genes for the three conditions (Figure S21, Supporting Information), which showed a highest overlap between outgoing and combined effects. We also found those genes that strongly modulated the outgoing and incoming effects also modulated the combined effects (Figure S21, Supporting Information).

We then carried out gene ontology (GO) analyses^[^ ^29^ ^]^ for the top 500 candidate genes (Figure 6B–D and Figure S22 (Supporting Information)). Interestingly, the candidate genes were all enriched in synaptic components and ion channels such as the dopaminergic (Figure 6C), glutamatergic (Figure 6B–D), and GABAergic synapses (Figure 6B,D), and voltage‐gated ion channels (Figure S22, Supporting Information). These findings were consistent with the observation that neuronal firing patterns can affect the spread of pathological α‐Syn.^[^ ^30^, ^31^, ^32^ ^]^ Outgoing genes were enriched in biological processes including learning, memory, and locomotory behavior (Figure 6B). By contrast, incoming genes were mainly related to the dopaminergic system and synapses (Figure 6C). We therefore find that while there is significant overlap in the functional enrichment of these gene subsets, there are also important differences that suggest that gene modulation of outgoing versus incoming genes may involve different biological and molecular processes.

### 2.5 Distinct Cell Type Enrichment for Outgoing, Incoming, and Combined Genes

**Figure 7:**
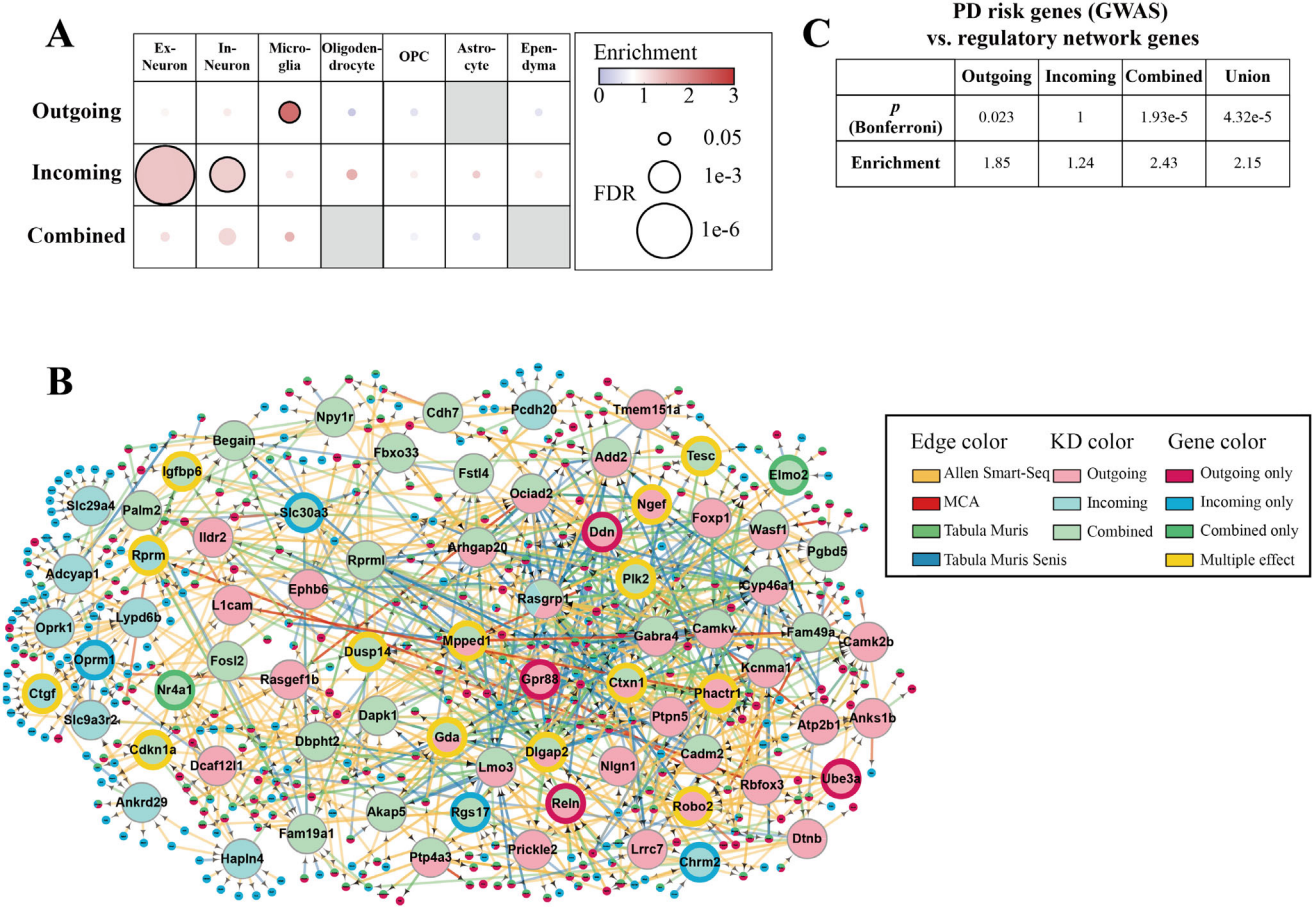
Results of cell‐type, key driver (KD), and GWAS‐associated enrichment analyses for the candidate (top 500) genes of outgoing, incoming, and combined effects. A) Cell‐type analyses based on the enrichment of cell‐type marker genes among the candidate genes of each effect. The sizes of the circles indicate FDR values within each row and the face colors indicate enrichment. Circles with black boundaries were statistically significant after FDR correction (*α* = 0.05). The gray cells indicate missing results. Abbreviations: Ex – excitatory, In – inhibitory, OPC – oligodendrocyte progenitor cell. B) The union neuronal SCING gene regulatory network of three individual regulatory networks of the top KDs for the candidate genes with outgoing, incoming, and combined effects. Gene regulatory networks were constructed using SCING and neuronal scRNAseq data from different single cell Atlases including the Allen Brain Single Cell Atlas, the Mouse Cell Atlas (MCA), Tabula Muris, and Tabula Muris Senis (see the Experimental Section). SCING networks were constructed from each dataset and the union network was used for key driver analysis. The larger, labeled circles indicate genes that were identified to be KDs, and smaller circles indicate candidate genes with the three effects. The direction of edges between genes indicates the regulatory relationship within SCING networks. For KDs, the color inside of each circle indicates from which candidate gene set the KD was identified, and multiple colors indicate the KD gene was a KD for multiple categories of candidate genes. The boundary color indicates whether the KD gene itself appeared as a candidate gene in any effect (or multiple effects). C) Enrichment analyses between the GWAS PD risk genes and our regulatory network genes. The outgoing, incoming, combined, and union represent the genes from different SCING networks (Figures S24–S26 (Supporting Information) and (B)). The *p* values were Bonferroni corrected with *n* = 4.

It is unclear which cell types contribute most to the selective vulnerability of pathological α‐Syn spreading. To address this question, cell‐type enrichment analyses^[^ ^33^ ^]^ were performed on the top 500 genes for each of the three different effect conditions (**Figure** **7**A and Table S3 (Supporting Information)), which revealed distinct cell‐type enrichment preferences for the different effects. Outgoing genes were significantly enriched in microglia cells, while incoming genes were significantly enriched in neurons (including excitatory and inhibitory neurons). This result suggests that microglial cells at presynaptic sites and neurons at postsynaptic sites show stronger effects in modulating pathological α‐Syn spread.

### 2.6 Key Driver Analyses and GWAS Enrichment Analyses Help identify and validate Key Driver Genes

To further compare these candidate genes subsets, cell‐type specific gene regulatory networks were constructed using SCING.^[^ ^34^ ^]^ Key driver (KD) analyses^[^ ^35^ ^]^ were then performed to identify the network key drivers of the selective vulnerability related genes based on the network topology (see the Experimental Section), where key drivers are the network hub genes whose neighborhoods are overrepresented by the vulnerability candidate genes. We first constructed the gene regulatory network for outgoing effect candidate genes and investigated their relationship based on the glial cell single‐cell RNA sequencing data (Figure S23 and Table S4, Supporting Information), since outgoing effect candidate genes were enriched in glial cells (Figure 7A). In order to deeper understand how three candidate gene subsets work in the neuronal cell type, we built regulatory networks based on neuronal networks (see the Experimental Section) for outgoing (Figure S24, Supporting Information), incoming (Figure S25, Supporting Information), and combined effects (Figure S26, Supporting Information), respectively (also see Table S5 in the Supporting Information). We merged these three individual networks (Figures S24–S26, Supporting Information) together to visualize the union gene regulatory network (Figure 7B), which contained the top KD genes for all three effects.

Interestingly, KD genes within the same effect were closer to each other in the network (Figure 7B), highlighting the differences between the three effects. The connections of outgoing and combined KD genes were denser than that of the incoming KD genes (Figure 7B), demonstrating the more distant regulatory relationship of genes with incoming effect. To quantify this topological structure, we performed chi‐square goodness of fit tests for KDs of each effect (Figure S27, Supporting Information). Our results showed that KD genes had closer relationship to genes from the same group than to genes from the other groups, and the KDs for the incoming genes had significantly fewer neighbor genes in the union network than the KDs for the outgoing and combined genes.

Finally, we analyzed the enrichment of candidate genes for PD risk genes from GWAS Catalog,^[^ ^36^ ^]^ which showed that PD risk genes were significantly enriched in the outgoing regulatory network, the combined regulatory network, and the union of three regulatory networks (Figure 7C and Table S6 (Supporting Information)). Overall, our gene regulatory network KD analyses further identified potential regulators and interactions among genes associated with selective vulnerability, pathological α‐Syn spreading, and PD risk factors (Figure 7C).

### 2.7 Functional Validation of Candidate Gene *RORA* in Mouse Primary Neurons

Although the reliability of our candidate gene list is supported by PD GWAS enrichment analysis, an important question remains as to whether these genes possess true biological functions related to pathological α‐Syn spreading. Some genes from our candidate list, such as *Gba* and *Gpnmb*, have already been functionally validated in previous studies,^[^ ^19^, ^28^ ^]^ demonstrating their association with pathological α‐Syn spreading. However, the functional roles of many other genes on our gene list remain unexplored.

**Figure 8:**
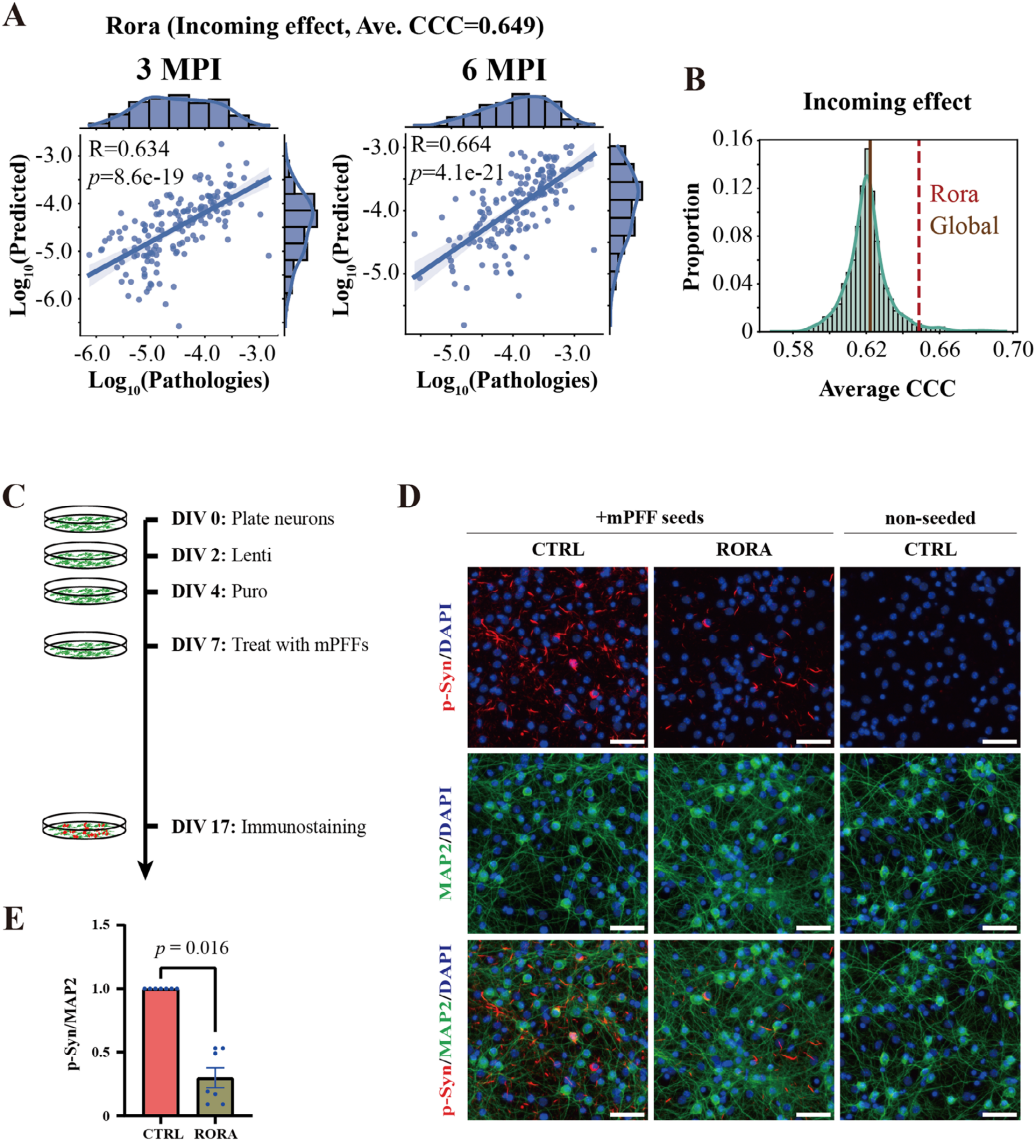
*RORA* overexpression reduces α‐Syn inclusions in mouse primary neurons. A) Model results of *RORA* gene for its incoming effect (ranking 120th). Each dot represents one brain region, while the *x*‐axis and *y*‐axis represent the pathology (log10‐transformed) found empirically and predicted by the model, respectively. The Pearson's correlation coefficient and the best regression lines for both 3 and 6 MPI are also displayed. The shaded ribbon represents the 95% prediction interval. Abbreviations: *R*, Pearson's correlation coefficient; *p*, *p* values from linear regression. B) The distribution of model results of incoming effect and the ranking position of *RORA*. C) Schematic representation of the experimental timeline for the primary mouse cortical neuron assay of synuclein inclusion formation. Embryonic cortical neurons were plated on day in vitro 0 (DIV0), followed by the addition of homemade lentivirus at DIV2. Puromycin selection began two days later (DIV4), and recombinant mouse PFFs were introduced at DIV7. At DIV17, cultures were detergent‐extracted and fixed before immunostaining with an anti‐α‐Syn Phospho (Ser129) antibody (81A) to detect synuclein pathology, MAP2 as a neuronal health marker, and DAPI to visualize cell nuclei. Plates were imaged, and quantification was performed for total integrated density of the 81A signal, total MAP2‐positive area, and DAPI‐positive cell counts/area. D) Representative images showing α‐Syn pathology with a significant reduction upon *RORA* overexpression. Cultures were also stained with DAPI to visualize cell nuclei and MAP2 to assess neuronal morphology, which were unaffected by mpffs treatment or *RORA* overexpression. Scale bar = 50 µm. E) Quantification of relative α‐Syn inclusions in control and *RORA*‐overexpressing groups, with synuclein pathology (IntDen sum) normalized to MAP2 signal (area). Statistical analysis was performed using one sample Wilcoxon test. Each dot represents an independent experiment (*n* = 7), and error bars indicate standard error of the mean.

To further address this, we selected *RORA* for additional functional validation (see the Experimental Section). *RORA* has been reported to exert neuroprotective effects in Parkinson's disease;^[^ ^37^ ^]^ however, its role in modulating pathological α‐Syn spreading remains unclear. According to our mathematical model, *RORA* ranked highly (120th) in terms of its incoming effect (**Figure** **8**A,B), suggesting a potential association with pathological α‐Syn spreading. Our in vitro experiments further supported this hypothesis, showing that after PFF treatment, α‐Syn pathology was reduced in *RORA*‐overexpressing mouse neurons compared to the control group (Figure 8C–E). Therefore, the protective effect of *RORA* in PD may also extend to its inhibitory influence on pathological α‐Syn transmission. In summary, the functional validation of *RORA* highlights the novelty and effectiveness of our mathematical model and methodology presented in this study.

## 3 Discussion

In this study, we provided a mathematical modeling approach and combined it with systems biology analysis tools to discover potential risk genes for the spread of pathological α‐Syn in PD. We investigated the nuanced directionality preference of pathological α‐Syn spread, and successfully predicted its spreading along neuronal connectivity network in the central nervous system. Compared to previous studies,^[^ ^7^, ^17^, ^18^, ^19^, ^20^ ^]^ the current work considered the directionality preference of pathological α‐Syn spreading as a parameter, allowing us to model directionally biased spread accurately. Our results suggest that the spread of pathological α‐Syn in WT mice is predominantly retrograde but also has a small component of anterograde direction (Figure 2 and Figure S7A (Supporting Information)). We also investigated the outgoing, incoming, and combined effects of gene expression on spreading directionality, which improved the model fitting performance over the global spread model and gene correlation models (Figure S13, Supporting Information) and again showed a similar predominantly retrograde direction as in the global spread model (Figure 5). Our mathematical modeling therefore demonstrated that while α‐Syn spread is predominantly retrograde, incorporating a minor anterograde component is important to improve the accuracy of data prediction in WT mice. More interestingly, we found that the spread directionality of pathological α‐Syn was very different in *G2019S LRRK2* mice (Figure S7, Supporting Information), which is the first report on how *G2019S LRRK2* affects the spread directionality preference. Overall, our current study has quantified the proportion of pathological α‐Syn spreading for the first time, which gives a more nuanced picture of the directional preference of α‐Syn than previously assumed.^[^ ^7^, ^17^, ^18^, ^20^ ^]^

Although previous studies showed that the spread of pathological α‐Syn followed the mesoscale connectome,^[^ ^17^, ^18^ ^]^ it is not clear whether all connections contribute equally to pathological α‐Syn spreading.^[^ ^14^, ^15^ ^]^ We used bootstrapping method to demonstrate that pathological α‐Syn propagated through the anatomic connections, and strikingly, we found that the spread of pathological α‐Syn was primarily driven by the partial connectome composed of just the top 2% strongest connections (Figure 3). We therefore propose the existence of the key subnetworks that drive the pathological spread for the first time (Figure 3G,H and Figure S10 and Table S14 (Supporting Information)), which allows us to localize the key brain regions and connections. Interestingly, most of the key regions implicated in PD Braak staging^[^ ^38^ ^]^ are represented within the strongest 2% of connections (Table S14, Supporting Information). These include the olfactory bulb (Braak stage 1), piriform area, entorhinal area, substantia nigra (Braak stage 3), midbrain (Braak stages 3 and 4), caudoputamen, amygdala (Braak stages 4–6), hippocampus (Braak stages 4–6), and somatosensory area (Braak stages 5 and 6). More specifically, certain connections appear to reflect the progression of pathology. For example, we observed strong connections between the olfactory bulb and piriform area, as well as between the piriform area and amygdala, which may provide insights into how pathology propagates across these regions. The existence of the key connectome has also been validated by the external datasets from Henderson et al.,^[^ ^7^ ^]^ which demonstrated that a small subset of the strongest connections within the connectome could predict the pathological α‐Syn spreading both in WT mice and *G2019S LRRK2* mice (Figure S9, Supporting Information). Additionally, we note that the key connectomes of pathological α‐Syn progression represent one mechanism, but not the only one. While the strongest connections can partially explain the progression of pathological α‐Syn in PD patients, this progression is also influenced by a variety of other factors, including genetic and environmental influences. Nevertheless, the findings of key connectomes enable us to assess the contribution of each of the strongest connections to pathological α‐Syn spreading that could be explored with future experiments.

In addition to the connectome, we studied distinct effects of regional gene expression on pathological α‐Syn spread, including the outgoing, incoming, and combined effects for the first time (Figure 4; see the Experimental Section). Previously, only the outgoing effect has been modeled in mathematical models.^[^ ^7^, ^13^, ^17^ ^]^ We investigated the case of four important PD risk genes, i.e., *Snca*,^[^ ^7^ ^]^ *Gba*,^[^ ^19^ ^]^ *Vps35*,^[^ ^17^ ^]^ and *Gpnmb*.^[^ ^28^ ^]^ Our study indicated that *Snca* and *Gpnmb* affected not only the presynaptic but also the postsynaptic regions, while *Gba* and *Vps35* mainly modulated the presynaptic sites (Figure 4H–M and Figures S17–S19 (Supporting Information)). Interestingly, none of these four genes are the top candidate genes that contribute to the selective vulnerability. These findings suggest that the genes modulating pathological α‐Syn spreading may be distinct from those genes that are thought to be PD risk factors. In addition, it will be more cost‐effective to use these theoretical models predicting the outgoing, incoming, or combined effects for individual gene before downstream functional studies, since each functional study may only focus on one partial step of the spreading. Overall, our study provides a new platform for studying the trans‐synaptic mechanisms of spread, especially the effects of the expression of specific genes on whether the source of the selective vulnerability emanates from the presynaptic or postsynaptic sites.

The Venn diagram (Figure S21, Supporting Information) showed a higher gene overlap ratio between genes with the outgoing effect and the combined effect than between pairs of gene sets with the other effects. Since the combined effect modeled the case that gene expression affected the presynaptic and postsynaptic regions equally, this result indicates that selective vulnerability is mainly driven by genes expressed at presynaptic rather than postsynaptic sites. It is also reflected in the fact that the outgoing and combined effects altered the Ave. CCC values much more than the incoming effect (Figure 4K,L). Given that the spread of pathological α‐Syn is predominantly retrograde in WT mice (Figures 2 and 5), these results suggest that genes expressed at sites that receiving pathology rather than those that sending pathology predominantly modulates pathological α‐Syn spread.

Our model allows us to rank the importance of genes by their model performance, showing a promising approach to explore PD risk genes from the perspective of pathological spreading (Figure 4K–M). Based on published literature (**Table** **1**), we found that the top‐ranked risk genes in terms of combined effect (Figure 4M and Table S2 (Supporting Information)) not only exhibited strong model performance but were also linked to the progression of pathological α‐Syn, synucleinopathies, or other neurodegenerative diseases. Apart from these, genes from other effects may also be related to PD, such as the *Spp1* (149th for incoming effect)^[^ ^39^ ^]^ and *Dok5* (1st for outgoing effect, and 2nd for combined effect, Table 1). Specifically, we found that the *RORA* gene exhibited a strong incoming effect (Figure 8A,B). We then conducted functional validation, biologically confirming its inhibitory role in pathological α‐Syn spreading in mouse primary neurons (Figure 8C–E). Therefore, our approach provides a novel pipeline for computationally identifying and biologically validating risk genes associated with pathological spreading.

**Table 1:** Relationship between top combined genes obtained from our model and evidence from previous studies.

Gene name	Ranking	Related evidence	Refs.
*Pde1a*	1st	α‐Syn toxicity	[40, 41]
*Dok5*	2nd	Member of *Dok* gene family	[42, 43]
*Mpped1a*	3rd	Important in CA1 neurons	[44, 45]
*Bmp3*	4th	Member of *Bmp* gene family	[46]
*Ctgf*	5th	Dopaminergic neurodegeneration	[47, 48]
*Slc2a3*, *Slc2a13*	6th, 7th	Member of *Slc2* gene family	[49, 50]

The results of the GO analysis further summarized risk genes between pathological α‐Syn spread and other related studies of synucleinopathies, and may explain the genetic comorbidity mechanisms of PD and other neuropsychiatric disorders or neurological diseases. Besides learning, memory, and locomotory behavior (Figure 6B,D), the candidate genes of our model were also related to biosynthetic and metabolic process of dopamine and catecholamine (Figure 6C,D), which is in line with the known selective vulnerability of dopaminergic neurons in PD.^[^ ^51^ ^]^ Since the dopamine circuit is associated with the reward and decision‐making system,^[^ ^52^ ^]^ this result may reflect the comorbidity between mood disorder and PD.^[^ ^53^, ^54^ ^]^ The genes were also related to “response to cocaine” and “response to ischemia” (Figure 6D). The relationship between cocaine and PD are well documented,^[^ ^55^, ^56^, ^57^ ^]^ particularly in the context of cocaine abuse^[^ ^57^, ^58^ ^]^ and the neurotoxicity^[^ ^59^ ^]^ leading to PD. Accordingly, since cocaine acts in dopaminergic neurons of ventral tegmental area and other brain regions,^[^ ^60^ ^]^ they may share similar neural circuits as PD,^[^ ^61^ ^]^ and these neural circuits may be vulnerable to pathological α‐Syn spread. Similarly, PD‐related proteins can mediate secondary brain damage after cerebral ischemia,^[^ ^62^ ^]^ and comorbidity between stroke and PD has also been reported;^[^ ^63^ ^]^ our results therefore indicate a spreading‐related mechanism underpinning the potential relationship between ischemia and PD as well. In summary, our results point to a promising bridge between PD, other neurological diseases, neuropsychiatric disorders, drug abuse, ion channels, and pathological α‐Syn spread, and provide an insight into the risk genes involved in the mechanisms underlying their comorbidity.

Cell type enrichment analyses further suggest that distinct cell types play different roles in modulating pathological α‐Syn spread, with microglial cells and neurons playing outsize roles at presynaptic sites and postsynaptic sites, respectively (Figure 7B). However, there was no significant cell‐type preference in the combined effect (Figure 7B); given that the combined effect blurs the contributions of outgoing and incoming effects, this result emphasizes that studying the impact on outgoing and incoming connections separately is required to disentangle the cell types involved in mediating each process.

To assess the interactions among the candidate genes involved in α‐Syn spreading, we built gene regulatory networks, and the key driver analyses obtained KD genes which were potential regulators of the candidate genes. These KD genes may play important roles in regulating PD or synucleinopathies. Among the KD genes whose network neighborhoods showed top fold enrichment rankings for candidate genes with multiple effects are *Mpped1* and *Ctgf* discussed above, as well as *Dlgap2*, whose network neighborhood has the highest fold enrichment for genes with outgoing and combined effects and is associated with age‐related cognitive decline (Figures S24–S26 and Table S5, Supporting Information).^[^ ^64^ ^]^ By identifying the relationships between KD and candidate genes, it also allows us to elucidate the regulatory networks involved in mediating the spreading of α‐Syn pathology. As expected, the GWAS enrichment analyses showed that the combined and union regulatory networks had higher enrichment for PD risk genes (Figure 7C), demonstrating that the mechanisms by which these genes contribute to the risk of developing PD include the mediation of network spreading of α‐Syn.

Another significant outcome of the current study is that our proposed methodology combining mathematical modeling (Figures 2, 3, 4, 5), system biology analyses (Figures 6 and 7), and biological validation (Figure 8) can be used to facilitate translational research from animals to humans. Future work includes applying our mathematical models to PD patient imaging data, which may become a promising tool for diagnosis and disease treatment at early stage. Although there have been promising recent developments toward direct, in vivo imaging of α‐Syn using positron emission tomography,^[^ ^65^ ^]^ no publicly available datasets are currently available for analyzing the spatiotemporal deposition of α‐Syn in human subjects. Nevertheless, previous mathematical modeling by our group and others has been employed to explain the progression of neurodegeneration as assessed by deformation‐based morphometry in PD patients,^[^ ^20^, ^66^ ^]^ and also explained the patterns of neurodegeneration of AD and other tauopathies,^[^ ^25^, ^67^, ^68^ ^]^ as well as amyotrophic lateral sclerosis.^[^ ^69^ ^]^ Recently, synaptic oligomeric tau was directly observed in the brains of AD patients,^[^ ^70^ ^]^ providing further motivation for using mathematical models in prion‐like diseases. Combined with the regional gene expression data in humans provided by the AIBS,^[^ ^71^ ^]^ our model will provide a critical tool to analyze the mechanisms of gene‐mediated pathological protein spread throughout the brain in other neurodegenerative diseases.

We note that the current study has several limitations, which we plan to further investigate experimentally in the future. While the AGEA^[^ ^16^ ^]^ remains the most expansive resource for spatial gene expression data in the mouse brain, we recognize that gene expression levels may be altered with the disease progression after PFF injection, which the AGEA does not capture. Consequently, we can only evaluate the effect of baseline gene expression on pathology spread, as many previous studies have done.^[^ ^7^, ^13^, ^17^, ^19^, ^72^ ^]^ Additionally, our study was limited by the current resolution of AMBA and the mesoscale connectome data^[^ ^14^, ^15^ ^]^ consisting of 410 brain regions; this may have led us to overlook pathology spread between potentially important subregions as well as local neural networks. We therefore plan to perform in vitro experiments to investigate spread processes at finer spatial scales. Besides, our model does not incorporate the effects of degeneration, since we only studied the progression at early stages of the disease, and previous studies have demonstrated there are few brain regions that show significant cellular degeneration before 6 MPI.^[^ ^7^, ^73^ ^]^ However, degeneration may play a more significant role at later stages of disease, therefore we will conduct more experiments to assess α‐Syn pathology beyond 6 MPI and update our model accordingly. Finally, the functions of the risk genes predicted by our model have not been fully validated biologically, and we will conduct more experiments to validate them in the future.

## 4 Experimental Section

### Animals

Two‐to‐three‐month‐old C57BL6/C3H wild‐type mice were purchased from the Jackson Laboratories (Bar Harbor) for the stereotaxic injection experiments, which has been reported in our previous study.^[^ ^1^ ^]^ Mouse cortical neurons were prepared in‐house from E16.5 embryos of CD1 mice (Charles River) for functional validation of *RORA*. All breeding, housing, and experimental procedures were performed according to the NIH Guide for the Care and Use of Experimental Animals and approved by the UCLA Institutional Animal Care and Use Committee (NO: ARC‐2020‐018).

### In Vitro α‐Syn Preformed Fibril Generation

Full‐length mouse α‐Syn (1‐140) monomers were expressed in BL21 (DE3) RIL cells and purified referring to the previous protocols.^[^ ^74^ ^]^ Mouse PFFs were generated by diluting the above α‐Syn monomers to 5 mg mL^−1^ in sterile Dulbecco's phosphate‐buffered saline (PBS) (Cellgro, Mediatech; pH adjusted to 7.0), and then incubating it at 37 °C with constant agitation at 1000 r.p.m for 7 days. The sedimentation test and thioflavin T‐binding assay were used to verify α‐Syn fibrillization.^[^ ^75^ ^]^

### Stereotaxic Injection of Mouse α‐Syn PFFs

Two‐to‐three‐month‐old C57BL6/C3H wild‐type mice were anesthetized with ketamine hydrochloride (100 mg kg^−1^), xylazine (10 mg kg^−1^), and acepromazine (0.1 mg kg^−1^). The amount of 6.25 µg mouse PFF in 2.5 µL Dulbecco's PBS was stereotaxically injected into the dorsal striatum (caudoputamen; coordinates: 0.2 mm relative to bregma, +2.0 mm from midline, +3.2 mm beneath the surface of skull) with 10 µL syringes (Hamilton) at a rate of 0.4 µL min^−1^. Mice were euthanized at 3 and 6 months‐post‐injection.

### Immunohistochemistry

Mice were transcardially perfused with PBS. The brain and spinal cord were removed and fixed in 70% ethanol (in 150 mm NaCl, pH 7.4) overnight. After fixation, brains were embedded in paraffin blocks and the immunohistochemistry was performed as previously described.^[^ ^76^, ^77^ ^]^ Phosphorylated at Ser 129 (pS129, 81A, 1:10 000 dilution) antibodies were used for misfolded α‐Syn proteins. Staining sections were scanned by a Perkin Elmer Lamina scanner at 20 × magnification. Sample size: *n* = 4 for the three months‐post‐injection mice, *n* = 3 for the six months‐post‐injection mice.

### Quantitative Pathology

All section selection, annotation, and quantification were performed by mouse neuroanatomists who were blinded to the treatment. For the quantification of the α‐Syn pathology, coronal slices were selected to closely match the following nine bregma levels: +4.28, +2.10, +0.98, −0.22, −1.22, −2.18, −2.92, −3.52, and −4.48 mm. For each bregma level of an individual mouse, 2 slices were selected as the technical replicates. The digitized images were uploaded into the QuPath software to allow annotation and quantification of the percentage area occupied by α‐Syn pathology. Standardized annotations were drawn to allow independent quantification of 196 gray matter regions throughout the brain. Each set of annotations was hand‐drawn onto the desired sections to match the designated brain regions. After annotations of all brains, analysis algorithms were applied to all stained sections, and data analysis measures for each region were recorded.

One analysis algorithm was applied to the tissue, but it varied depending on the level of background staining observed in the mice. The algorithm detected a total signal above a minimum threshold, which was determined to most likely not include any background signal. Specifically, the analysis included all signal that was above a 0.5 or 0.7 optical density threshold, based on the analysis algorithm used. Additionally, the analysis tool was developed with a resolution of 0.49 micrometers per pixel and a smoothing sigma of 0.5. The signal was then normalized to total tissue area.

Finally, in order to match our quantitative data to the connectome data for subsequent modeling work, we merged and added some brain regions. We eventually obtained the pathological dataset containing 410 grey matter regions, with 205 regions bi‐hemispheric symmetry. Among these, data of 156 brain regions were derived from the quantification of pathological immunohistochemistry. The value of each brain region represented the regional pathological burden (the area of pathology over the area of the whole region). Since the amounts of pathology between different regions could vary by a factor of 1–1000, we took the logarithm transformation (log10‐transformed) for visualizing the quantitative data at 3 and 6 MPI averaged within its group.

### Plasmid Construction

The lentivirus overexpressing *RORA* (pLX317‐RORA‐v5‐puro) was obtained from the Molecular Screening Shared Resource core facility at UCLA. The control plasmid, pLX317‐v5‐puro, was derived from pLX317‐EGFP‐puro (Addgene, Plasmid #115439) by polymerase‐chain‐reaction (PCR)‐mediated deletion of a major portion of EGFP using Q5 Hot Start High‐Fidelity DNA Polymerase (New England, Cat. #E0554S). The following primers were used for PCR: forward primer: gccgacccagctttcttgtac, reverse primer: gcccttgctcaccatggtgaag. After PCR, the Kinase‐Ligase‐DpnI (KLD) enzyme mix was used for circularization and template removal. The KLD‐treated product was then transformed into competent cells, and a single clone was selected and subjected to miniprep (Invitrogen, Cat. #K210010). The plasmid sequence was verified by whole‐plasmid sequencing performed by Plasmidsaurus.

### Generation of Control and *RORA*‐Expressing Lentivirus

Control lentivirus and lentivirus for exogenous *RORA* expression were generated as follows. HEK293T cells were thawed, passaged, and maintained in T25 flasks in 3+ Dulbecco's modified Eagle medium (DMEM (Gibco, Cat. #11960077) supplemented with 10% fetal bovine serum (FBS) (Gibco, Cat. #A5670801), 1% GlutaMAX (Gibco, Cat. #35050079), and 1% penicillin/streptomycin (Corning, Cat. #30002CI)). Cells were plated in 6‐well plates at a density expected to reach 60–70% confluency the following day.

On the day of transfection, 2 µg of the expression plasmid and 3 µg of third‐generation packaging plasmids—pRSV‐Rev (Addgene, Plasmid #12253), pMDLg/pRRE (Addgene, Plasmid #12251), and pMD2.G (Addgene, Plasmid #12259)—were diluted in Opti‐MEM I Reduced Serum Medium (Gibco, Cat. #31985070) and thoroughly mixed. Lipofectamine 2000 (Invitrogen, Cat. #11668027) was separately diluted in Opti‐MEM and incubated at room temperature for 5 min. The diluted plasmid mixture was then combined with the diluted Lipofectamine 2000 solution, gently mixed, and incubated for an additional 10 min at room temperature. The transfection mixture was added dropwise to the cells, followed by gentle swirling of the plate to ensure even distribution. After 24 h, the culture medium was replaced with 1 mL of fresh medium. At 48 h post‐transfection, the medium containing lentiviral particles was collected and stored on ice. A second batch of medium was collected at 72 h, pooled with the first collection, and filtered through a 0.45 µm PES filter into polypropylene tubes. The filtered virus was aliquoted and stored at −80 °C for future use.

### Preparation of Mouse Primary Neurons

Prior to cell plating, 384‐well plates (Greiner Bio‐One, Cat. #781091) were coated with 0.1 mg mL^−1^ poly‐d‐lysine (Sigma‐Aldrich, Cat. #P0899) in 50 mm borate buffer (pH 8.5) and incubated overnight at room temperature. The following day, plates were washed 5 times with ddH₂O before use.

On the day of dissection, E16.5 embryos were extracted from the uterus and decapitated. The heads were rinsed 4 times in ice‐cold 3+ HBSS (500 mL HBSS (Gibco, Cat. #21‐021‐CM) supplemented with 1% 1 m *N*‐(2‐hydroxyethyl)piperazine‐29‐(2‐ethane‐sulfonic acid) (Gibco, Cat. #15630080), 1% 100 mm sodium pyruvate (Corning, Cat. #25000CI), 0.5% penicillin–streptomycin, and 100 mL ddH₂O) and stored in the same ice‐cold solution. Cortical tissues were dissected following a Nature Protocols method,^[^ ^78^ ^]^ cut into hippocampus‐sized pieces, and stored in ice‐cold Hibernate E solution (Fisher Scientific, Cat. #NC0285514) supplemented with 1% B‐27 Plus (Gibco, Cat. #A3582801) and 1% GlutaMAX.

The cortical tissues were transferred to a tissue culture hood, washed repeatedly with sterile 3+ HBSS in a 15 mL conical tube, and digested with papain‐containing HBSS solution (20 U mL^−1^ papain (Worthington, Cat. #LS003126), 5 mm l‐cysteine, 1.1 mm ethylenediaminetetraacetic acid (pH 8.5)) at 37 °C for 7–10 min. DNase I (Worthington, Cat. #LS006355) was added halfway through digestion. The reaction was quenched with FBS, followed by sequential washes with 3+ HBSS, Neuron Basal Medium (Gibco, Cat. #10888022) and plating medium to remove residual enzymes.

Tissues were gently dissociated into a single‐cell suspension by pipetting in 1 mL plating medium (complete neuronal medium (Neuron Basal Medium supplemented with 2% B‐27 Plus, 1% GlutaMAX, and 1% penicillin–streptomycin) with an additional 5% FBS). The suspension was passed through a cell strainer to remove undissociated cells. Cells were counted, diluted in plating medium, and seeded at 7–9 × 10^3^ cells per well. After ≈3 h, once cells had adhered to the well bottom, the plating medium was replaced with complete neuronal medium (without FBS) to prevent the overgrowth of nonneuronal cell types. Neurons were maintained in a humidified incubator at 37 °C with 5% CO₂.

### Lentivirus and PFF Transduction

Freshly thawed lentivirus was added to primary neurons on DIV2. Puromycin (Sigma‐Aldrich, Cat. #P8833) selection began 48 h later at a concentration of 0.3 µg mL^−1^, by performing a half‐medium change.

On DIV7, PFF seeds were transduced into the neurons. Before transduction, PFFs were diluted in complete neuronal medium and sonicated in a water bath sonicator (Diagenode) for 20 cycles (30 s on, 30 s off) at ≈8 °C. The sonicated seeds were further diluted to 0.5 ng µL^−1^ in complete neuronal medium before being added to the neurons. For 384‐well plates, 5 ng of PFF in 10 µL of medium was added to each well, resulting in a final concentration of 0.1 ng µL^−1^. On DIV12, 20 µL of fresh medium was added to the neurons.

Around DIV17, cells in 384‐well plates were washed repeatedly with PBS using an automated plate washer (Bio‐Tek, ELx405), leaving 30 µL of PBS in each well. To extract soluble proteins and fix the cells, 30 µL of 8% paraformaldehyde (Electron Microscopy Sciences, Cat. #15713S) with 2% Triton X‐100 in PBS was added to each well and incubated at room temperature for 15 min. After fixation, cells were washed with PBS using the plate washer and incubated in blocking buffer (3% bovine serum albumin (Genesee, Cat. #25‐529C) and 3% FBS in PBS) at room temperature for 1 h.

### Immunocytochemistry

To stain insoluble pathological Synuclein, cells were incubated overnight at 4 °C with anti‐α‐Synuclein Phospho (Ser129) Antibody 81A (BioLegend, Cat. #825701) diluted 1:4000 in blocking buffer. Neuronal viability was assessed simultaneously by staining with MAP2 antibody (BioLegend, Cat. #822501) at the same dilution. Following primary antibody incubation, cells were washed with PBS and incubated for 1 h at room temperature with secondary antibodies: goat anti‐mouse IgG2a 568 (Invitrogen, Cat. #A‐21134) and goat anti‐chicken 488 (Invitrogen, Cat. #A‐11039), both diluted 1:2000 in blocking buffer, along with 1 µg mL^−1^ DAPI for nuclear staining. Cells were washed again with PBS to remove unbound secondary antibodies and DAPI before imaging.

### Image Acquisition and Analysis

Images of Synuclein pathology, MAP2, and DAPI were acquired using a Nikon ECLIPSE Ti2‐E inverted microscope. Images were captured at 10 × magnification (0.66 µm px^−1^) with a 2048 × 2048 pixel resolution and 16‐bit depth per channel. RGB TIFFs with channel splits were exported and analyzed in FIJI.

To quantify Synuclein pathology density (pSyn_Den), the 81A channel image was converted to 8‐bit, threshold‐adjusted, and analyzed using the Analyze Particles function to obtain Counts and Integrated Density (IntDen) of each particle. The pathology level was represented by the total IntDen sum. The same function was used to measure the total MAP2‐positive area (MAP2_Area), which served as a normalization factor for Synuclein pathology in neurons. For DAPI counts/area, the watershed algorithm was applied to improve segmentation accuracy. The DAPI counts/area in *RORA*‐expressing cells were compared to control cells to confirm that *RORA* overexpression did not significantly affect cell viability.

The relative Synuclein pathology level in *RORA*‐expressing cells compared to controls was calculated using the following formula

(1) $$\begin{array}{cc} & \mathrm{Relative}\;RORA\;Pathology\\ & \;=\frac{RORA\;\mathrm{signal}-\mathrm{Background}\;\mathrm{signal}}{\mathrm{CTRL}\;\mathrm{signal}-\mathrm{Background}\;\mathrm{signal}}\\ & \;=\frac{\left[\begin{array}{c} \mathrm{pSyn}\_\mathrm{Den}(RORA)/\mathrm{MAP}2\_\mathrm{Area}(RORA)\\ -\mathrm{pSyn}\_\mathrm{Den}(\mathrm{noseeds})/\mathrm{MAP}2\_\mathrm{Area}(\mathrm{noseeds}) \end{array}\right]}{\left[\begin{array}{c} \mathrm{pSyn}\_\mathrm{Den}(\mathrm{CTRL})/\mathrm{MAP}2\_\mathrm{Area}(\mathrm{CTRL})\\ -\mathrm{pSyn}\_\mathrm{Den}(\mathrm{noseeds})/\mathrm{MAP}2\_\mathrm{Area}(\mathrm{noseeds}) \end{array}\right]} \end{array}$$

### Mathematical Models—Global Spread Model

The α‐Syn pathological progression at early stage was considered as the sum of the following two effects:^[^ ^1^, ^2^, ^13^, ^73^ ^]^ 1) Pathology spread—if two brain regions were structurally connected, the pathology could be spread from one region to the other. The spread could be driven by the concentration gradient (diffusion) or against the gradient (active transport). From the cellular level, the spread directionality could be fully anterograde (only from presynaptic membrane to postsynaptic membrane), fully retrograde (only from postsynaptic membrane to presynaptic membrane), or directionally biased. 2) Amplification and clearance—the pathological α‐Syn seeds would induce α‐Syn monomers to generate new misfolded α‐Syn aggregates via templated amplification, and the misfolded α‐Syn aggregates could be removed by autophagy and lysosomal degradation. The total amount of the pathology in the whole network was determined by the overall effects of the amplification and clearance. The degeneration process was not considered, because although some papers had reported the degeneration of α‐Syn pathologies after 8 MPI,^[^ ^17^ ^]^ pronounced degeneration before 6 MPI in the whole brain was not observed.^[^ ^7^, ^73^ ^]^

In our first set of analyses, we augmented the Nex*is*:global model,^[^ ^13^ ^]^ and we applied it to model the two processes in the brain networks. The original Nex*is* model had shown a great performance on predicting the spread of pathological Tau;^[^ ^13^ ^]^ however, it did not account for the possibility of unequal bidirectional spreading. Therefore, the original Nex*is* was augmented to model the spreading directionality.

We first modeled the spread component. Let *X* = {*x_i_* |*i* = 1, 2,…, 410} be the area fractions of the α‐Syn pathology in our 410 gray matter regions, and *C* = {*c_ij_* |*i*,*j* = 1, 2,…, 410} be the weighted adjacency matrix that defined the axonal projection strength from brain region *i* to *j* (connectome adjacency matrix).^[^ ^14^, ^15^ ^]^ First, we considered the case where spread direction was fully anterograde. For each individual brain region *i*, pathology spread from region *j* to *i* along projection *c_ji_*, which increased the pathology burdens of region *i*; meanwhile, pathology also spread from region *i* to *j* along projection *c_ij_*, which decreased the pathology burdens of region *i*. Therefore, the amount of pathologies changed by spread could be described as the following ordinary differential equation (ODE) model

(2) $$\frac{dx_{\mathrm{spread},i}}{dt}=\beta \sum_{j\neq i}\left(c_{ji}x_{j}-c_{ij}x_{i}\right)=-\beta \sum_{j\neq i}\left(c_{ij}x_{i}-c_{ji}x_{j}\right)$$

where β was the global diffusivity rate to modulate the velocity of the pathology diffusion, *t* ∈ *N* denoted the MPI.

Then, we defined the amplification and clearance process using a global parameter α. α was the overall rate of the amplification and clearance effects. We let the positive α denote the overall amplification effect. For each brain region *i*, the rate of change of pathology through amplification and clearance was given by

(3) $$\frac{dx_{\mathrm{amplification}/\mathrm{clearance},i}}{\mathrm{dt}}=\alpha x_{i}$$

We the fully anterograde global spread model, then, was governed by the sum of these two equations

(4) $$\frac{dx_{i}}{\mathrm{dt}}=\frac{dx_{\mathrm{amplification}/\mathrm{clearance},i}}{\mathrm{dt}}+\frac{dx_{\mathrm{spread},i}}{\mathrm{dt}}=\alpha x_{i}-\beta \sum_{j\neq i}(c_{\mathrm{ij}}x_{i}-c_{\mathrm{ji}}x_{j})$$

We defined the diagonal operation $\mathrm{diag}\left(Z\right)=\left(\begin{array}{ccccc} z_{1} & 0 & 0 & \text{…} & 0\\ 0 & z_{2} & 0 & \text{…} & 0\\ 0 & 0 & z_{3} & \text{…} & 0\\ \text{…} & \text{…} & \text{…} & \text{…} & 0\\ 0 & 0 & 0 & \text{…} & z_{n} \end{array}\right)$ for *Z* = (*z*_1_,*z*_2_,…, *z_n_*), and let *columnsum* denote the sums of every column of a given matrix. Then, Equation (4) had an analytical solution: $\hat{X}\left(t\right)=e^{(diag(\alpha )-\beta L)t}X_{0}$. Here, $\hat{X}\left(t\right)$ was the vector of the predicted pathology, *L* was the Laplacian operator of the connectome adjacency matrix. For for the fully anterograde spreading case, *L* = *L*_anterograde_ = diag(columnsum(*C*′)) − *C*′  and $L_{ij}=\{\begin{array}{c} \sum_{j\neq i}c_{ij},i=j\\ -c_{ji},i\neq j \end{array}$. $X_{0}=\{\begin{array}{c} \gamma ,\mathrm{injected}\;\mathrm{areas}\\ 0,\mathrm{others} \end{array}$ was the seed scaling vector that described the amount of the injected pathological seeds at 0 MPI, which were 0 for non‐injected regions and γ ∈ (0, 1] for the injected regions.

Similarly, for the fully retrograde diffusion case, there was only one different term

(5) $$L_{\mathrm{retrograde}}=diag\left(columnsum\left(C\right)\right)-C$$

In order to accommodate unequal bidirectional spread, we introduced the parameter *s*, which was bounded between 0 and 1. By convention, a value of 0 indicated fully anterograde spread, a value of 1 indicated fully retrograde spread, and a value of 0.5 indicated unbiased spreading. The spread Equation (2) was therefore generalized as follows

(6) $$\begin{array}{cc} \frac{dx_{\mathrm{spread},i}}{dt} & =\beta \sum_{j\neq i}\left[\left(1-s\right)\left(c_{ji}x_{j}-c_{ij}x_{i}\right)+s\left(c_{ij}x_{j}-c_{ji}x_{i}\right)\right]\\ & =-\beta \sum_{j\neq i}\left[\left(1-s\right)\left(c_{ij}x_{i}-c_{ji}x_{j}\right)+s\left(c_{ji}x_{i}-c_{ij}x_{j}\right)\right] \end{array}$$

The general Laplacian operator, $\hat{L}$, was therefore defined as

(7) $$\left\{\begin{array}{c} \hat{L}=diag\left(columnsum\left(\hat{C}\right)\right)-\hat{C}\\ \hat{C}=\left(1-s\right)\cdot C^{\prime }+s\cdot C \end{array}\right.$$

As before, this equation had the following analytical solution

(8) $$\hat{X}\left(t\right)=e^{(diag\left(\alpha \right)-\beta \hat{L})t}X_{0}$$

Compared to the previous mathematical models in this field,^[^ ^7^, ^9^, ^10^, ^11^, ^12^, ^13^, ^17^, ^21^ ^]^ our augmented Nex*is* model was able to simultaneously capture the seed scaling, the global amplification or clearance effect, the diffusion effect, and the directionally biased spread of pathology, whereas the other studies were only modeling parts of the above factors, especially ignoring the possibility of directionally biased spread.

### Mathematical Models—Spread Model with the Effect of Gene Expression

In addition to the connectome‐based spread and the global amplification and clearance effects, it was reported that the cell‐type densities^[^ ^1^ ^]^ and regional gene expression^[^ ^7^, ^17^, ^19^ ^]^ also played very important roles in the pathology progression; this is known as the selective vulnerability of different brain regions imparted by genes. To investigate this effect, and especially to identify the candidate genes that were associated with the pathological spread, we explored the effects of the 3855 individual genes from the coronal series of the AGEA.^[^ ^13^, ^16^ ^]^ We modified the Nex*is*:microglia model^[^ ^13^ ^]^ explored previously to accommodate the differential effects of outgoing and incoming connections as follows.

Let $G_{k}=\left[\begin{array}{ccccc} g_{k,1} & 0 & 0 & \text{…} & 0\\ 0 & g_{k,2} & 0 & \text{…} & 0\\ 0 & 0 & g_{k,3} & \text{…} & 0\\ \text{…} & \text{…} & \text{…} & \text{…} & \text{…}\\ 0 & 0 & 0 & \text{…} & g_{k,410} \end{array}\right]$, where *g*_*k*,*i*_ was the gene expression level of gene *k* on the brain region *i*. Then, we defined the gene‐mediated connectivity matrix, *C*⊗*G_k_*, which could take different functional forms depending on whether the effect on presynaptic or postsynaptic sites was being considered. Using this nomenclature, previous studies had defined *C*⊗*G_k_* = *G_k_* · *C*, and therefore had only explored the gene mediation of pathology spread along outgoing connections (Figure 4B).^[^ ^7^, ^13^, ^17^ ^]^ Biologically, these models only investigated the selective vulnerability from the gene expression of presynaptic regions (Figure 4B). In order to generalize the model to also accommodate modulation from incoming regions alone or equally from both the outgoing and incoming connections simultaneously, we have considered the following three forms of *C*⊗*G_k_* (Figure 4A–D)

(9) $$C⊗G_{k}=\left\{\begin{array}{c} G_{k}\cdot C,\mathrm{outgoing}\;\mathrm{effect}\\ C\cdot G_{k},\mathrm{incoming}\;\mathrm{effect}\\ \sqrt{G_{k}}\cdot C\cdot \sqrt{G_{k}},\mathrm{combined}\;\mathrm{effect} \end{array}\right.$$

Therefore, the direction‐biased spreading of the gene–connectome matrices were updated as ${\hat{C}}_{\mathrm{Gene}}=\left(1-s\right)\cdot {\left(C⊗G_{k}\right)}^{\prime }+s\cdot \left(C⊗G_{k}\right)$, and its related Laplacian matrices were then updated as ${\hat{L}}_{\mathrm{Gene}}=\mathrm{diag}(\mathrm{columnsum}\left({\hat{C}}_{\mathrm{Gene}}\right))-{\hat{C}}_{\mathrm{Gene}}$. Then, the solution could be calculated by

(10) $$\hat{X}\left(t\right)=e^{(diag\left(\alpha \right)-\beta {\hat{L}}_{Gene})t}X_{0}$$

### Mathematical Models—Model Fitting with the Quantitative Pathological Data

We took the average of the 3 and 6 MPI's quantitative pathological data respectively, and fitted the above models over *t* ∈ {3, 6}. We picked the seed scaling parameter γ, the overall effect of global amplification or clearance α, the global diffusivity rate β, and the directionality parameter *s*, which maximized the model fitting, defined as the best CCC^[^ ^24^ ^]^ between the quantitative pathology vector log _10_(*X*(*t*)) and the predicted pathology vector $\log_{10}\left(\hat{X}\left(t\right)\right)$ for all nonzero and defined values of *X*(*t*), averaged over *t* ∈ {3, 6}. Compared to Pearson's correlation coefficient method as a loss function, CCC can capture the scale characteristics^[^ ^24^ ^]^ and be used to fit the seed scaling parameter γ. We took the logarithm transformation (log10‐transformed), since the scale of the quantitative pathology data in different regions could differ by a factor of 1–1000. According to the above models, the parameters had different bound limitations. Using the MATLAB (R2022a, MathWorks) function “fmincon”, we fitted the parameters of the model using the following cost function

(11) $$\begin{array}{c} \underset{\alpha ,\beta ,\gamma ,s}{\arg\min}\sum_{t\in \left[3,6\right]}(1-\mathrm{CCC}\left(\mathrm{lo}g_{10}\left(X\left(t\right)\right),\mathrm{lo}g_{10}\left(\hat{X}\left(t\right)\right)\right),\;s.t.\;\;\left\{\begin{array}{c} \gamma \in (0,1)\\ \alpha \in (-\infty ,+\infty )\\ \beta \in [0,+\infty )\\ s\in [0,1] \end{array}\right. \end{array}$$

The model ran successfully for all genes in the outgoing or incoming effect models, but 60 genes failed to be fitted in the combined effect due to the error with matrix computations (3795 out of 3855 genes retained, see Table S2 in the Supporting Information for details).

### Mathematical Models ‐ Robustness Testing of Mathematical Models

Bootstrapping was used to assess robustness. To prove that pathology progression was driven by the mesoscale connectome but not by randomly generated matrices, we tested the global spread model with 1000 null connectomes whose elements were sampled from the uniform random distribution *U*_[0,1]_. We also explored the effect of randomly permuting the actual connectome weights, creating a different set of 1,000 null connectomes. To investigate whether the partial connectome was able to explain the pathology progression, and also to explore the characteristics of the key partial connectome, we fit the global spread model using connectomes with specified percentages of connections removed at random, with each 5% increment sampled 1,000 times.

We also tested the robustness of the genes using the 500th genes of each spread effect (*Large* for the outgoing effect, *Socs6* for the incoming effect, and *Tbc1d14* for the combined effect) as test cases. The bootstrapping was repeated 1000 times by randomly permuting the elements of the gene expression vectors.

The random seeds (“rng” function in MATLAB software) were incorporated to ensure that the results of bootstrapping were reproducible.

### Mathematical Models ‐ Model Validation Using Other Datasets

In order to validate our findings of spreading directionality and key connectomes, we ran our model using external datasets from Henderson et al.^[^ ^7^ ^]^ Similar to our experiment, the amount of 5 µg mouse α‐Syn PFFs in 2.5µL Dulbecco's PBS were injected into the dorsal striatum of the C57BL/6J nontransgenic mice (NTG mice, same as the wild‐type mice in our study) and the B6.Cg‐Tg(Lrrk2*G2019S)2Yue/J *G2019S LRRK2* transgenic mice (*G2019* mice). Mice were euthanized at 1, 3, and 6 monthspost‐injection (NTG mice: *n* = 4 for 1 MPI, *n* = 6 for 3 MPI, and *n* = 6 for 6 MPI. *G2019* mice: *n* = 6 for 1 MPI, *n* = 6 for 3 MPI, and *n* = 7 for 6 MPI), and all mice were initially stained with Syn506 for quantification of pathology. After brain annotation and quantification, α‐Syn pathology distribution data and a connectome of 116 annotated brain regions (13 456 connections in total, denoted as the “masked” connectome) were used to model the pathological progression according to the above global spread model. It was noted that compared with our whole connectome (410 brain regions with 168 100 connections in total), the “masked” connectome in Henderson et al.^[^ ^7^ ^]^ only represented a subset of all connections in the brain. The detailed dataset and experimental design could be found in Henderson et al.^[^ ^7^ ^]^ and on GitHub (https://github.com/ejcorn/connectome_diffusion).

### Systems Biology Analyses—Candidate Genes Selection

After fitting the above models, we ranked the importance of the genes for their outgoing, incoming, and combined effects, separately. Any individual gene related to a specific spread effect will have an Ave. CCC values for its best fitting, and the genes which had better Ave. CCC values than that of the global spread model were considered as the potential genes which affected the spread positively. We picked the top 500 genes from the potential gene lists for each effect group as the candidate genes. We then performed gene ontology analyses, cell‐type analyses, and gene regulatory network key driver analyses.

### Systems Biology Analyses—Gene Ontology Analyses

Gene ontology (GO) analyses were performed in PANTHER to study the enrichments in biological process, molecular function, and cellular component of the candidate genes (PANTHER Overrepresentation Test (Released 20221013); GO Ontology database https://doi.org/10.5281/zenodo.7709866 Released 2023‐03‐06; http://geneontology.org/).^[^ ^29^ ^]^ For each GO category of any specific spread effect, we visualized the results sorted by fold enrichment that were statistically significant after Bonferroni correction.

### Systems Biology Analyses—Cell‐Type Analyses

Marker genes for neuronal cell types (excitatory and inhibitory) and glial cell types (astrocyte, microglia, oligodendrocyte, oligodendrocyte progenitor cell, and ependyma) were used to test for enrichment in the candidate genes of the incoming, outgoing, and combined effects. Excitatory and inhibitory neuronal marker genes were collected from the study on cell type compositions across the entire mouse brain.^[^ ^33^ ^]^ Distinguishing markers from different excitatory (2426 cell types) and inhibitory (2242 cell types) neurons were combined into excitatory and inhibitory marker gene lists. As glial cell marker genes are more similar across brain regions, an internal single‐cell RNA‐sequencing mouse dataset (see Table S7 in the Supporting Information for details) was used to retrieve marker genes for astrocyte, microglia, oligodendrocyte, oligodendrocyte precursor cell, and ependyma consistent between the hippocampus and hypothalamus (average log2FC > 2 compared to other cell types for each tissue) which captured canonical glial cell Stype marker genes. Marker gene lists were subset to the genes used for testing of the model. Hypergeometric test was used to retrieve the significance of enrichment, and *p* values were Bonferroni adjusted. The background population size was the number of genes (3855) tested in the model.

### Systems Biology Analyses—KD Analyses and Regulatory Network

We used SCING^[^ ^34^ ^]^ to build gene regulatory networks for candidate gene subsets. We used the default hyperparameters in SCING that included all genes, 10 principal components, 0.7 subsampling proportion, 100 neighbors per gene, and 500 target supercells. Single cell transcriptomics data from Tabula Muris,^[^ ^79^ ^]^ Tabula Muris Senis,^[^ ^80^ ^]^ and Mouse Cell Atlas,^[^ ^81^ ^]^ with networks built on the Allen 10X dataset^[^ ^82^ ^]^ from the hippocampus, visual cortex, somatosensory cortex, and primary motor cortex brain regions were used for outgoing effect candidate genes, since outgoing effect candidate gene was enriched in glial cells. Then, to study the gene regulatory relationship in neuronal network, one neuronal SCING network was built based on the neuronal single cell transcriptomics data from Allen Brain Atlas,^[^ ^83^ ^]^ Tabula Muris,^[^ ^79^ ^]^ Tabula Muris Senis,^[^ ^80^ ^]^ and Mouse Cell Atlas,^[^ ^81^ ^]^ for each candidate gene of three effects (outgoing, incoming, combined) to the neuronal cell types and the resulting networks were combined together as a union network with the data source information retained. This union network along with the candidate genes of the incoming, outgoing, and combined effects were used as inputs into key driver analysis (KDA)^[^ ^35^ ^]^ using a search depth of 1 and not considering direction to identify KDs whose neighborhood subnetworks were enriched for the candidate genes of each category (incoming, outgoing, combined) based on a Chi‐like statistics. We called key drivers with a false discovery rate (FDR) threshold of less than 0.05.

To test whether KDs for the candidate genes of the same effect category (incoming, outgoing, or combined) were more connected than KDs for candidate genes of different categories, we performed chi‐square goodness of fit tests for each KD effect, with the observations being the number of the directed edges from a source KD node of each effect to its respective target KD nodes. As each node could fall under multiple effects, we used two effects for each edge: target was of the same effect or not. The expected number of edges for each KD to the same source type was calculated by $|\mathrm{Edge}s_{KD_{\mathrm{source}}\to KD_{\mathrm{any}}}|*\frac{|KD_{\mathrm{source}}|-1}{|KD_{\mathrm{any}}|-1}$ based on the number of edges for each effect category found in the networks and the expected probabilities for each KD based on the gene metadata if edge selection was independent. The remaining edges were expected to not be of the source KD type.

### Systems Biology Analyses—GWAS‐PD Enrichment Analyses

We performed enrichment analysis on the genes in each network (incoming, outgoing, combined, and the union network of all three) for over‐representation of PD candidate risk genes from the GWAS Catalog^[^ ^36^ ^]^ using hypergeometric tests^[^ ^84^ ^]^ with Bonferroni correction for *n* = 4.

### Software and Statistics

All statistical tests were two‐tailed, and the significance level was set to α = 0.05 unless otherwise noted. Normality was assessed using the Shapiro–Wilk test. Student's *t*‐test (for normally distributed data) or Wilcoxon rank‐sum test (for non‐normally distributed data) was used for comparing pathology between 3 and 6 MPI for each quantified brain region. One sample Wilcoxon test was used to compare the relative α‐Syn inclusions in control and *RORA*‐overexpressing groups. Bonferroni correction and the FDR were used for multiple comparisons in systems biology analyses. Chi‐square test was used for comparing the actual number of the edges with the expected number after KD analyses. All of the *p* values were adjusted accordingly. All statistical analyses and visualization were performed mainly with MATLAB (R2022a), Graphpad Prism (8.0.2, GraphPad Software), R (4.1.3), and Python (3.9.11).

## Conflict of Interest

The authors declare no conflict of interest.

## Author Contributions

Y.L. and J.T. contributed equally to this work as co‐first authors. A.R., and C.P. contributed equally to this work. Y.L. conceived and designed the experiments, collected data, performed modeling and coding, analyzed and visualized the results, wrote the original draft, and edited the draft. J.T. conceived and designed the experiments, performed modeling and coding, analyzed and visualized the results, wrote and edited the paper. S.Z. performed primary neuron culture experiments and analyzed the data. J.D. performed gene network and cell‐type enrichment analyses, and visualized the results. N.W. performed GWAS‐PD enrichment analyses and visualized the results. C.L. and S.K. performed annotations and quantifications for brain sections, collected data, analyzed the results. C.A. collected data. J.T. and M.C. curated multiple single cell Atlases and constructed cell‐type specific SCING networks. C.L. and B.L. analyzed and visualized the results. Y.S. revised the paper. R.D. contributed to functional study of *RORA* in pathological α‐Syn transmission. X.Y. supervised, conceived, and designed the systems biology analyses, and edited the paper. A.R. supervised the study, conceived and designed the experiments, wrote and edited the draft. C.P. planned and supervised the study, conceived and designed the experiments, interpreted the data, wrote and edited the draft. All authors reviewed and approved the paper.

## Supporting information



Supporting Information

Supporting Information

## Data Availability

The data that support the findings of this study are available in the supplementary material of this article.
