# Supplementary material for: Key Connectomes and Synaptic‐Compartment‐Specific Risk Genes Drive Pathological α‐Synuclein Spreading
Source: Adv Sci (Weinh). 2025 May 28;12(25):2413052. doi: 10.1002/advs.202413052 (PMC12224943; doi:10.1002/advs.202413052)
Supplement: Supplementary file 1 — Supporting Information [file ADVS-12-2413052-s001.docx]

**Supplementary Figures**


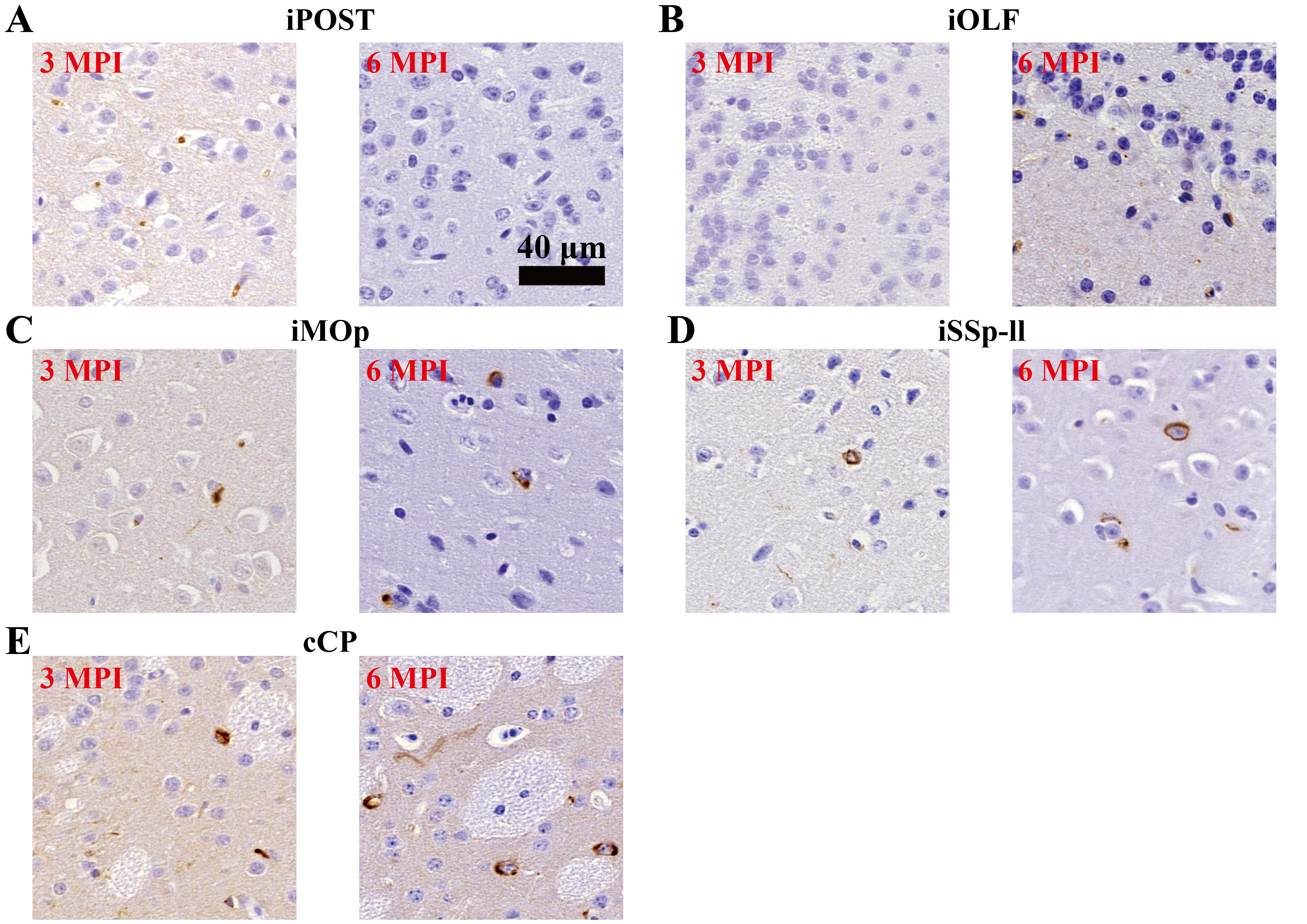


**Figure S1** Representative quantitative pS129 α-synuclein pathology images of selected regions at 3 and 6 MPI. **A**, iPOST region, showing a decrease trend from 3 to 6 MPI. **B-E**, Regions with statistically significance between 3 and 6 MPI after FDR correction. **B**, iOLF region. **C**, iMOp region. **D**, iSSp-ll region. **E**, cCP region. All scales were the same and the scale bar was shown in **A**. Scale bar: 40 μm.


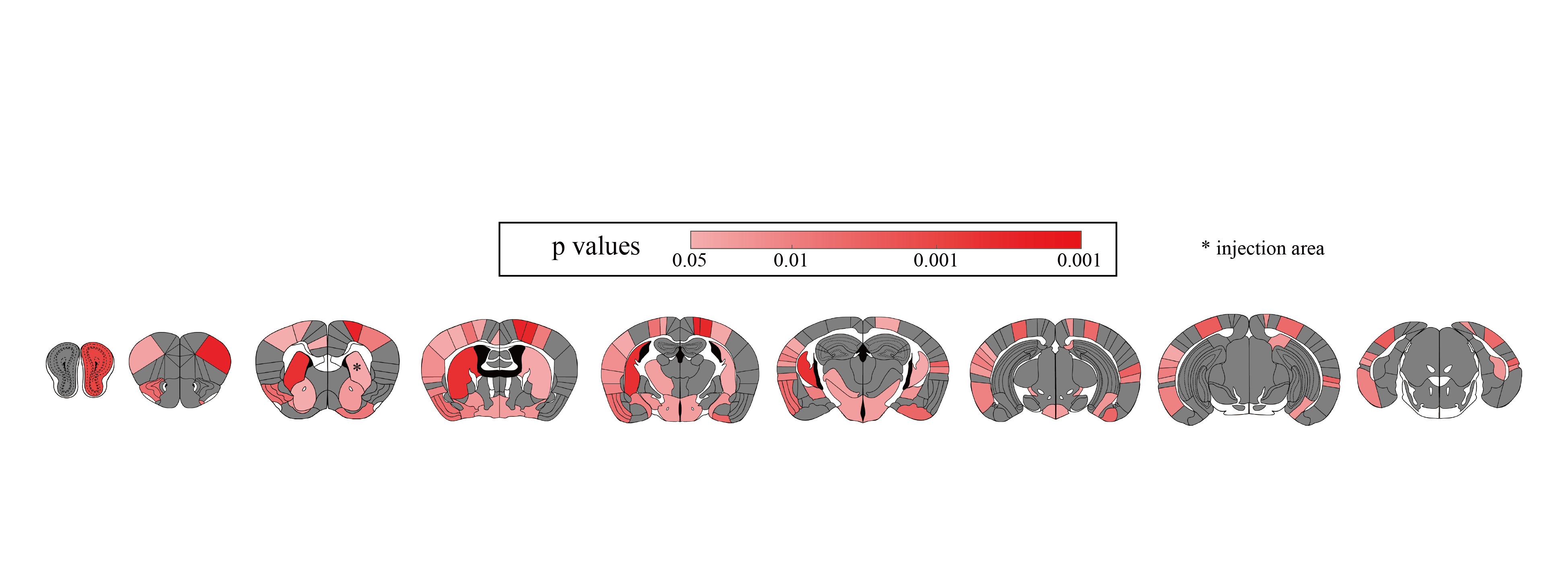


**Figure S2** Heatmap showing regional *p* values with statistically significant (*p*<0.05) differences between 3 and 6 MPI. Warmer colors represented the differences between 3 and 6 MPI were more statistically significant (*, injection area).


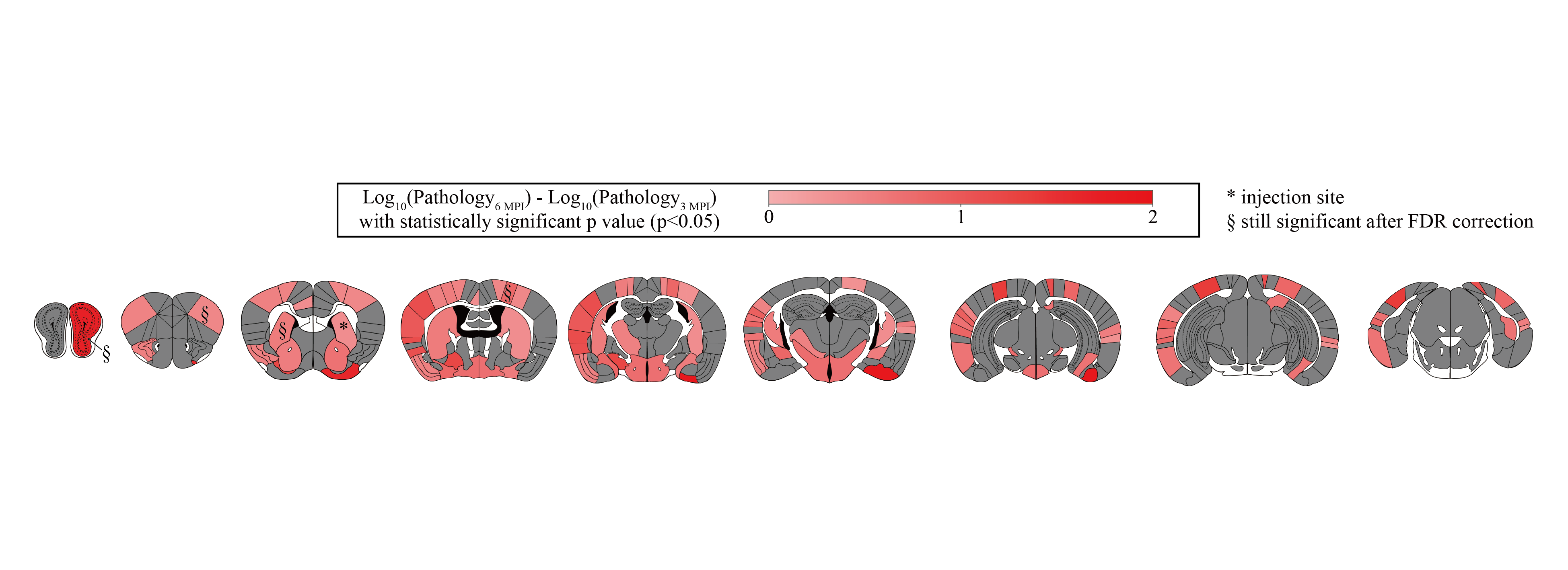


**Figure S3** Differences of regional pathology burdens between 3 and 6 MPI, only displaying the results of statistically significant regions. All the statistically significant regions showed upward trends of pathology. iOLF, iMOp, iSSP-ll, and cCP were still significant after false discovery rate (FDR) correction. *, injection area. §, regions that were still significant after FDR correction.


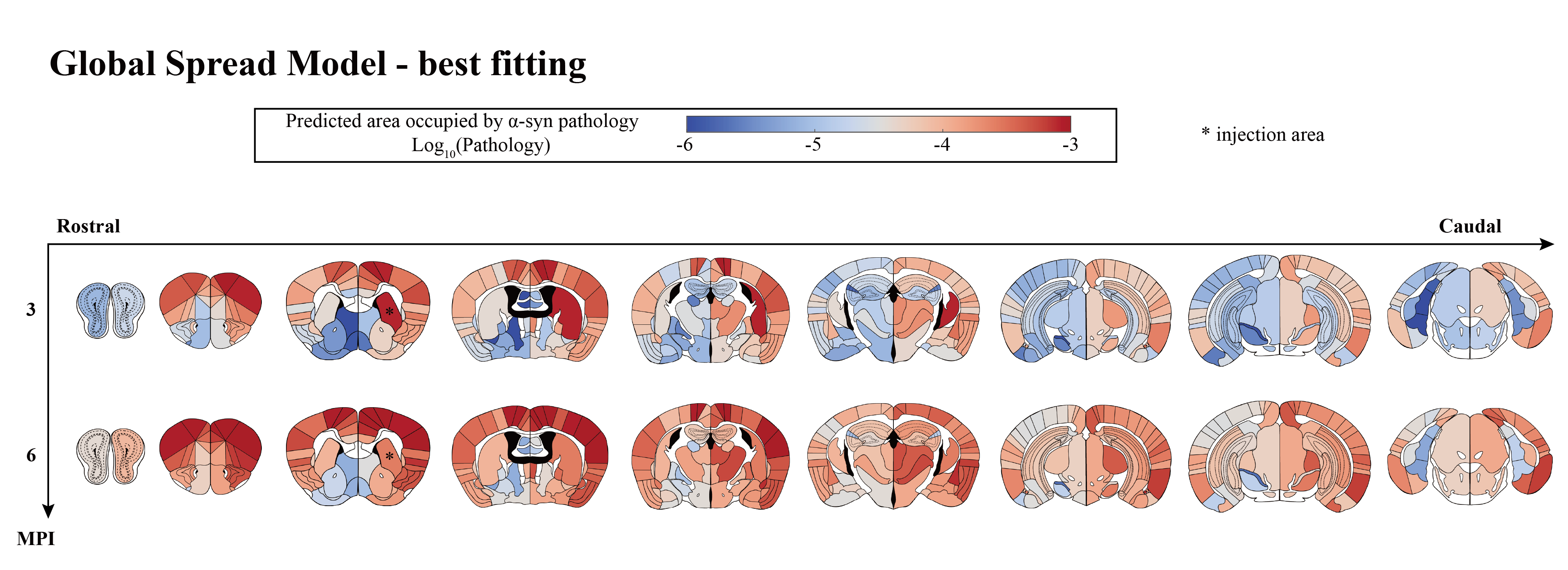


**Figure S4** Heatmap of the regions affected by α-Syn pathologies calculated by the best model fitting of global spread model (log10-transformed; *, injection area).


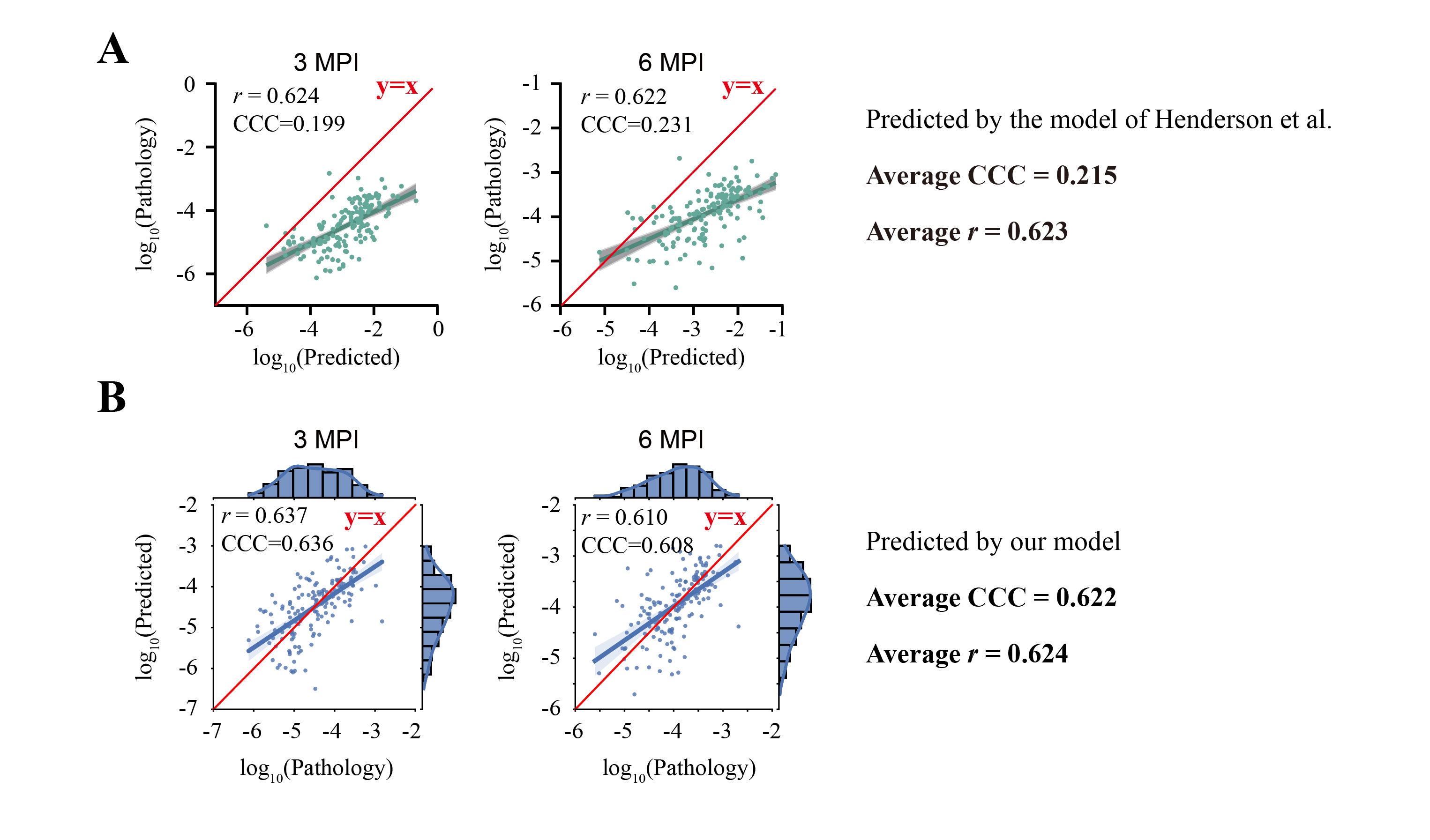


**Figure S5** Comparison between the model fitting using a previous model (**A**) from Henderson et al. (Henderson et al., 2019) and our model (**B**, which reproduces **Fig. 2A**) based on our quantitative pathology data. Compared to our model, the model of Henderson et al. disregarded the amplification process of pathology and the unequally directional transmission, and used the best Pearson’s correlations to evaluate the model performance, which significantly compromised the ability to predict both trend and scale compared to our current model. Note that the closer the best-fit line is to y=x (red line), the better the model captures scale of the observed data.


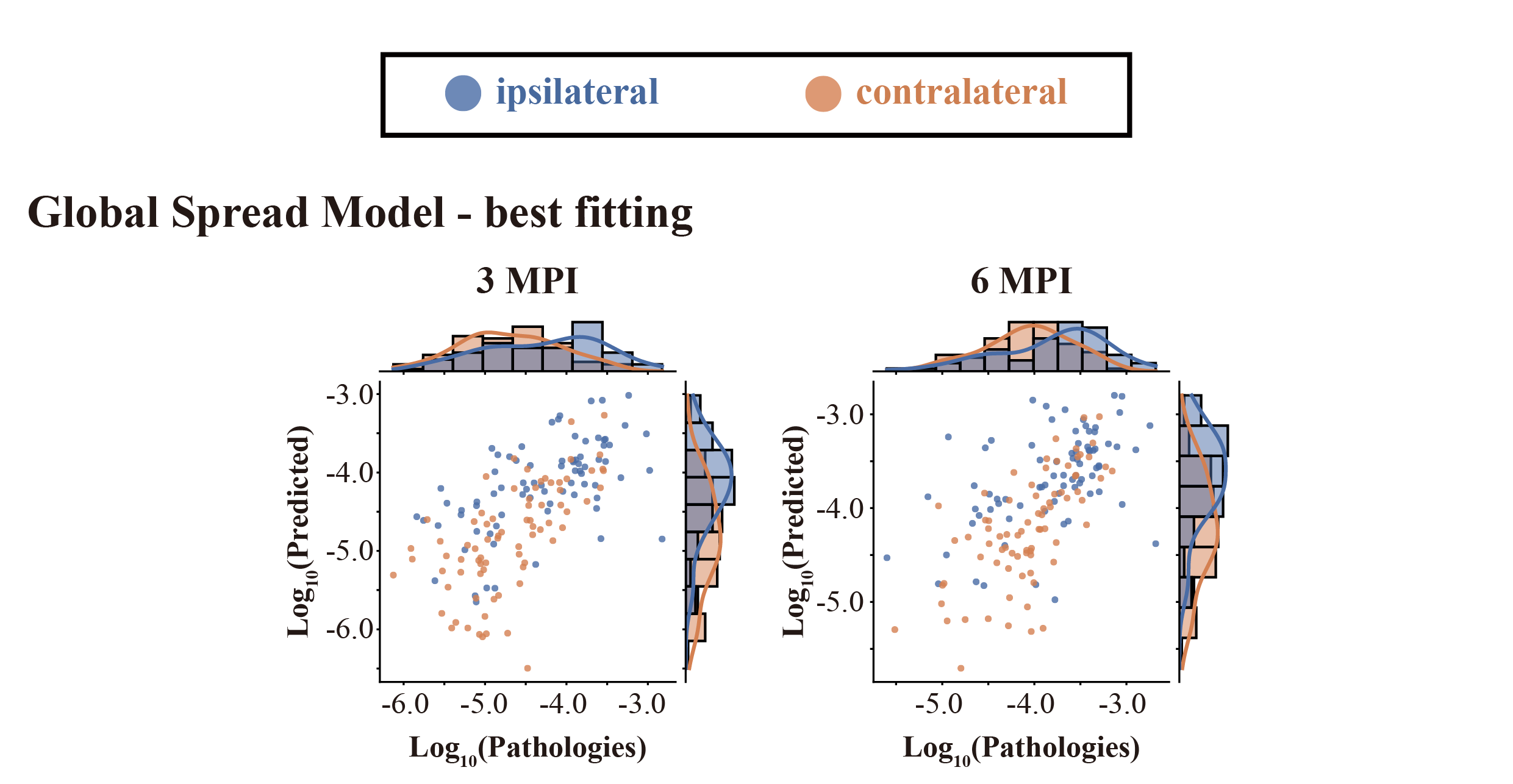


**Figure S6** The global spread model accurately captured the feature that the ipsilateral hemisphere had more pathology than the contralateral hemisphere both at 3 and 6 MPI. Regions ipsilateral to the injection site are shown in blue, while contralateral regions are shown in orange, with histograms of the univariate distributions shown along the axes.


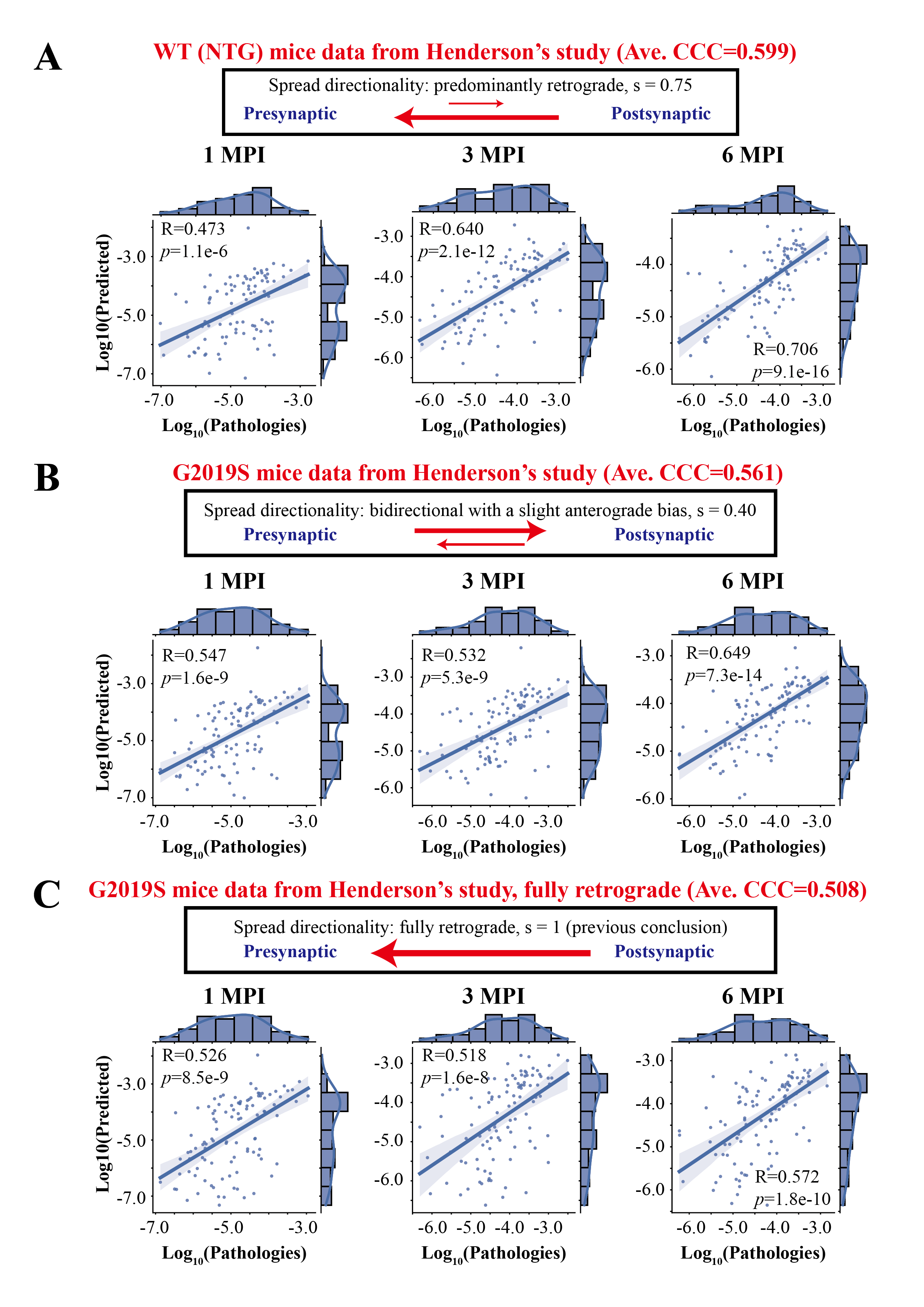


**Figure S7** Wild-type non-transgenic (WT) mice and *G2019S LRRK2* (*G2019S*) mice exhibit different directional preferences of pathological α-Syn spread. We used data from Henderson et al. (Henderson et al., 2019) and our global spread model (see **Methods**). **A**, Best model results for the WT mice (Ave. CCC = 0.599) with the directionality parameter of s=0.75 (predominantly retrograde, in line with **Fig. 2A**). **B**, Best model results for the *G2019S* mice (Ave. CCC = 0.561) with the directionality parameter of s=0.40 (bidirectional, with a slight anterograde bias). **C**, Model results for the *G2019S* mice (Ave. CCC=0.508) with s=1, which enforces fully retrograde spread as in previous models (Henderson et al., 2019) . The directionality of pathological α-Syn spread is shown by the arrows in the box, while the length of the line indicates the proportion of spread direction. Each dot represents one brain region and the x-axis and y-axis represent the pathology (log10-transformed) found empirically and predicted by the model, respectively. For each situation, the Pearson’s correlation coefficient and the best regression lines for 1, 3 and 6 MPI are also displayed. The shaded ribbon represented the 95% prediction interval. Abbreviations: R, Pearson’s correlation coefficient; *p*, *p* values from linear regression. The detailed description of the datasets was shown in **Methods**.


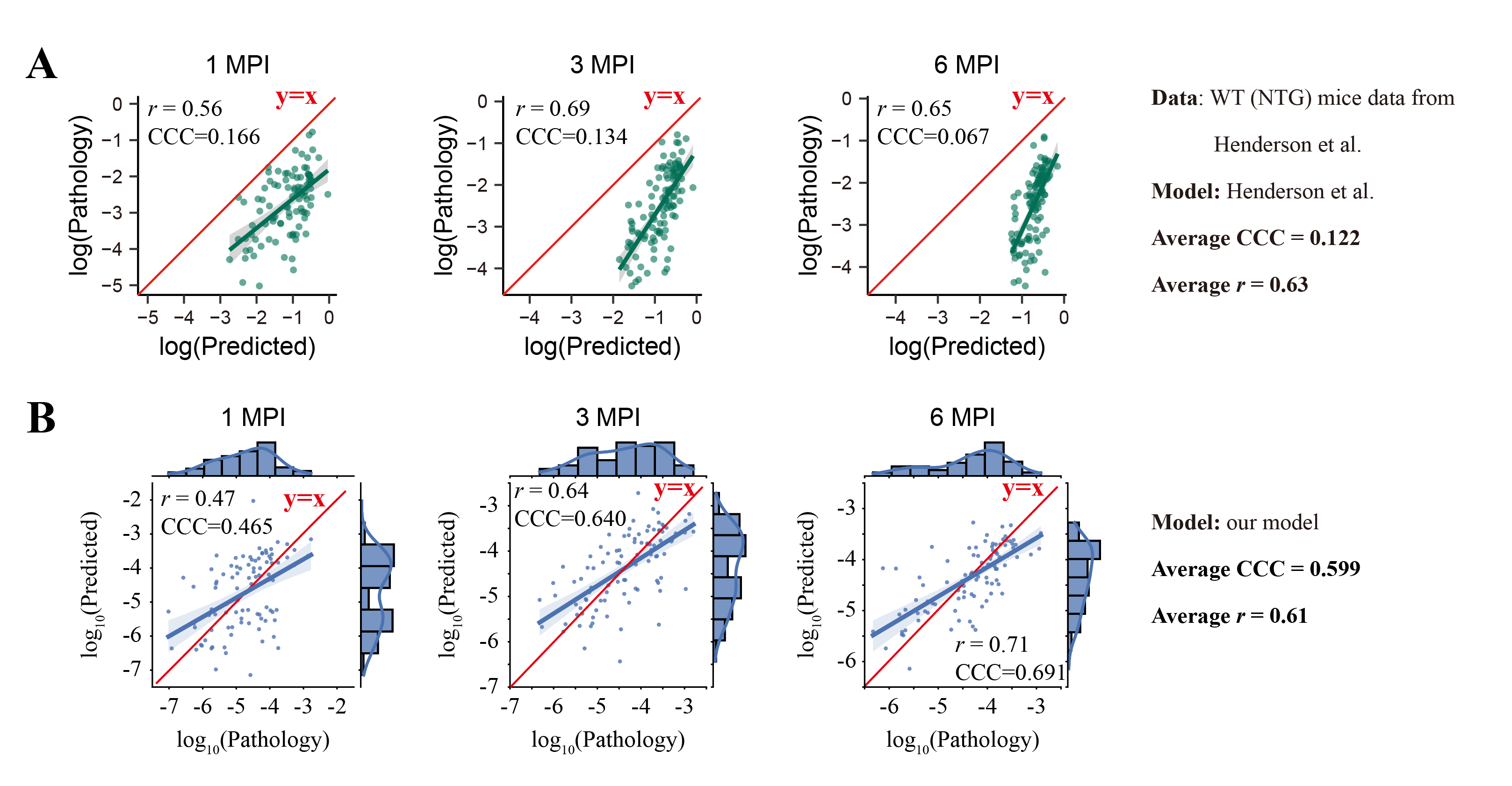


**Figure S8** Comparison between the model fitting using a previous model (**A**) from Henderson et al. (Henderson et al., 2019) and our model (**B**, which reproduces **Fig. S7A**) based on the WT (*NTG*) mice data from Henderson et al. (Henderson et al., 2019). Compared to our model, the model of Henderson et al. disregarded the amplification process of pathology and the unequally directional transmission, and used the best Pearson’s correlations to evaluate the model performance, which significantly compromised the ability to predict both trend and scale compared to our current model. Note that the closer the best-fit line is to y=x (red line), the better the model captures scale of the observed data.

**
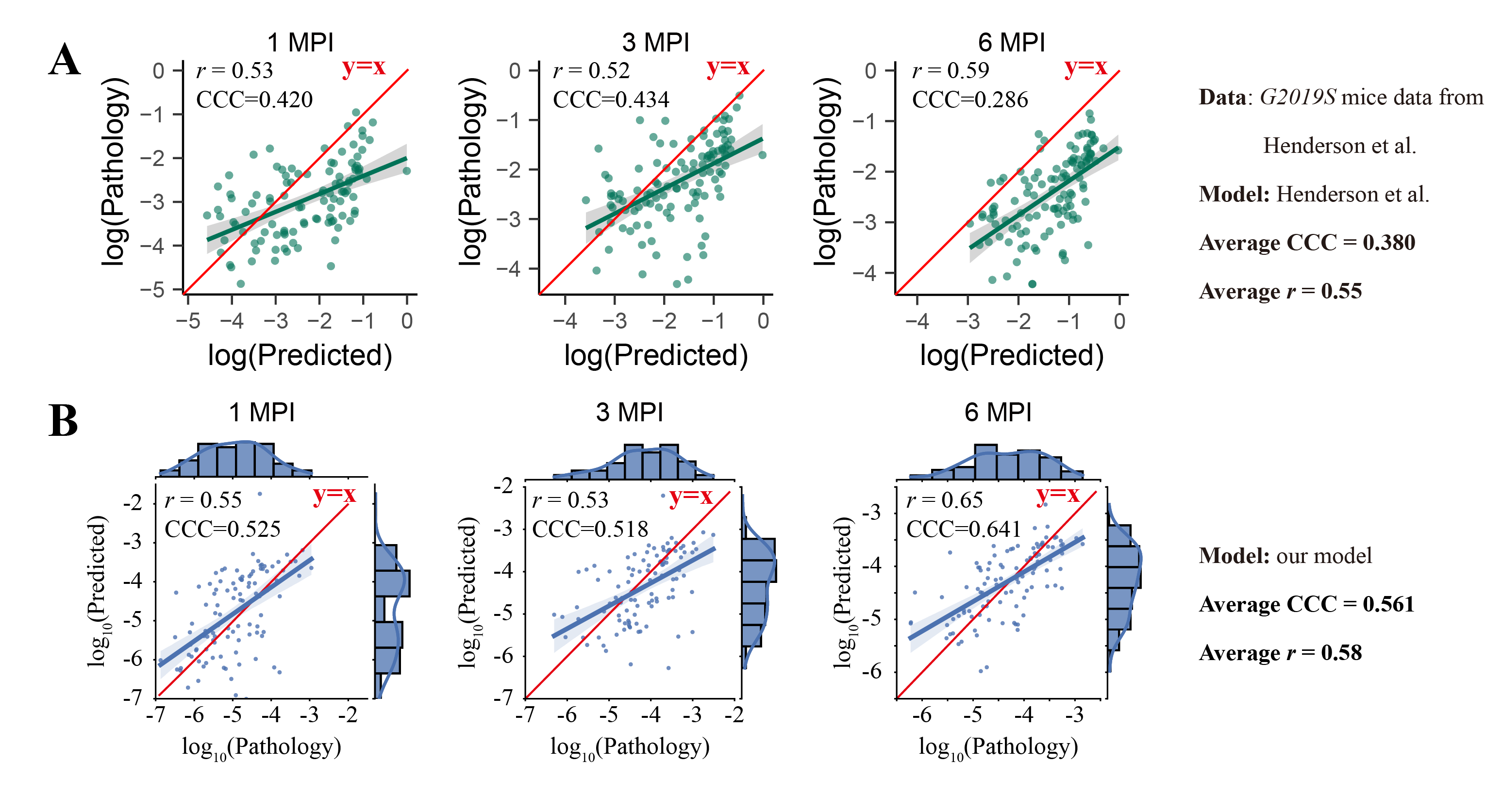
**

**Figure S9** Comparison between the model fitting using a previous model (**A**) from Henderson et al. (Henderson et al., 2019) and our model (**B**, which reproduces **Fig. S7B**) based on the *G2019S-LRRK2* (*G2019S*) mice data from Henderson et al (Henderson et al., 2019). Compared to our model, the model of Henderson et al. disregarded the amplification process of pathology and the unequally directional transmission, and used the best Pearson’s correlations to evaluate the model performance, which significantly compromised the ability to predict both trend and scale compared to our current model. Note that the closer the best-fit line is to y=x (red line), the better the model captures scale of the observed data.


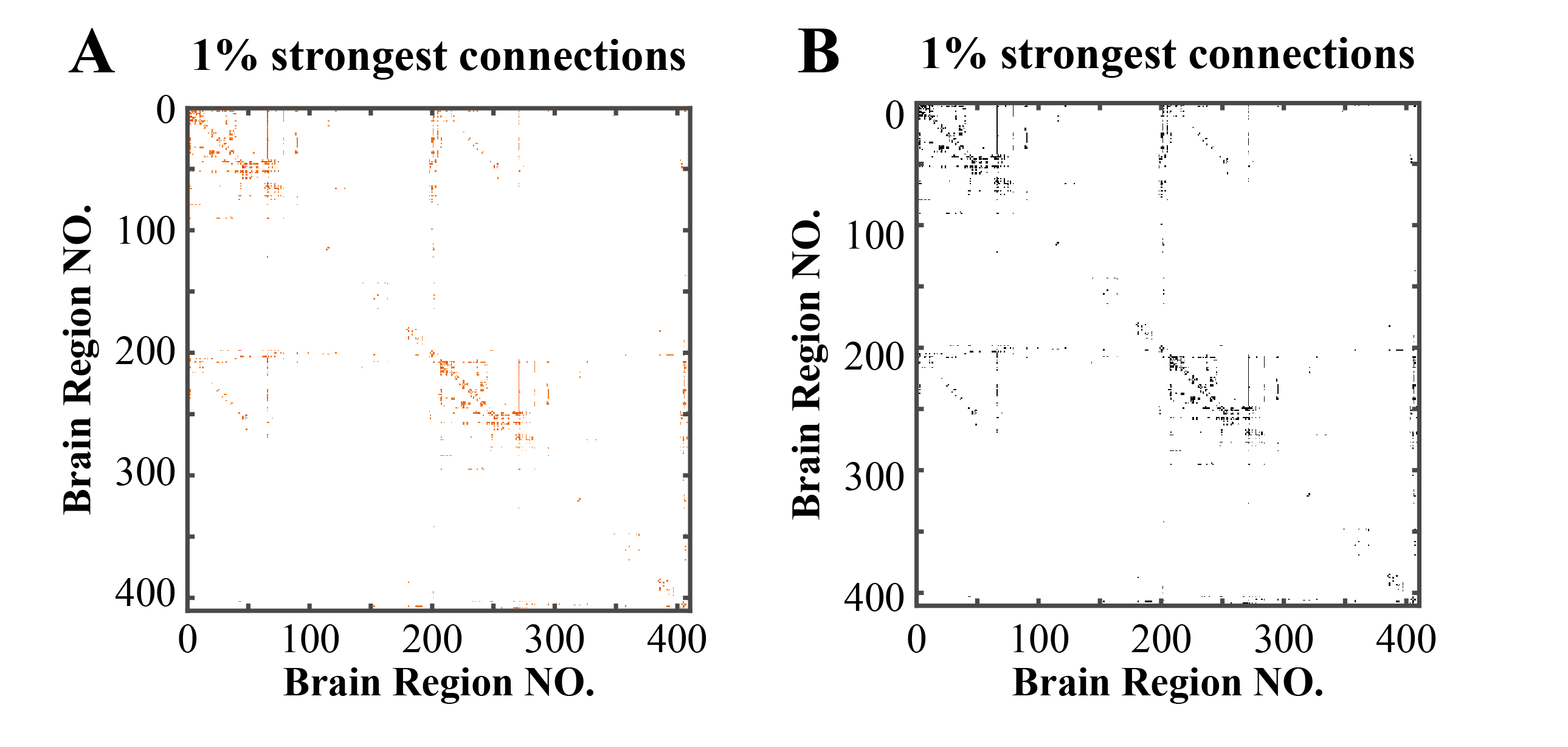


**Figure S10** Visualization of connectomes of the 1% of the strongest connections. **A**, Heatmap of this partial connectome, where the warmer colors represent stronger connections (log10(raw_value+1)-transformed). **B**, Heatmap of the adjacency matrix corresponding to **A**.


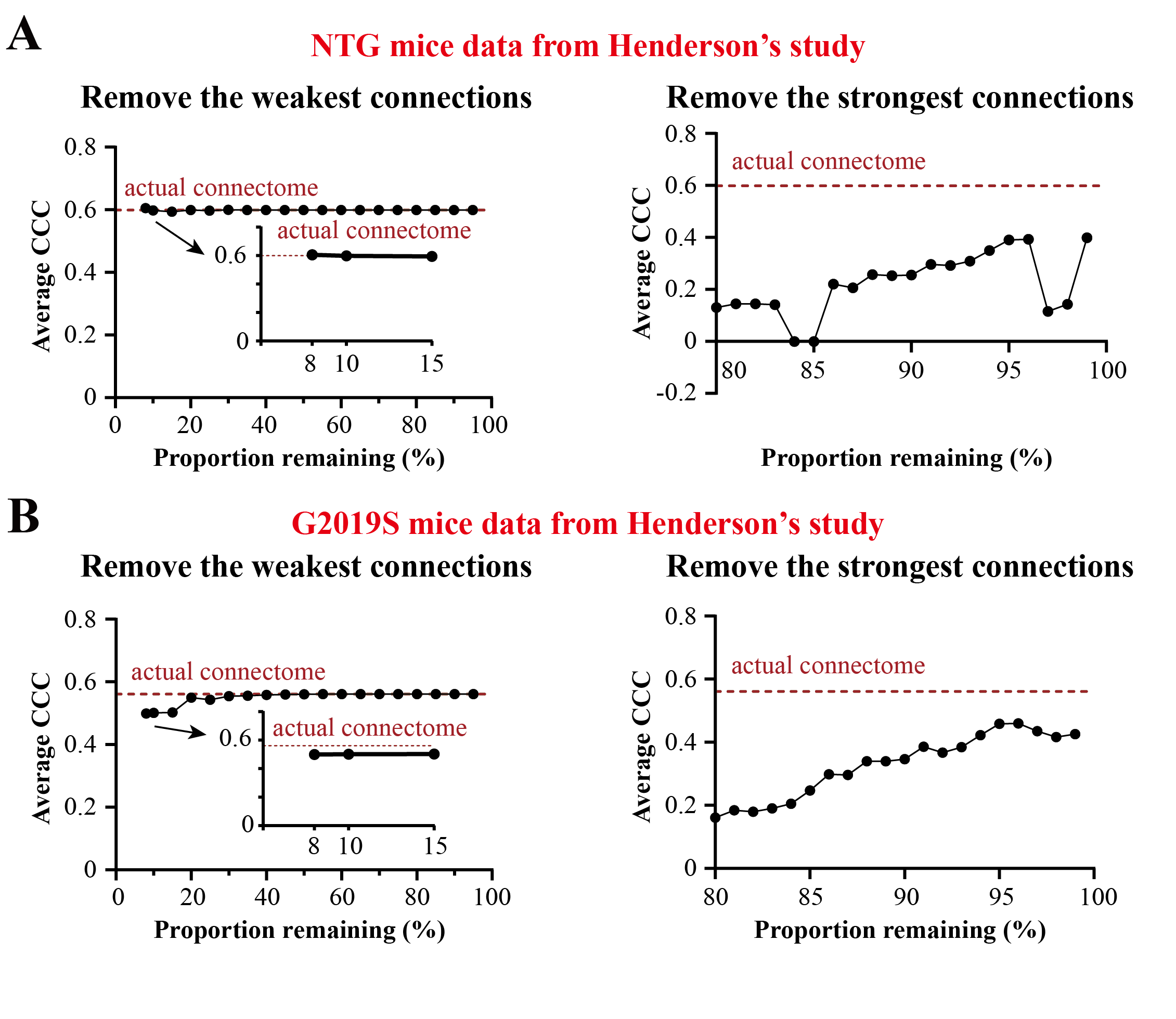


**Figure S11** The spread of pathological α-Syn was driven by the partial ‘masked’ connectome composed of the strongest connections for WT mice and *G2019S* mice. The two groups of mice data are from Henderson et al. (Henderson et al., 2019). **A**, Model performance was not affected when removing up to 92% of the weakest connections (*left*), but was markedly worse after removing the strongest 1% of connections (*right*). The dotted line of the actual connectome showed the best model fitting of the global spread model with the actual ‘masked’ connectome (Ave. CCC = 0.599). **B**, Model fitting results after removing proportions of edges from the connectome in order of connection strength using *G2019S* mouse data from Henderson et al., similar to **A**. The dotted line of the actual connectome showed the best model fitting of the global spread model with the actual ‘masked’ connectome (Ave. CCC = 0.561).


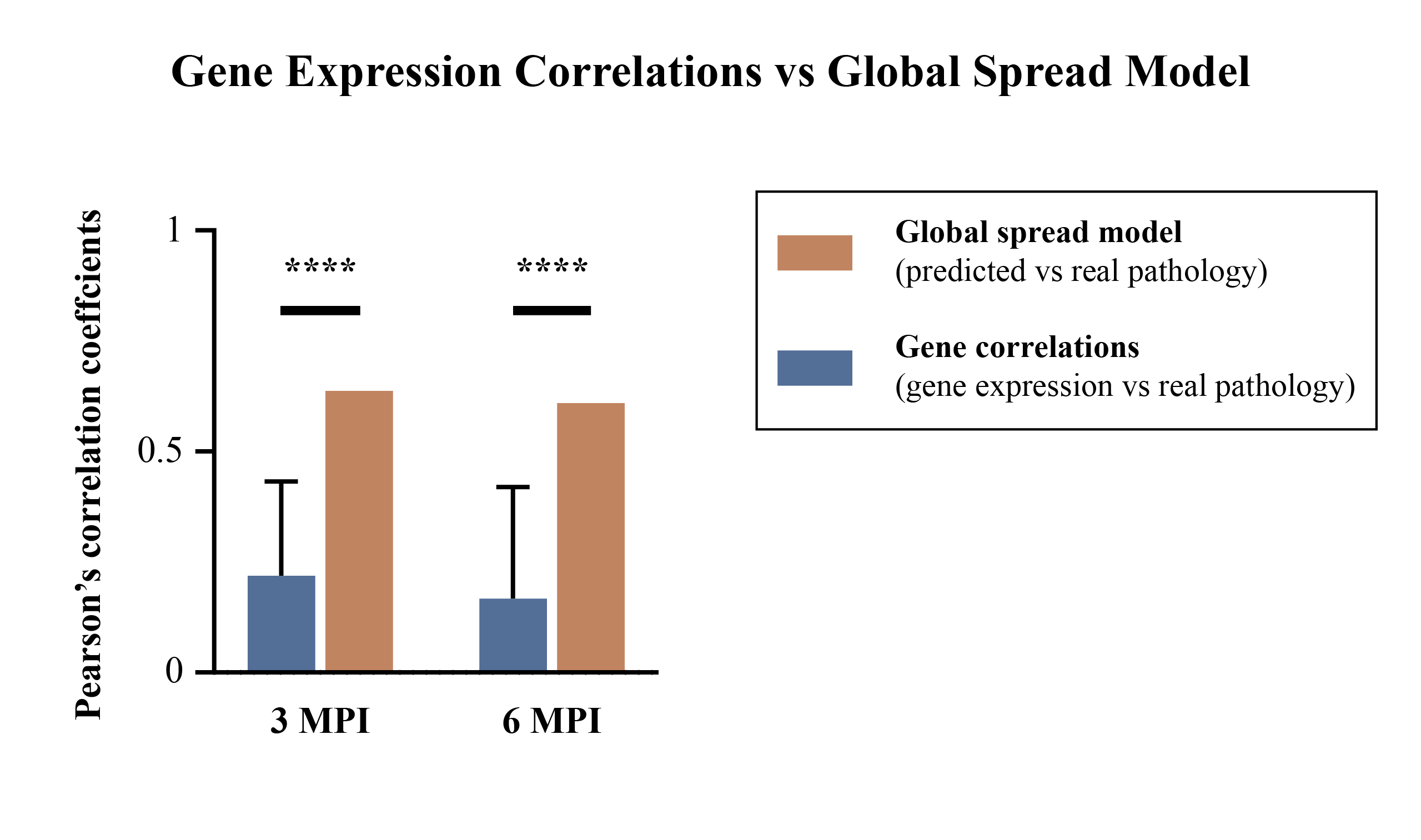


**Figure S12** Comparisons of gene expression correlations (defined as the Pearson’s correlation coefficients of gene expression and pathology) and global spread model (the Pearson’s correlation coefficients of model-predicted pathology and real pathology) at 3 and 6 MPI. Gene expression correlations had statistically worse performance than global spread model at both 3 MPI (*p*<0.0001) and 6 MPI (*p*<0.0001) with one sample Wilcoxon test. ****, *p*<0.0001. Data of gene expression correlations were shown as mean±s.d.


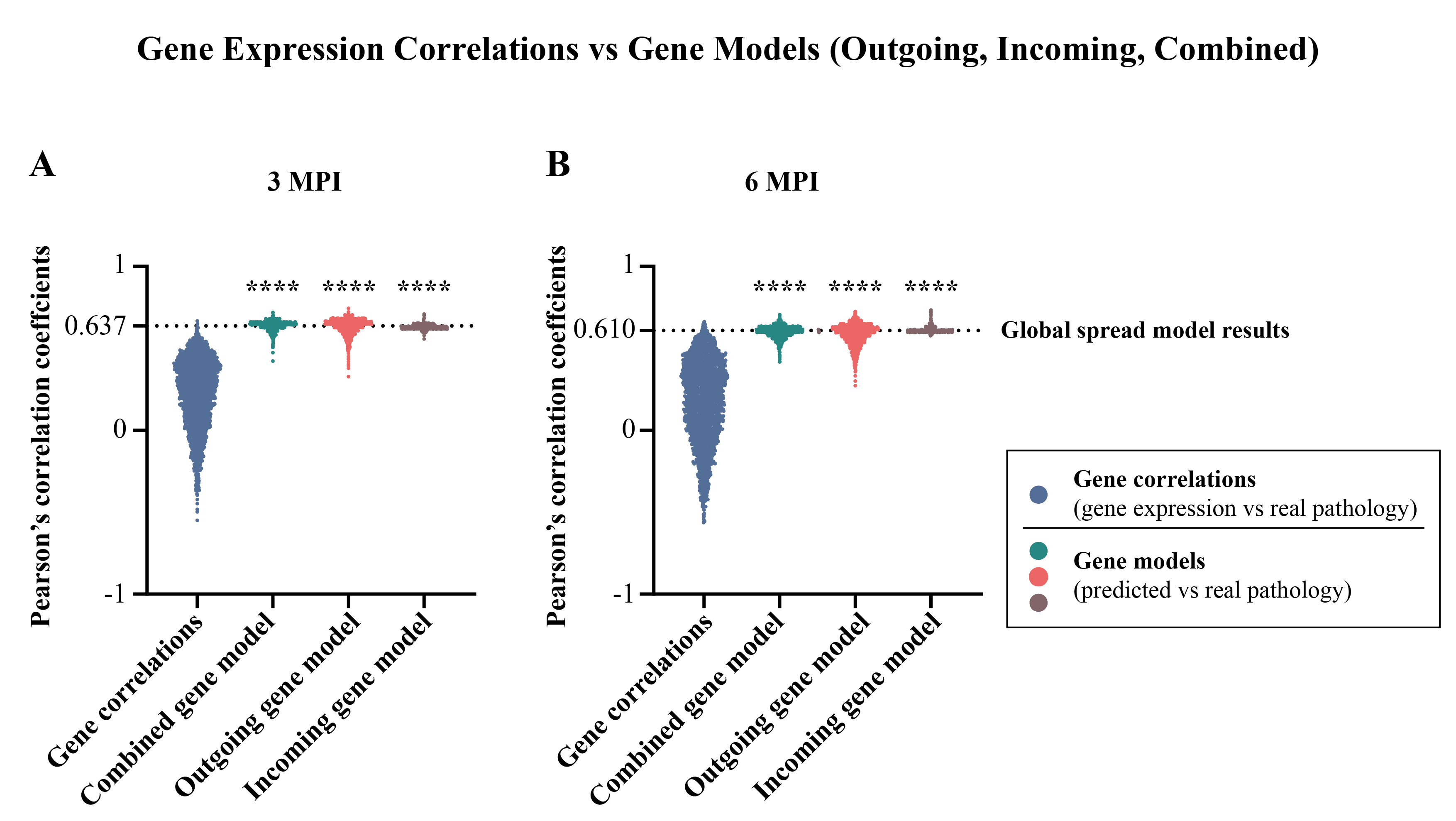


**Figure S13** Comparisons of gene expression correlations (defined as the Pearson’s correlation coefficients of gene expression and pathology) and gene models (the Pearson’s correlation coefficients of model-predicted pathology and real pathology; for outgoing, incoming, and combined effects) at 3 (**A**) and 6 (**B**) MPI. Gene expression correlations had statistically worse performance than either of the three gene models at both 3 MPI (*p*<0.0001) and 6 MPI (*p*<0.0001) with Friedman test with Dunn's multiple comparisons. Each dot in figures represents the result of one individual gene. The dashed lines represent the Pearson’s correlation coefficients of global spread model. ****, *p*<0.0001.


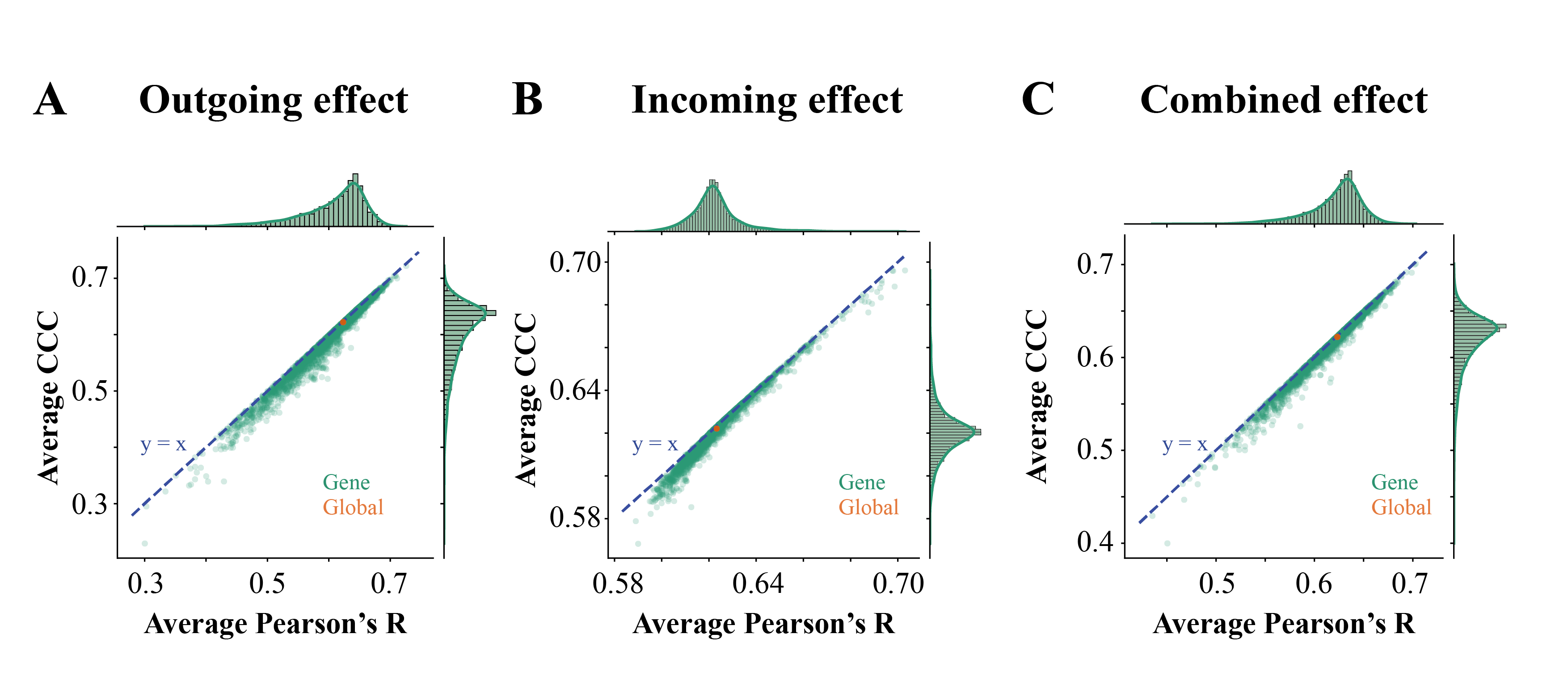


**Figure S14** The Ave. CCC vs. the Pearson’s correlation coefficient. Each green dot represents the result of one individual gene and the orange dot represents that of the global spread model. The x-axis and y-axis represent their average of Pearson’s correlation coefficients and Ave. CCC, respectively. Since all the dots were close to the dashed line (y=x), there was not much difference between the Pearson’s correlation coefficient and CCC, revealing the gene model worked well in capturing the scale features. **A**, Outgoing effect. **B**, Incoming effect. **C**, Combined effect.


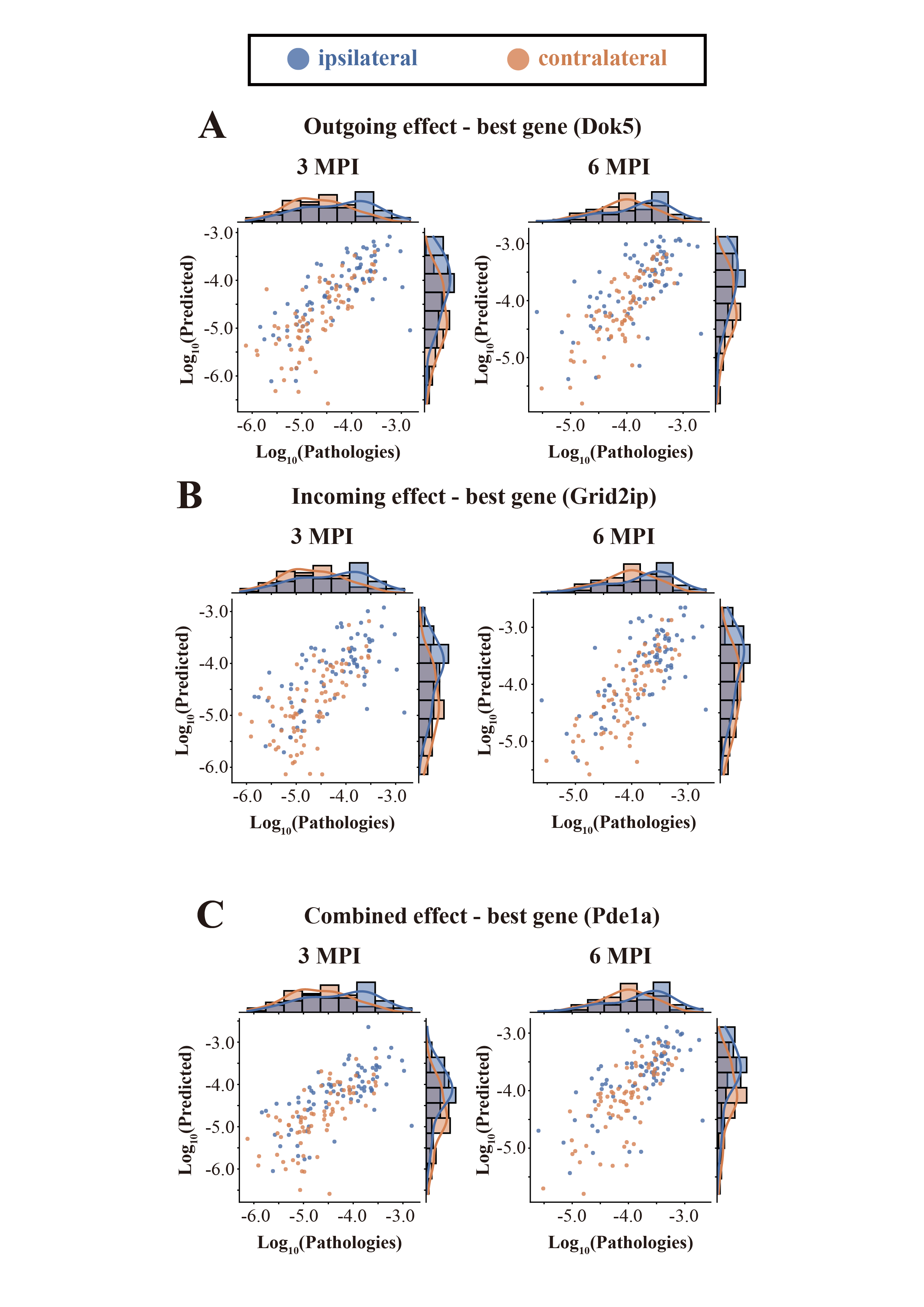


**Figure S15** The gene expression model also accurately captures the feature that the ipsilateral hemisphere had more pathology than the contralateral hemisphere both at 3 and 6 MPI. Regions ipsilateral to the injection site are shown in blue, while contralateral regions are shown in orange, with histograms of the univariate distributions shown along the axes. **A**, Best outgoing effect (*Dok5*). **B**, Best incoming effect gene (*Grid2ip*). **C**, Best combined effect gene (*Pde1a*).


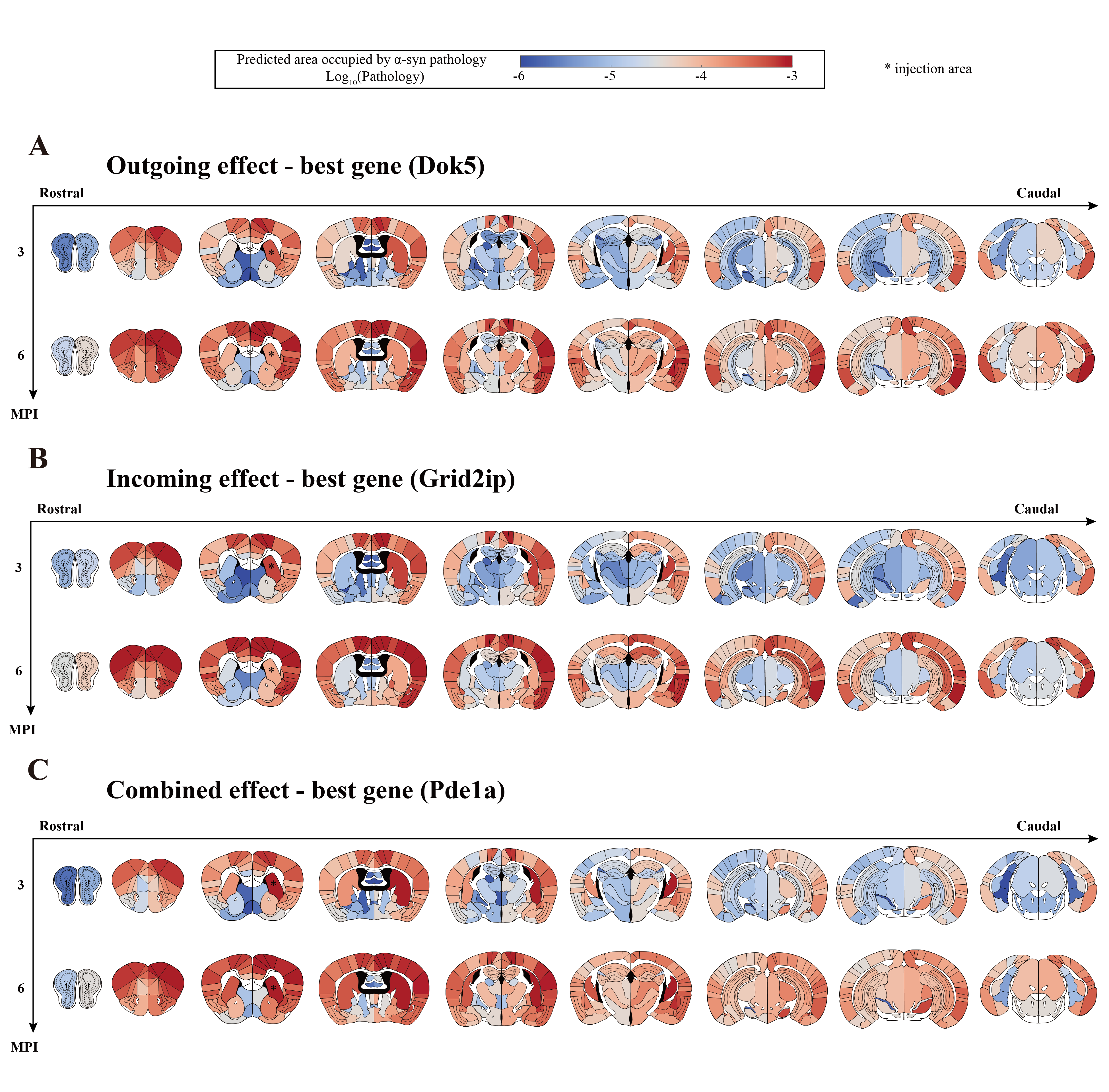


**Figure S16** Heatmap of the regional α-Syn pathology predicted by the best gene model (log10-transformed; *, injection area) for **A**, outgoing effect (*Dok5*); **B**, incoming effect (*Grid2ip*). **C**, combined effect (*Pde1a*).


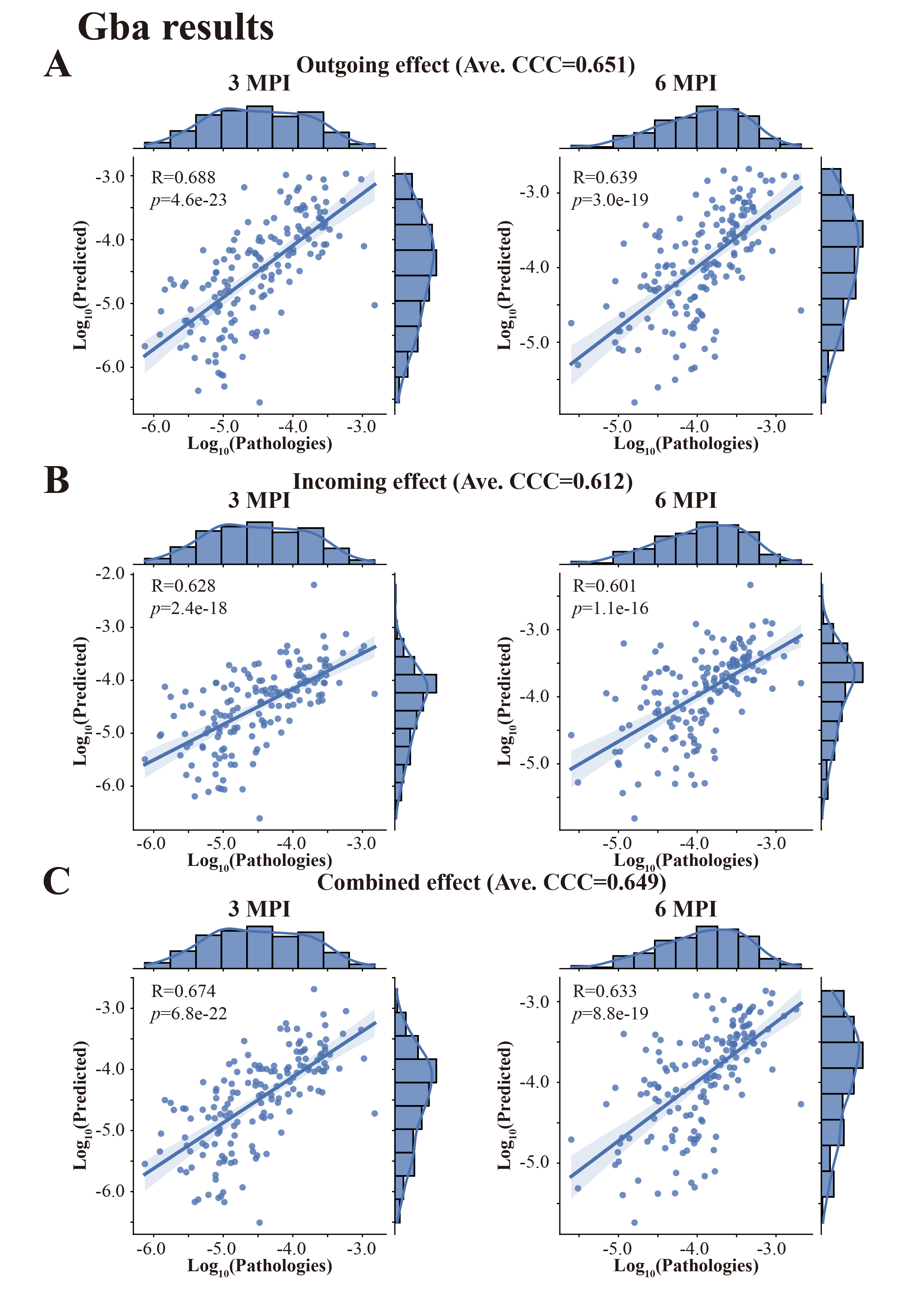


**Figure S17** Model results of *Gba* for outgoing (**A**), incoming (**B**), and combined effects (**C**), respectively. Each dot represents one brain region, while the x-axis and y-axis represent the pathology (log10-transformed) found empirically and predicted by the model, respectively. The Pearson’s correlation coefficient and the best regression lines for both 3 and 6 MPI are also displayed. The shaded ribbon represents the 95% prediction interval. Abbreviations: R, Pearson’s correlation coefficient; *p*, *p* values from linear regression.


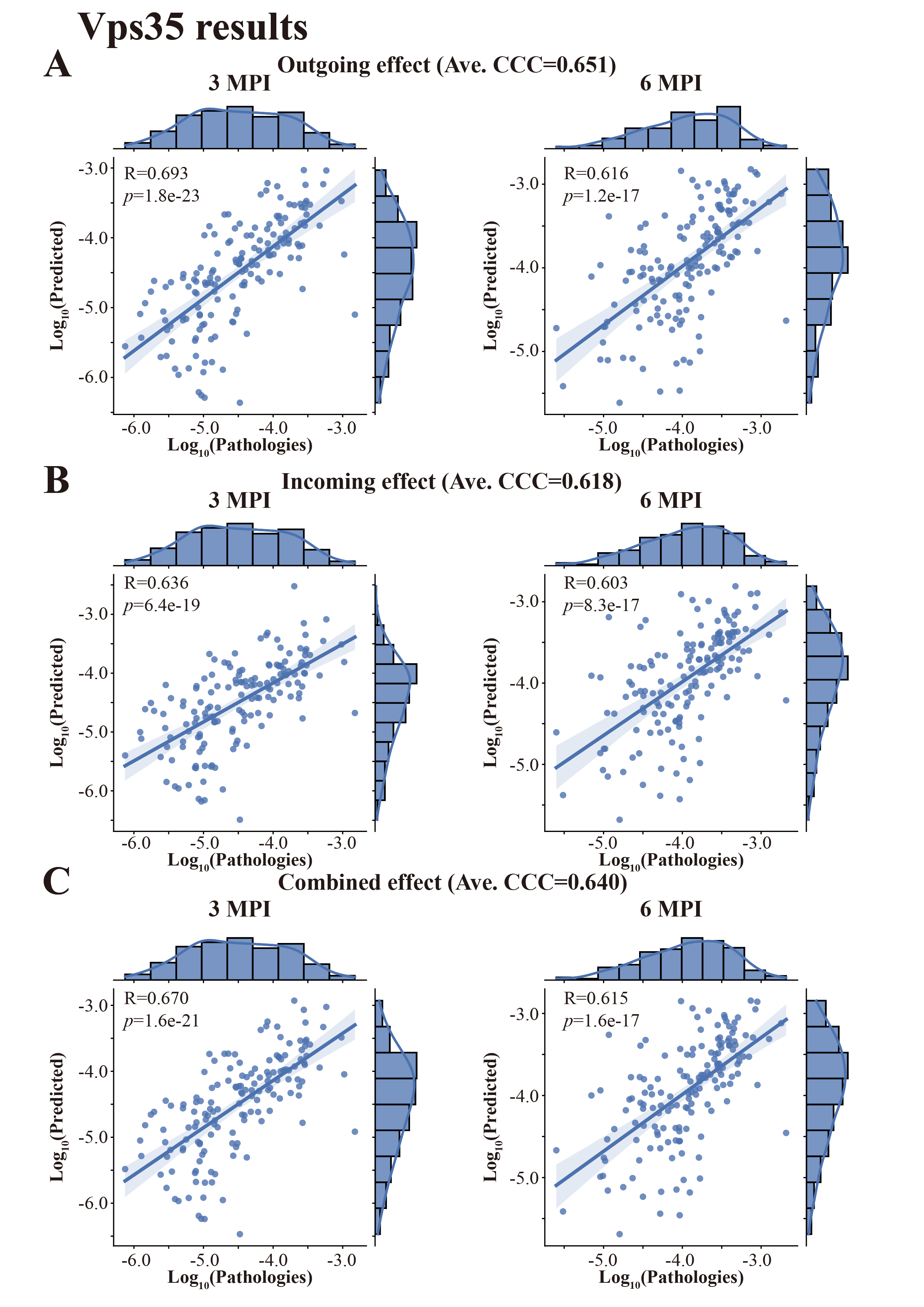


**Figure S18** Model results of *Vps35* for outgoing (**A**), incoming (**B**), and combined effect (**C**), respectively. Each dot represents one brain region, while the x-axis and y-axis represent the pathology (log10-transformed) found empirically and predicted by the model, respectively. The Pearson’s correlation coefficient and the best regression lines for both 3 and 6 MPI are also displayed. The shaded ribbon represents the 95% prediction interval. Abbreviations: R, Pearson’s correlation coefficient; *p*, *p* values from linear regression.


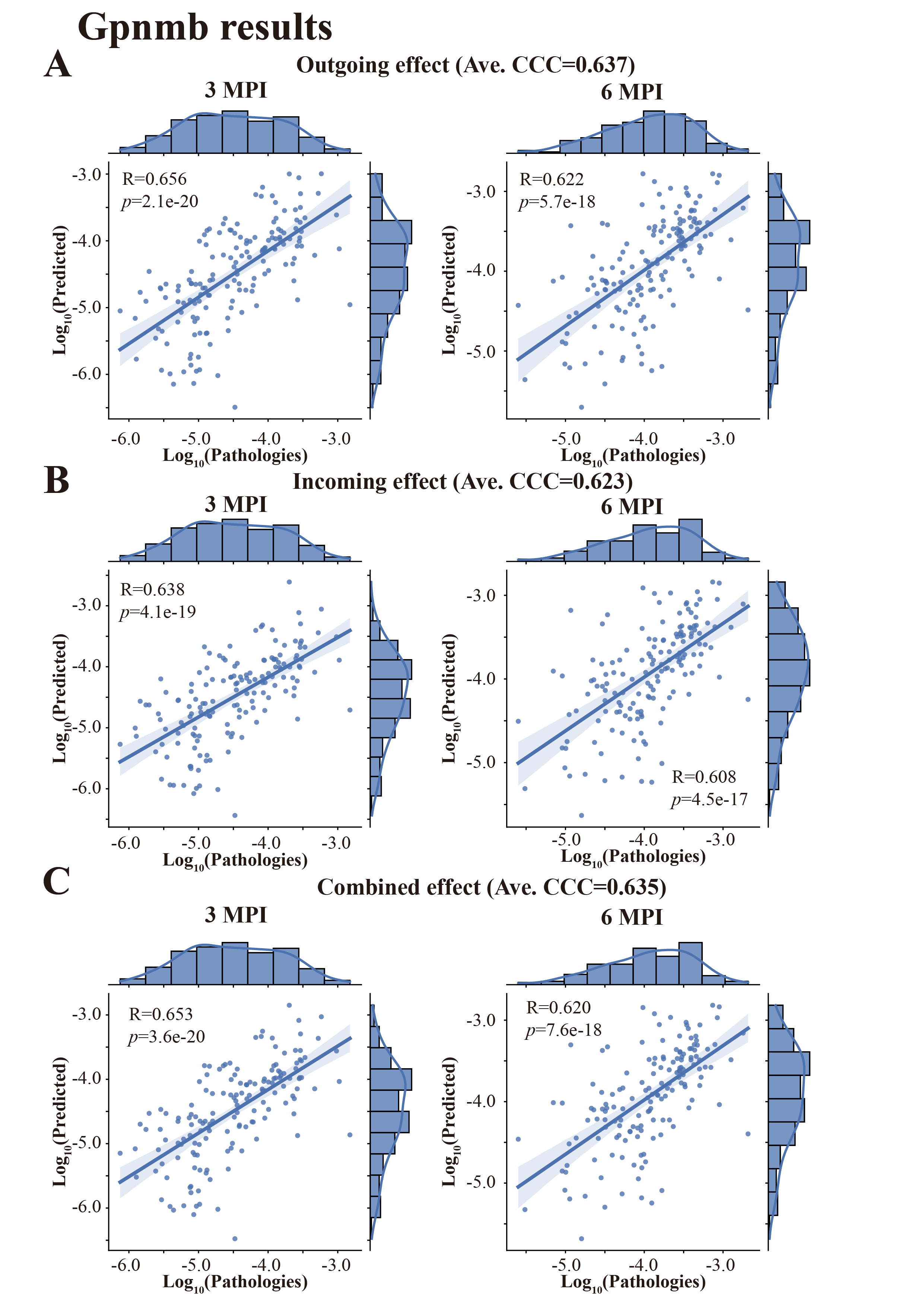


**Figure S19** Model results of *Gpnmb* for outgoing (**A**), incoming (**B**), and combined effect (**C**), respectively. Each dot represents one brain region, while the x-axis and y-axis represent the pathology (log10-transformed) found empirically and predicted by the model, respectively. The Pearson’s correlation coefficient and the best regression lines for both 3 and 6 MPI are also displayed. The shaded ribbon represents the 95% prediction interval. Abbreviations: R, Pearson’s correlation coefficient; *p*, *p* values from linear regression.


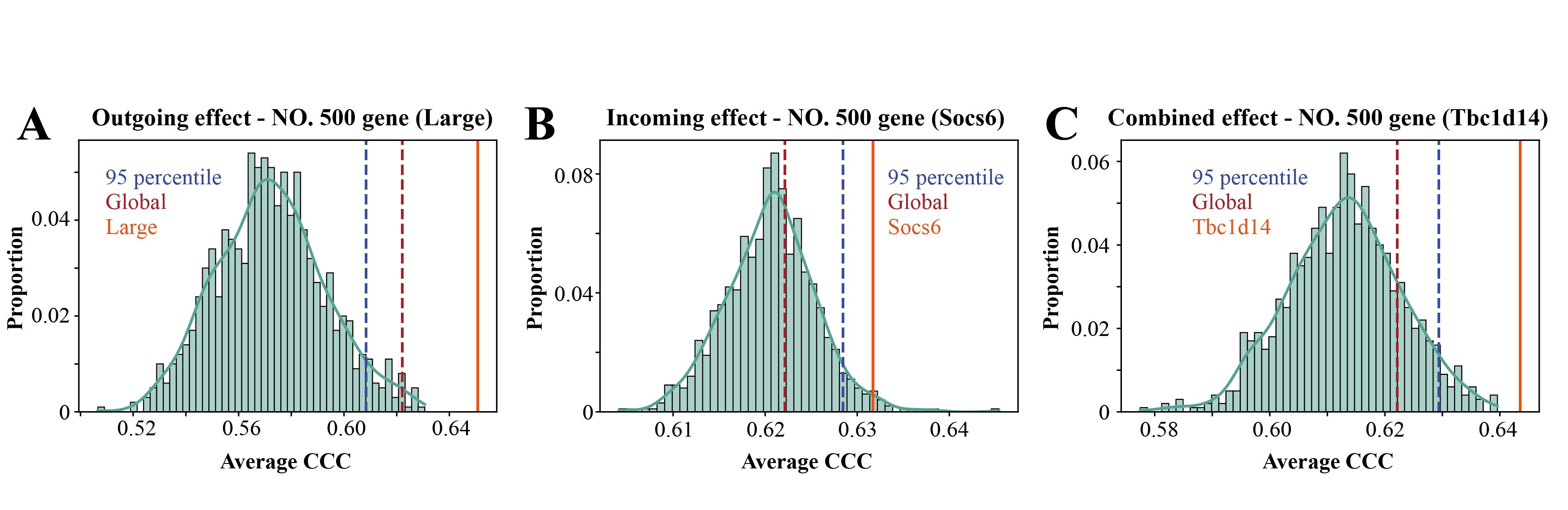


**Figure S20** Bootstrapping results with 1,000 replicates in which the elements of the 500th-best genes for each of the **A**, outgoing (*Large*); **B**, incoming (*Socs6*). **C**, combined (*Tbc1d14*) effects, respectively, were randomly permuted. The true Ave. CCC of 500th-best genes were all higher than the 95% percentiles of the null model distributions.


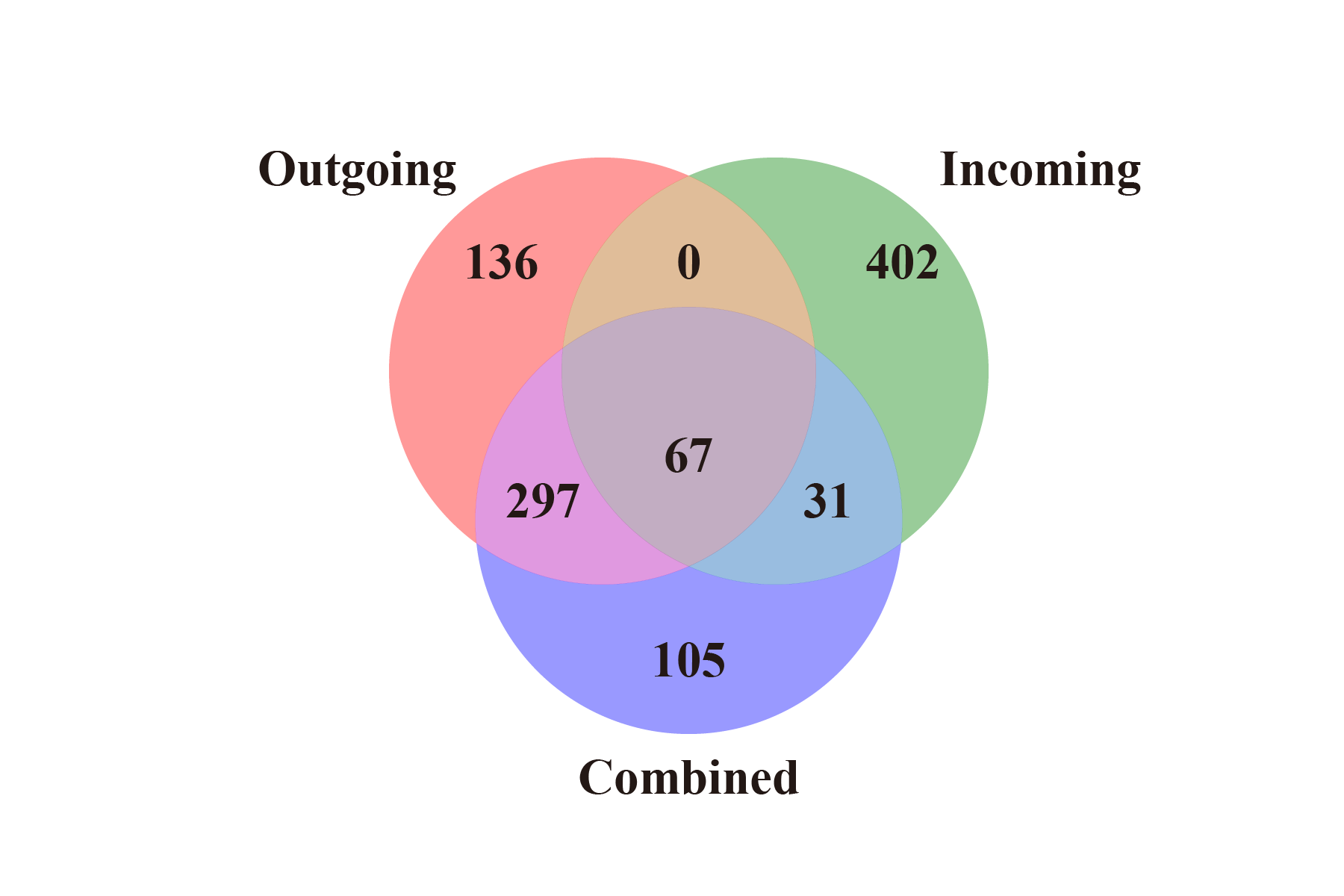


**Figure S21** Venn diagram showing the relationships among the top 500 genes of the outgoing, incoming, and combined effects. There was more gene overlap between the outgoing effect and the combined effect than the other effect pairs.


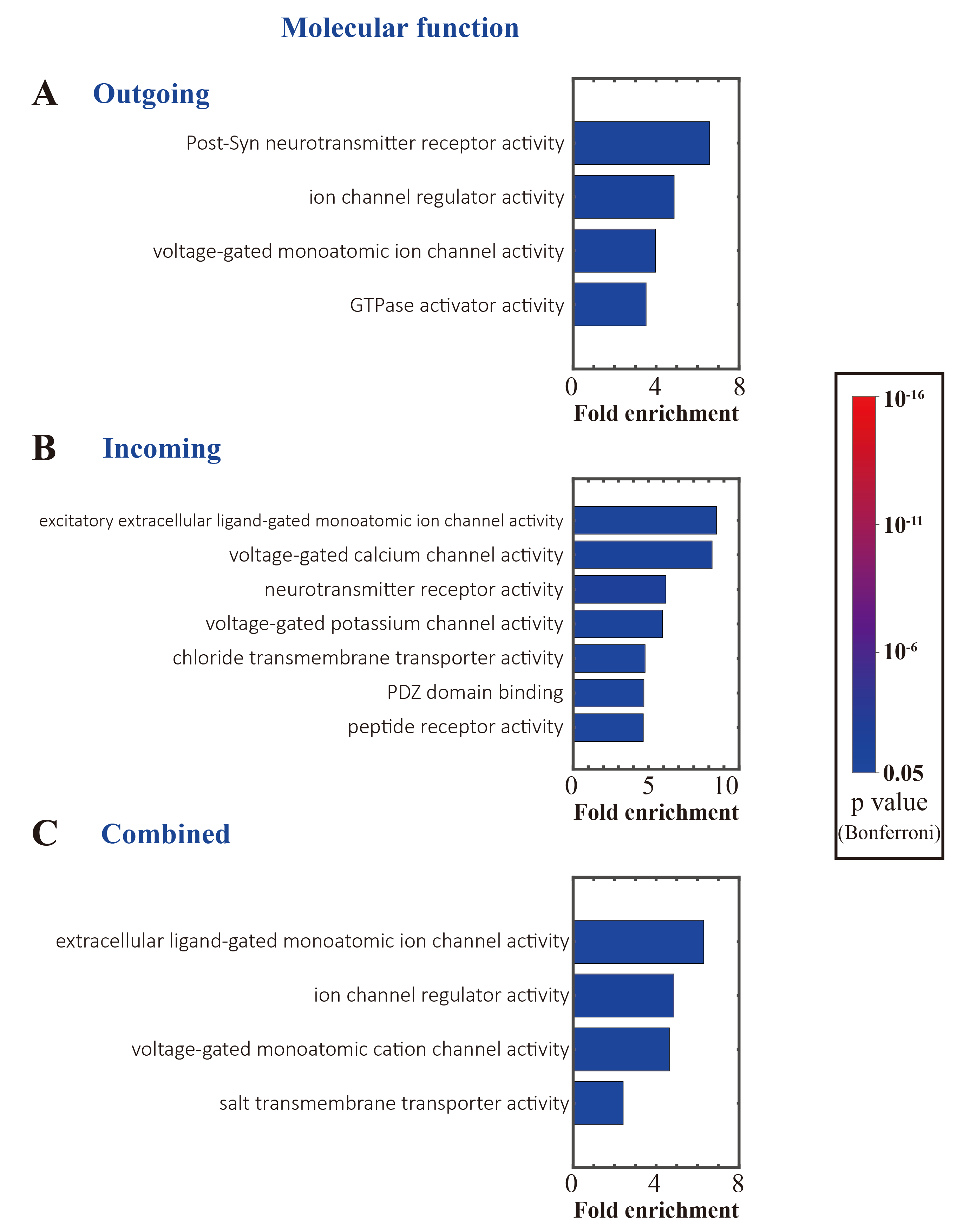


**Figure S22** Molecular function of GO analyses for the top 500 genes of outgoing, incoming, and combined effect. **A**, Outgoing effect. **B**, Incoming effect. **C**, Combined effect. For each GO entry, the displayed results were strictly sorted by the highest fold enrichment and statistically significant after Bonferroni correction. The colors of the bars represent the *p* values, and the length represents the fold enrichment. Only the primary hierarchy is shown. Abbreviations: Post-Syn, postsynaptic.


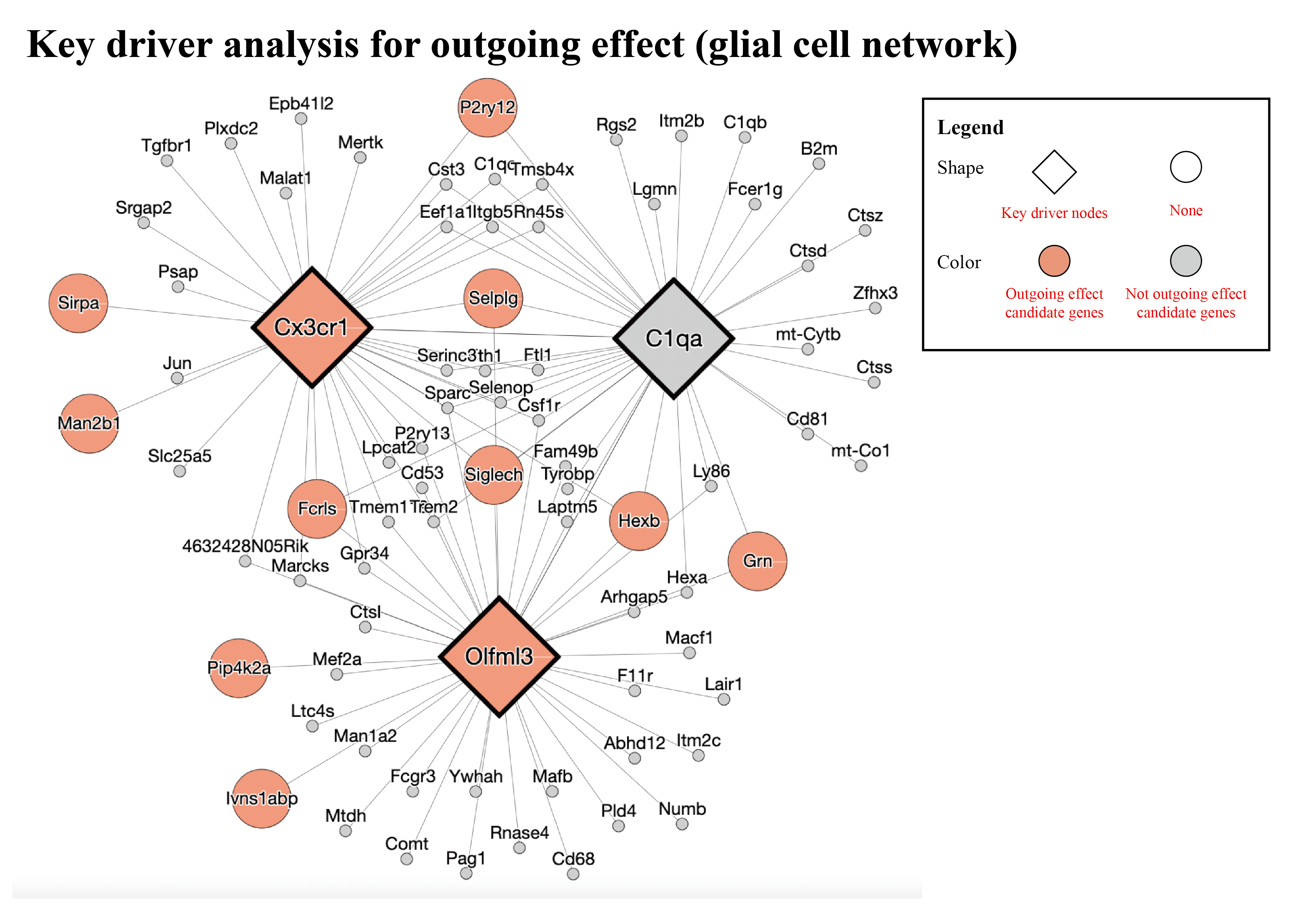


**Figure S23** KD analysis for candidate genes of incoming effect and the associated gene regulatory network on glial cell scRNAseq data. The diamond shape indicate genes that were identified to be KDs, and circles indicate not. The colors indicate whether the gene occurs in outgoing effect candidate gene subset or not. The SCING neuronal network is constructed using scRNAseq data from Tabula Muris, Tabula Muris Senis, and Mouse Cell Atlas, with networks built on the Allen 10X dataset from the hippocampus, visual cortex, somatosensory cortex, and primary motor cortex brain regions.


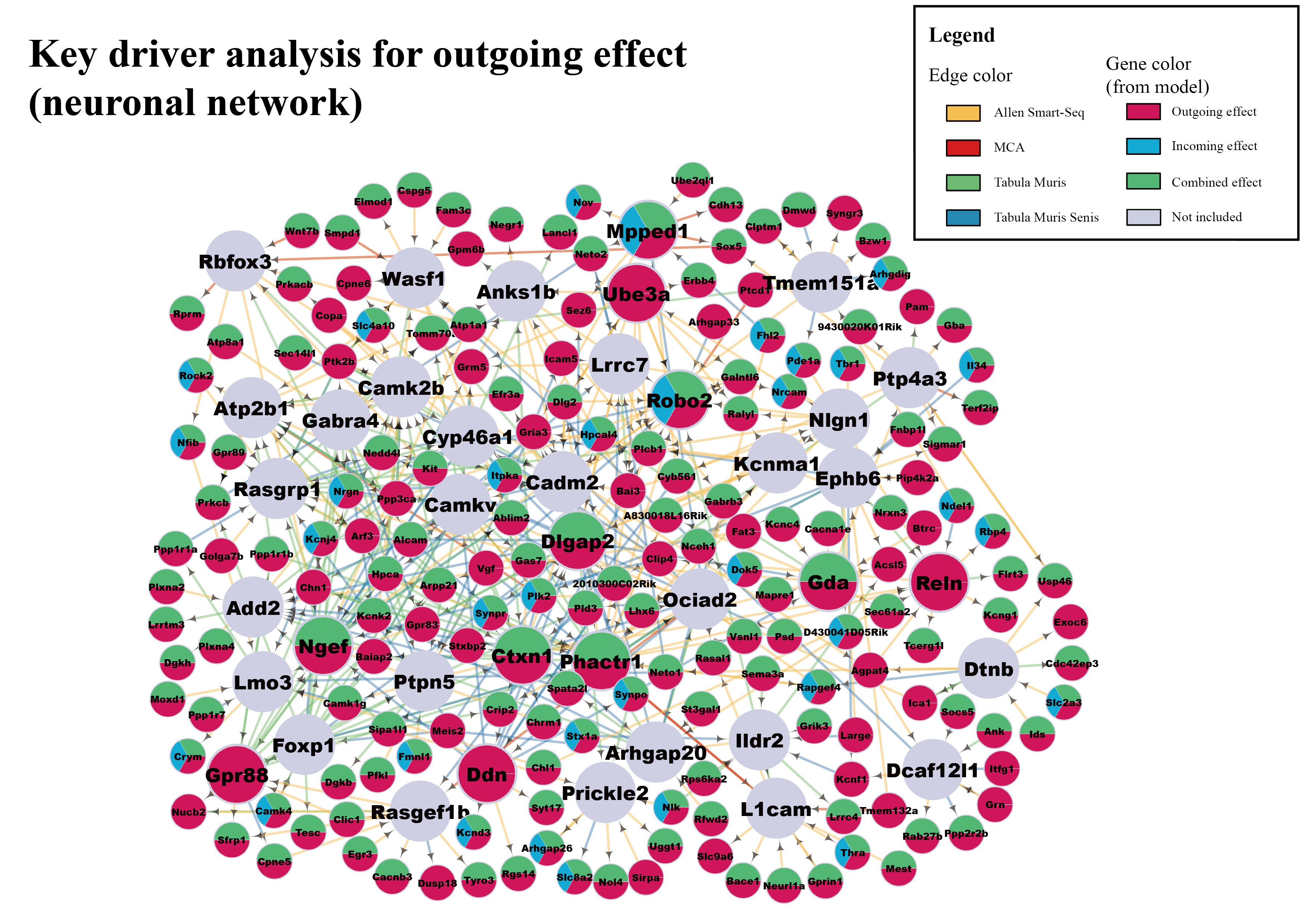


**Figure S24** KD analysis for candidate genes of outgoing effect and the associated gene regulatory network on neuronal scRNAseq data. The larger circles indicate genes that were identified to be KDs, and smaller circles indicate candidate genes from the outgoing effects. The direction of edges indicates the regulatory relationship between genes in the SCING neuronal network constructed using scRNAseq data from single cell atlases Allen Smart-seq, mouse cell atlas (MCA), Tabula Muris, and Tabula Muris Senis. The color inside the circle denotes whether the gene has outgoing (red), incoming (green), or combined (blue) effect, while the multiple colors indicate the gene is from multiple categories. KD genes that did not appear in any of the effects were marked as gray color. Note that network KDs for the candidate genes do not have to be candidate genes themselves but are predicted to regulate the candidate genes.


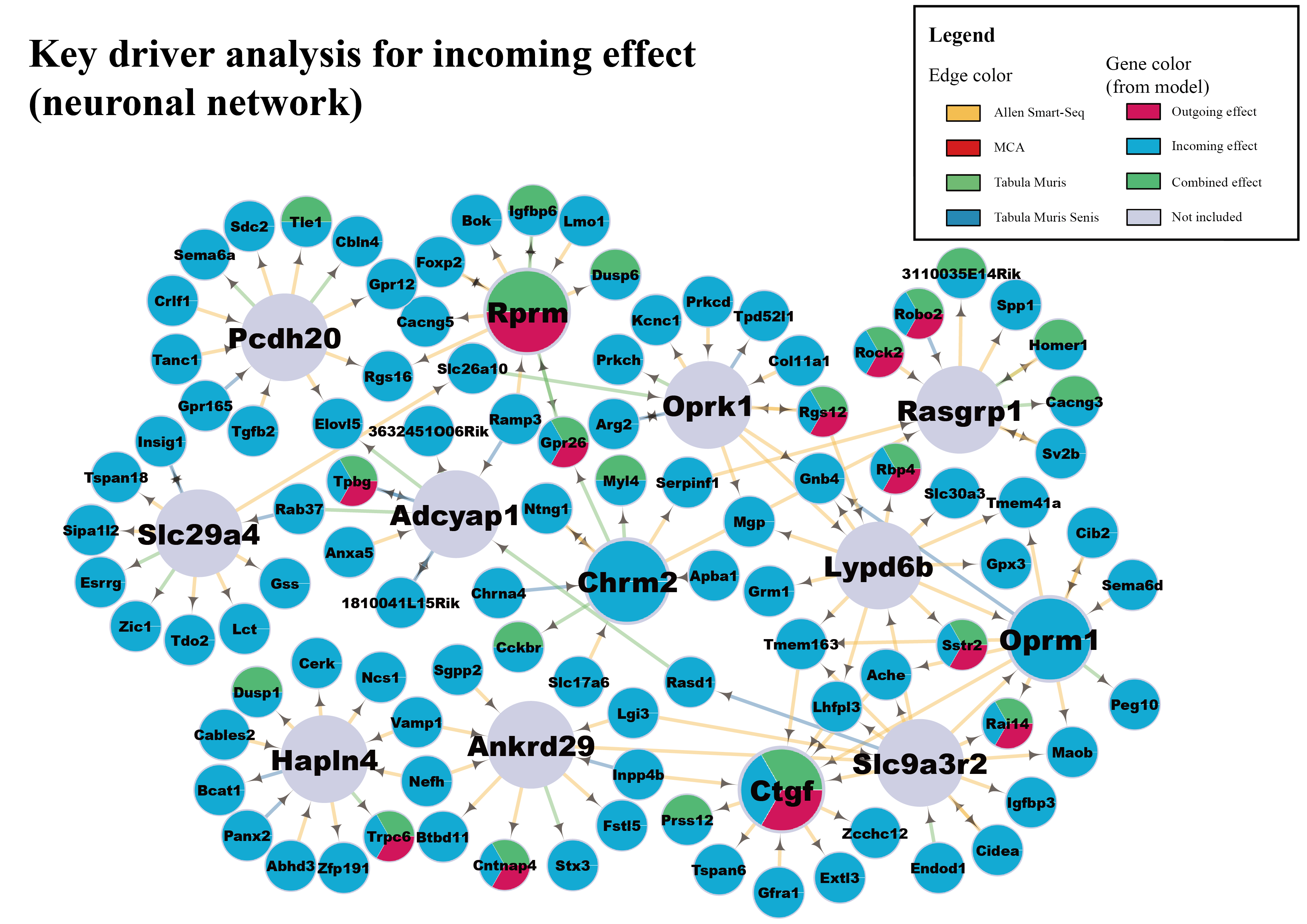


**Figure S25** KD analysis for candidate genes of incoming effect and the associated gene regulatory network on neuronal scRNAseq data. The larger circles indicate genes that were identified to be KDs, and smaller circles indicate candidate genes from the incoming effects. The direction of edges indicates the regulatory relationship between genes in the SCING neuronal network constructed using scRNAseq data from single cell atlases Allen Smart-seq, mouse cell atlas (MCA), Tabula Muris, and Tabula Muris Senis. The color inside the circle denotes whether the gene has outgoing (red), incoming (green), or combined (blue) effect, while the multiple colors indicate the gene is from multiple categories. KD genes that did not appear in any of the effects were marked as gray color. Note that network KDs for the candidate genes do not have to be candidate genes themselves but are predicted to regulate the candidate genes.


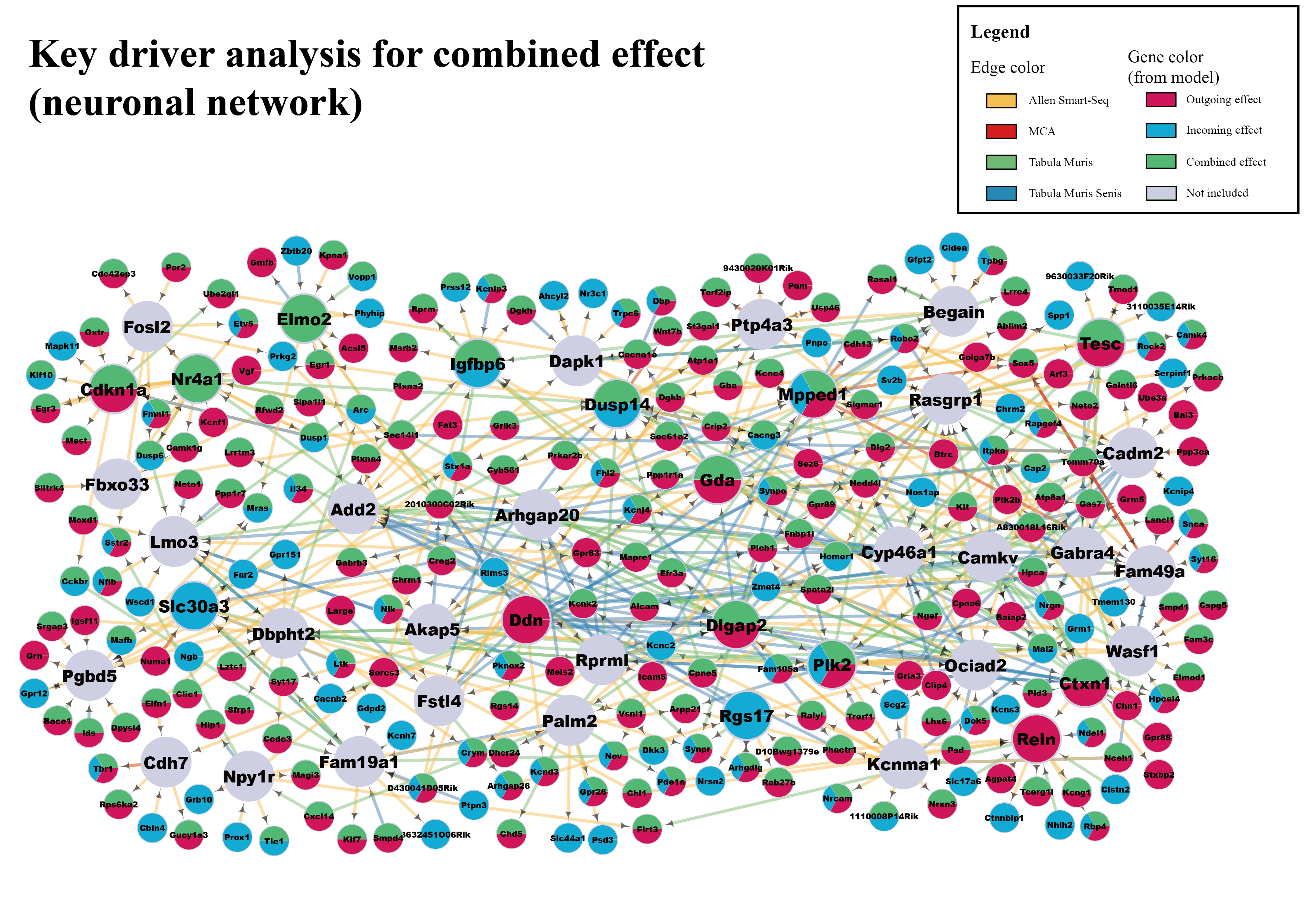


**Figure S26** KD analysis for candidate genes of combined effect and the associated gene regulatory network on neuronal scRNAseq data. The larger circles indicate genes that were identified to be KDs, and smaller circles indicate candidate genes from the combined effects. The direction of edges indicates the regulatory relationship between genes in the SCING neuronal network constructed using scRNAseq data from single cell atlases Allen Smart-seq, mouse cell atlas (MCA), Tabula Muris, and Tabula Muris Senis. The color inside the circle denotes whether the gene has outgoing (red), incoming (green), or combined (blue) effect, while the multiple colors indicate the gene is from multiple categories. KD genes that did not appear in any of the effects were marked as gray color. Note that network KDs for the candidate genes do not have to be candidate genes themselves but are predicted to regulate the candidate genes.


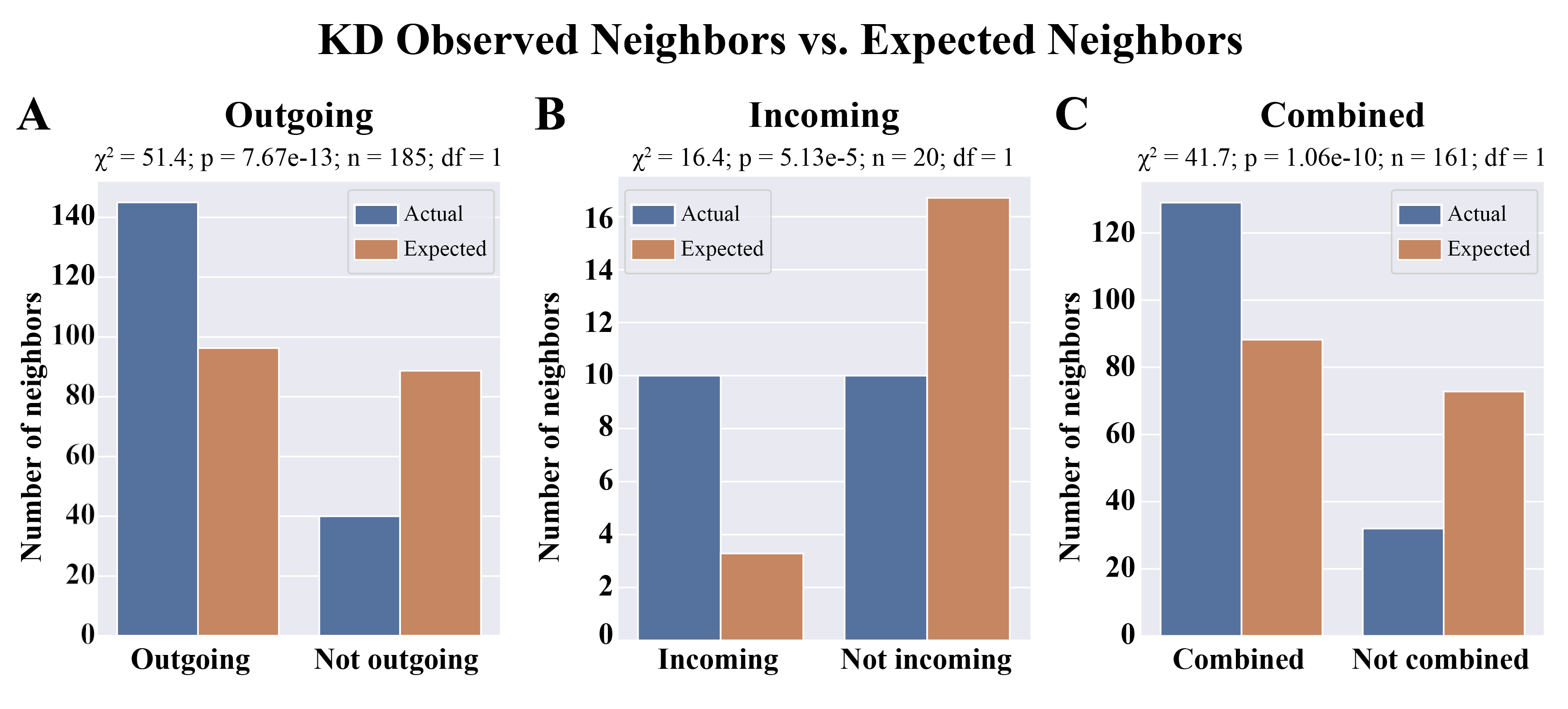


**Figure S27** Chi-square goodness of fit test for number of neighbors of each category for the union regulatory network. A, Outgoing subset. B, Incoming subset. C, Combined subset.

**Reference**

Henderson, M.X., E.J. Cornblath, A. Darwich, B. Zhang, H. Brown, R.J. Gathagan, R.M. Sandler, D.S. Bassett, J.Q. Trojanowski, and V.M. Lee. 2019. Spread of α-synuclein pathology through the brain connectome is modulated by selective vulnerability and predicted by network analysis. *Nature neuroscience* 22:1248-1257.
